# Supplementary figures and images for: Objective perimetry identifies regional functional progression and recovery in mild Diabetic Macular Oedema
Source: PLoS One. 2023 Jun 15;18(6):e0287319. doi: 10.1371/journal.pone.0287319 (PMC10270604; doi:10.1371/journal.pone.0287319)

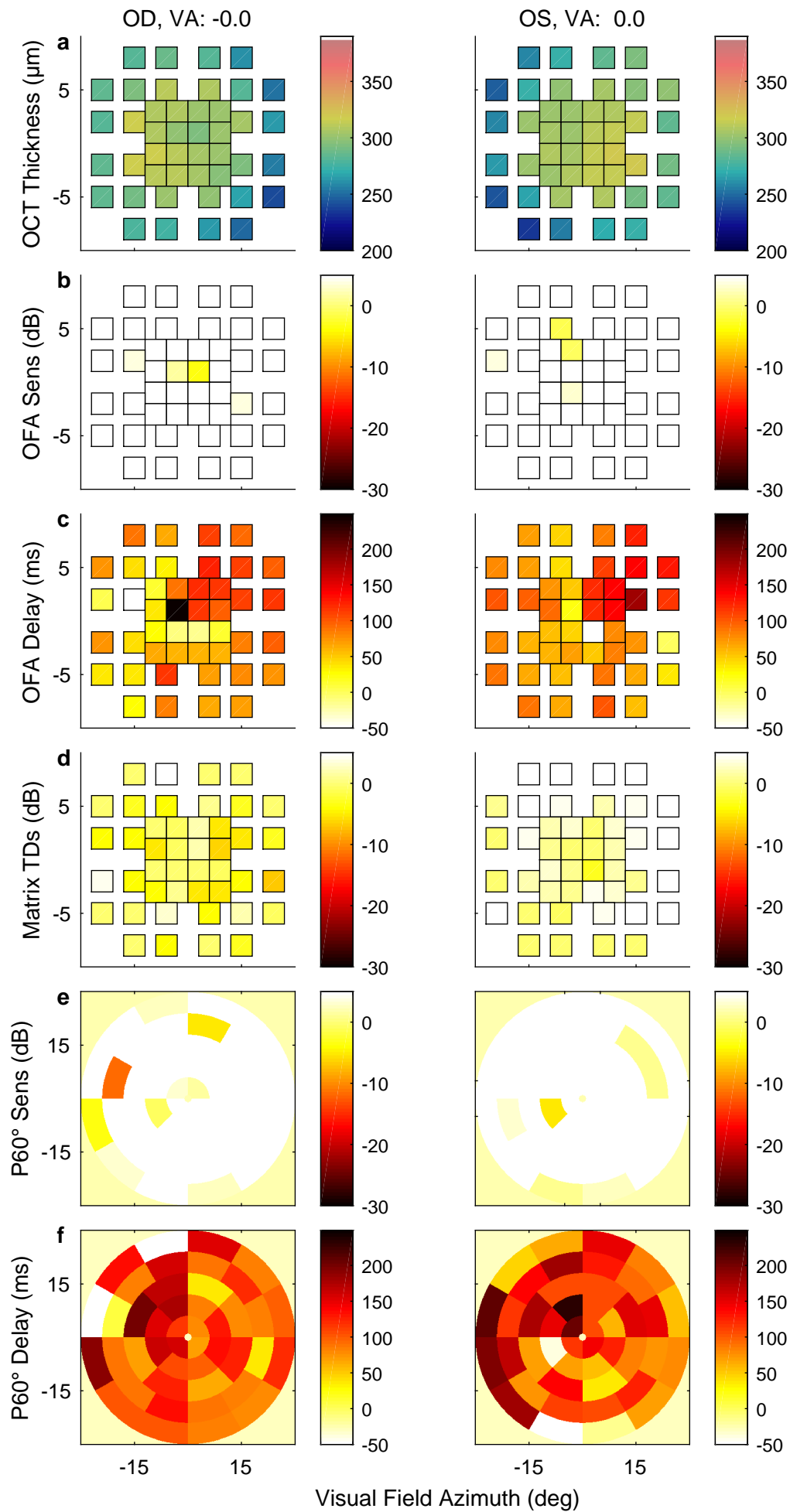

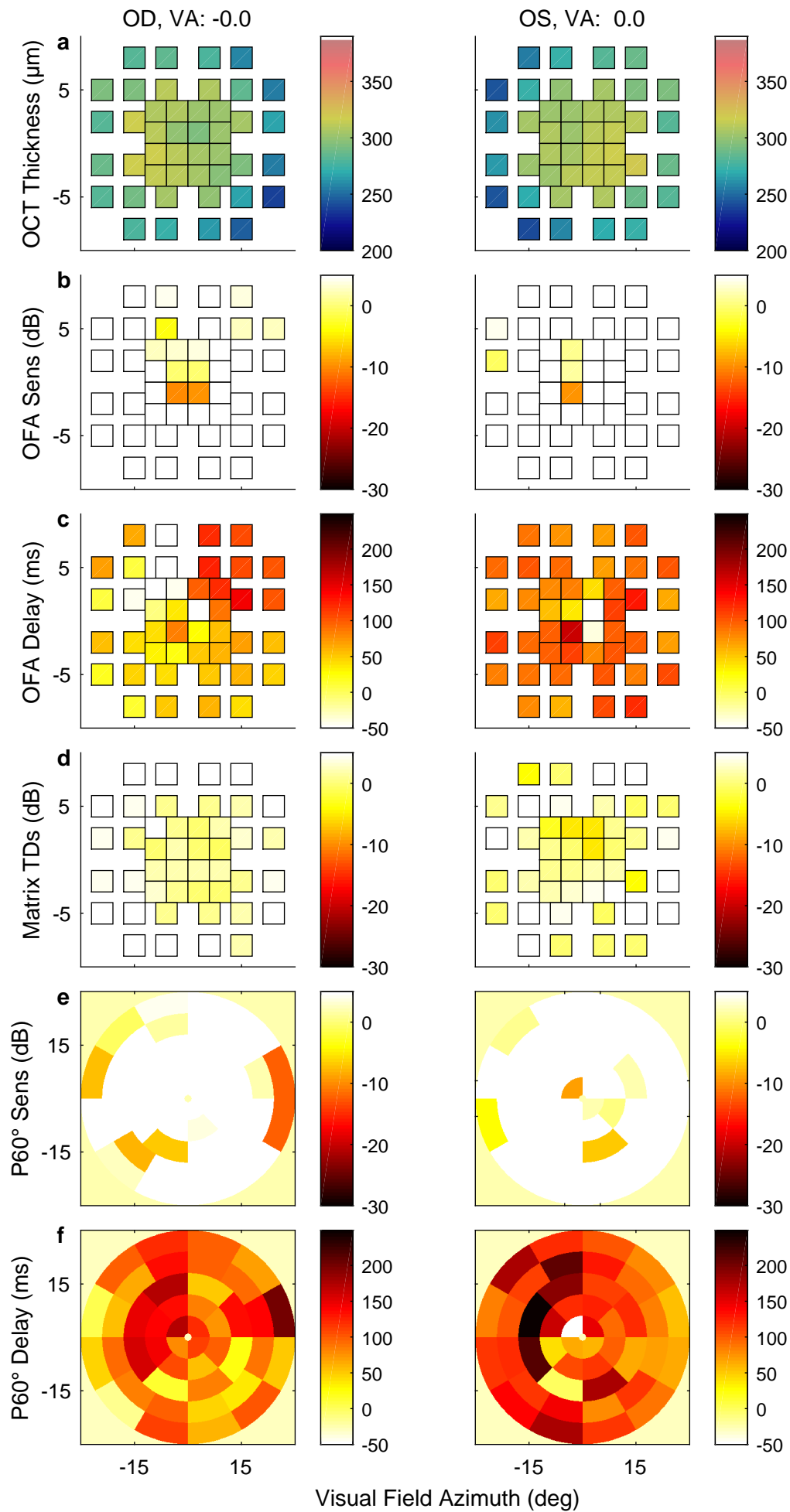

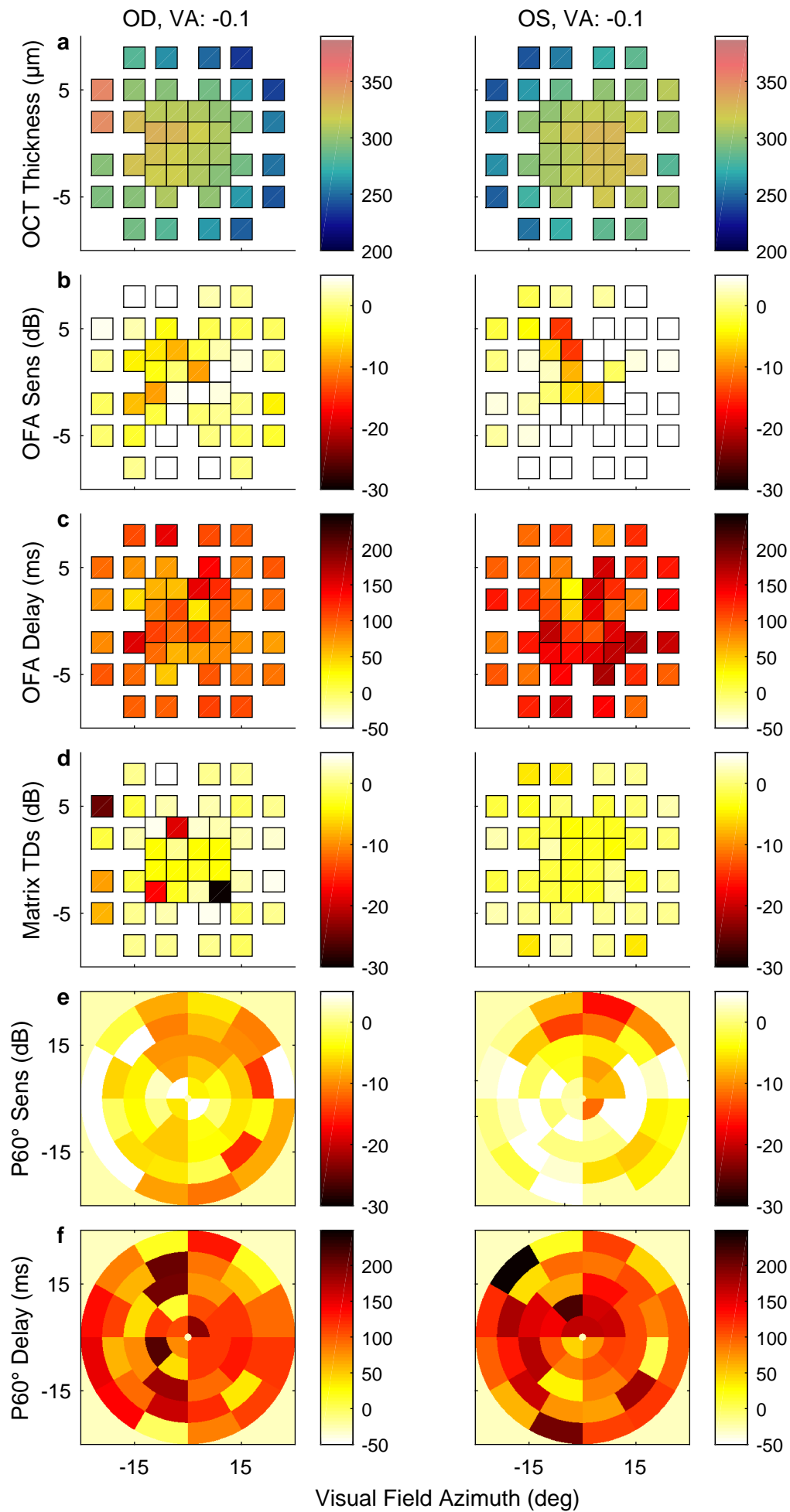

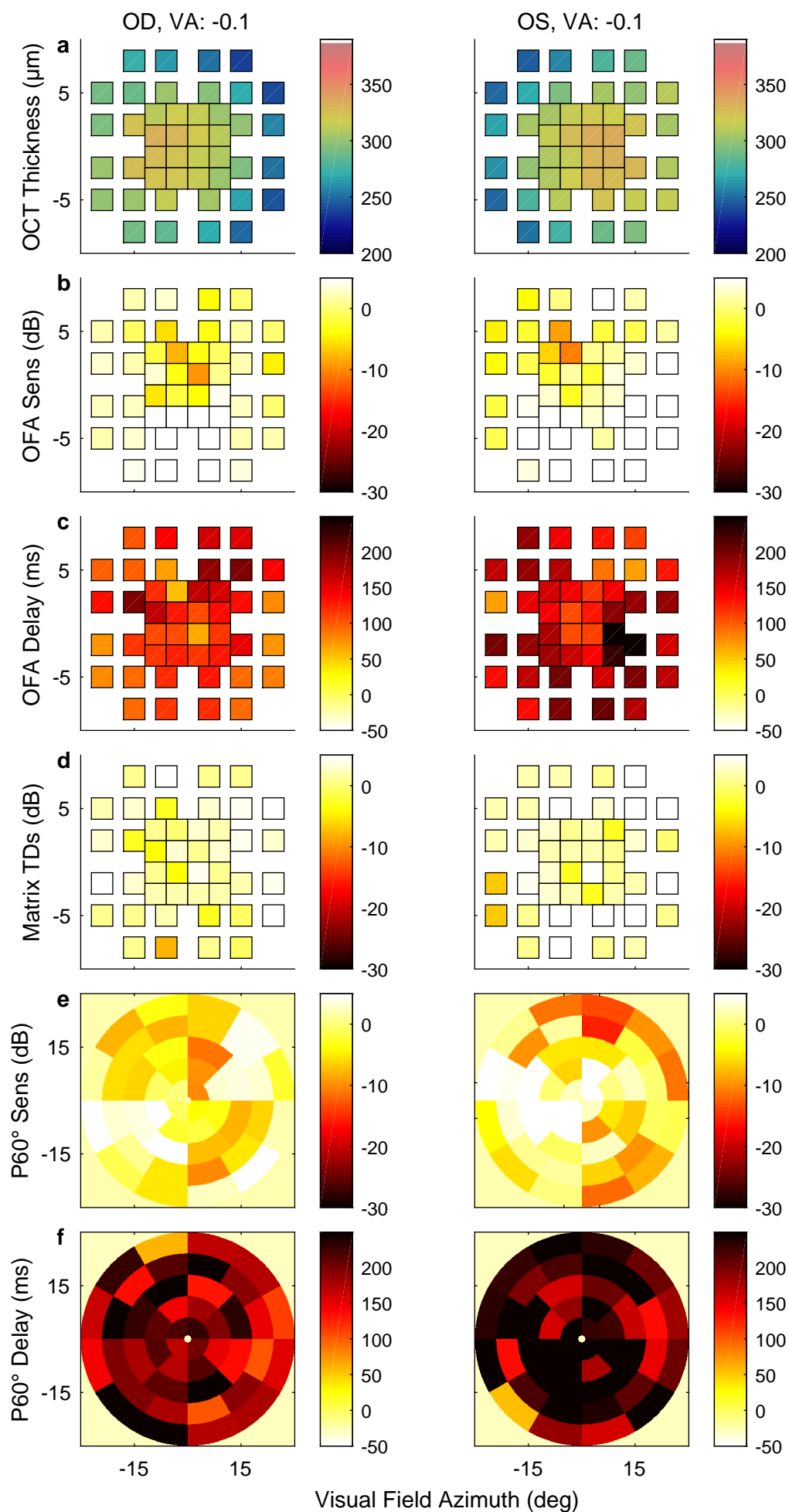

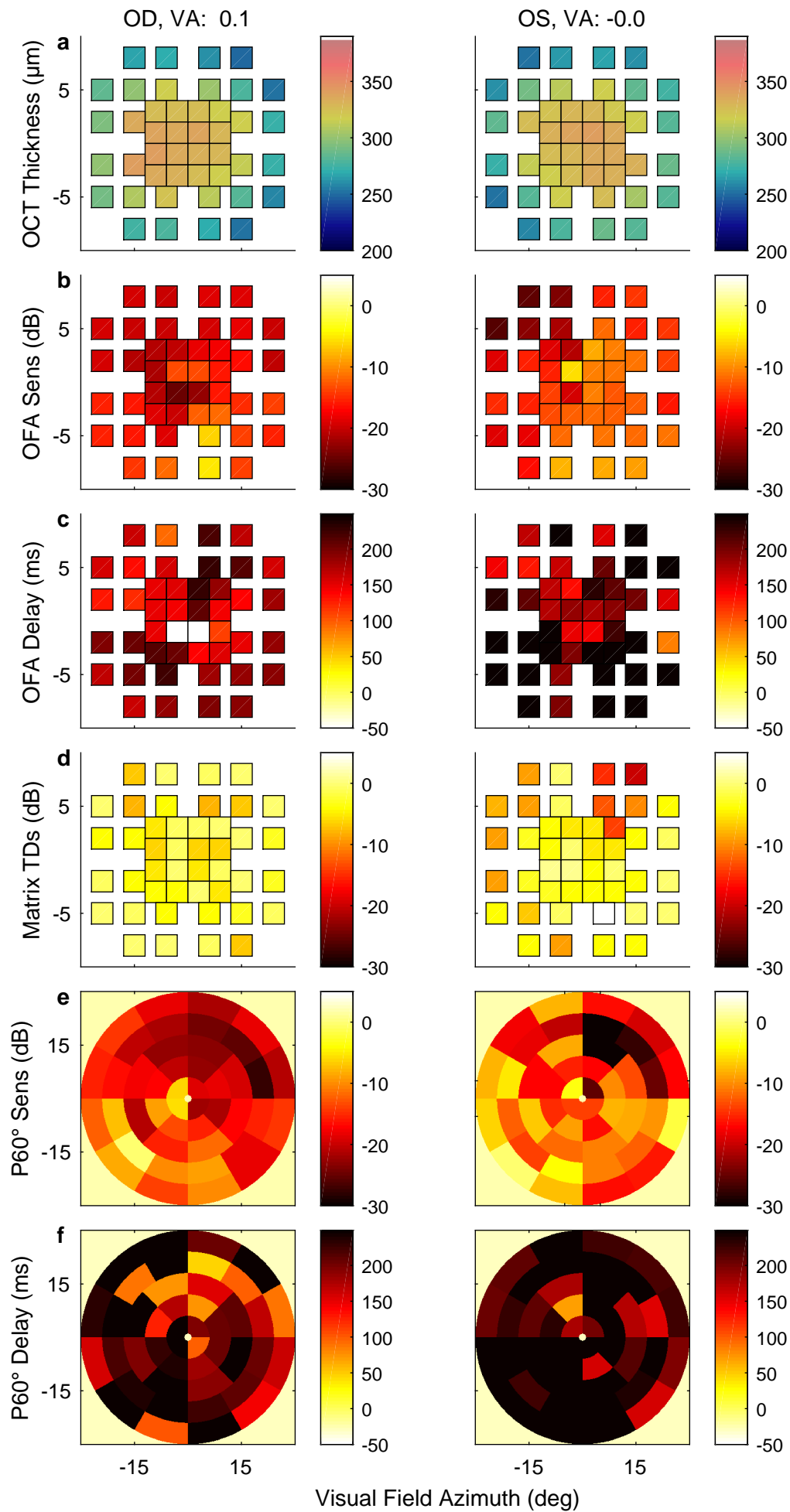

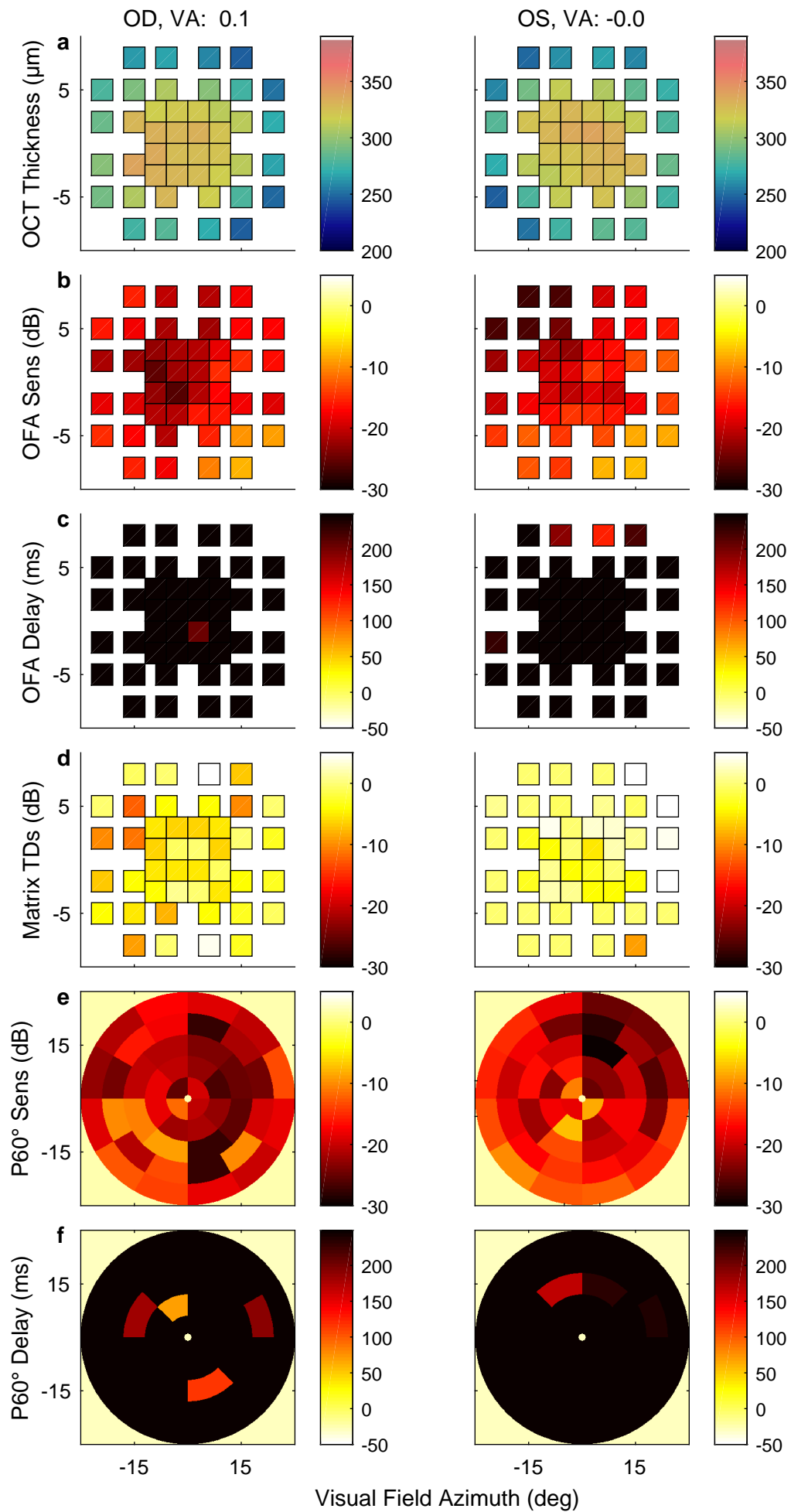

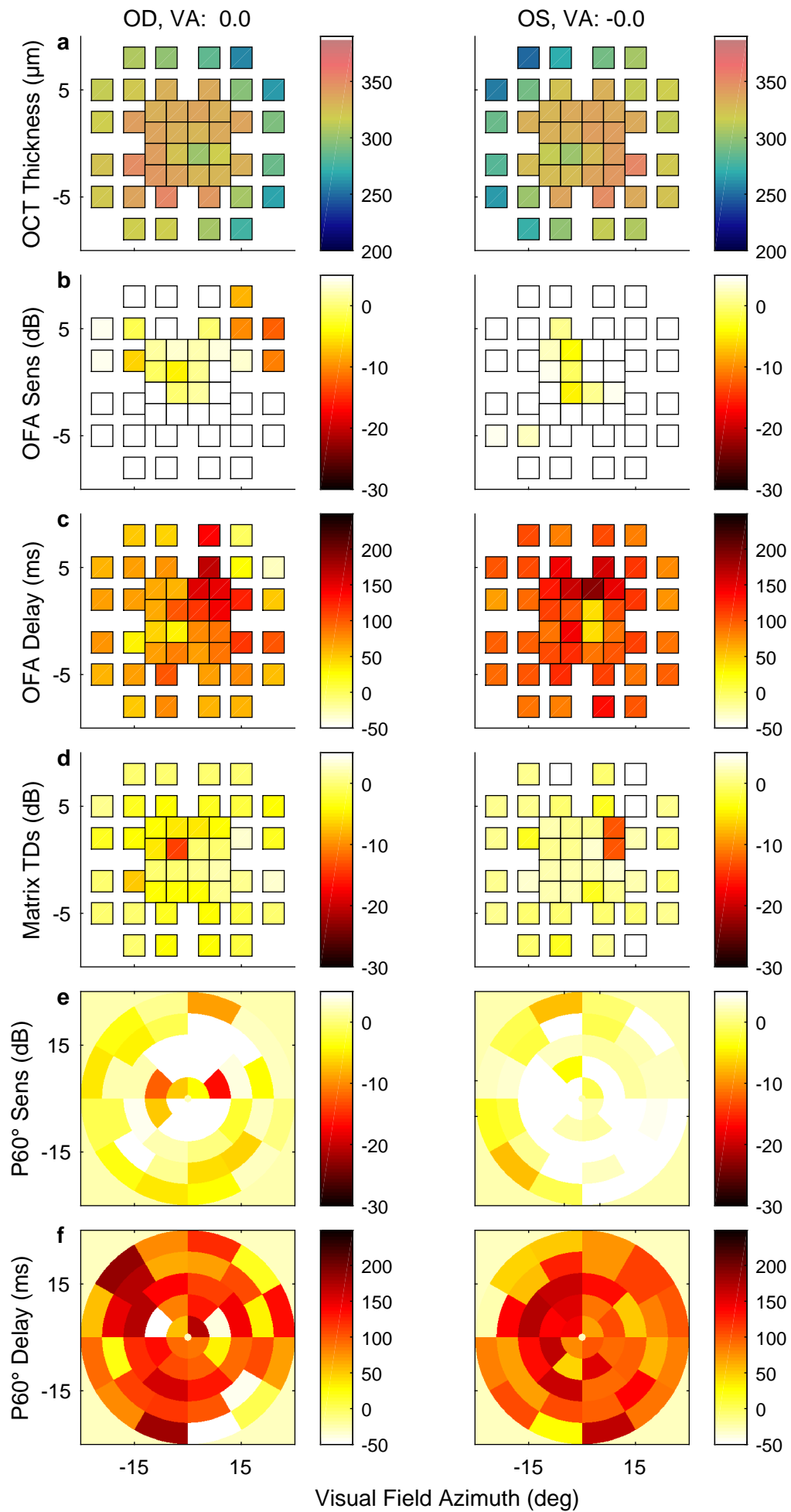

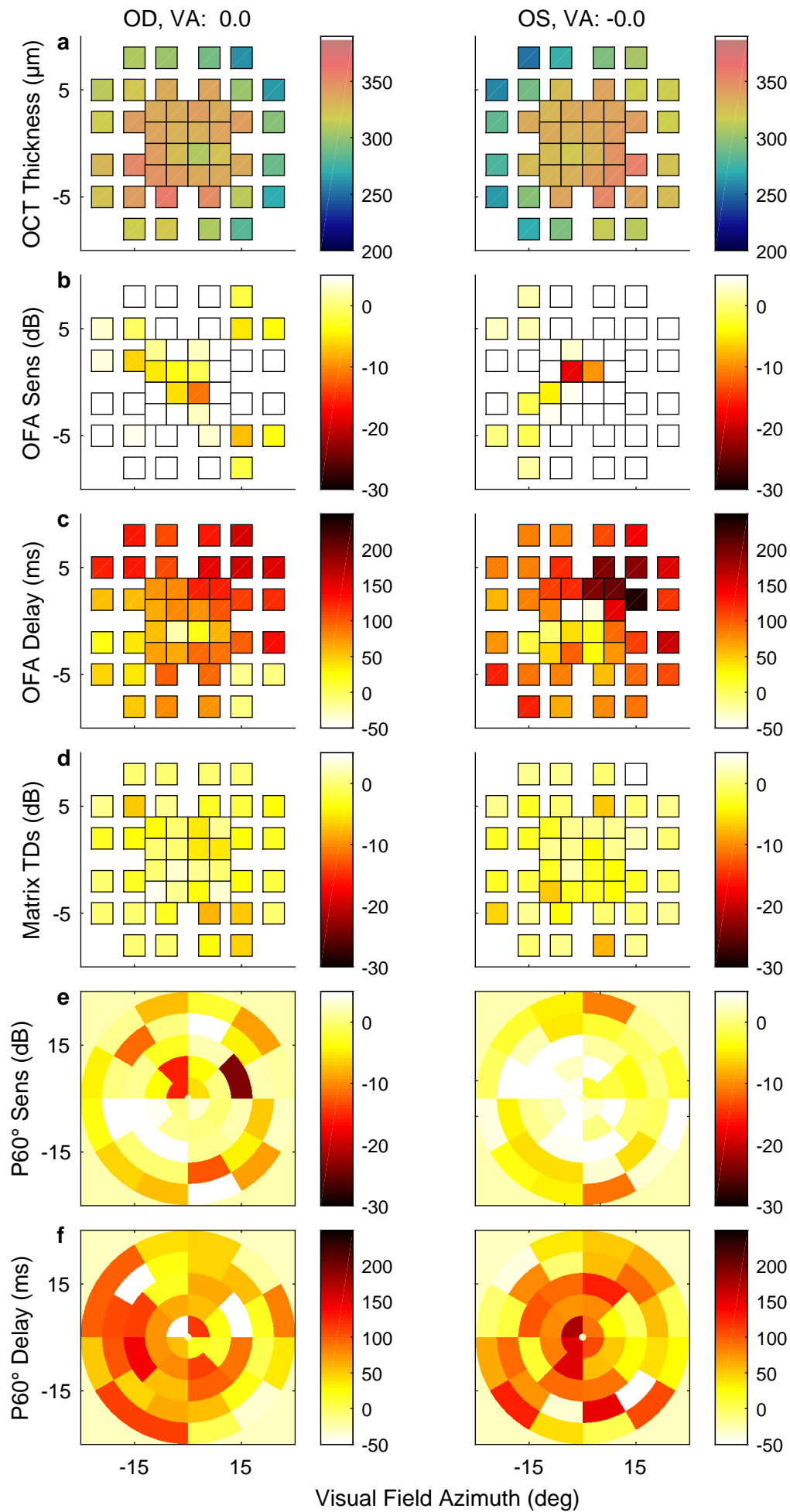

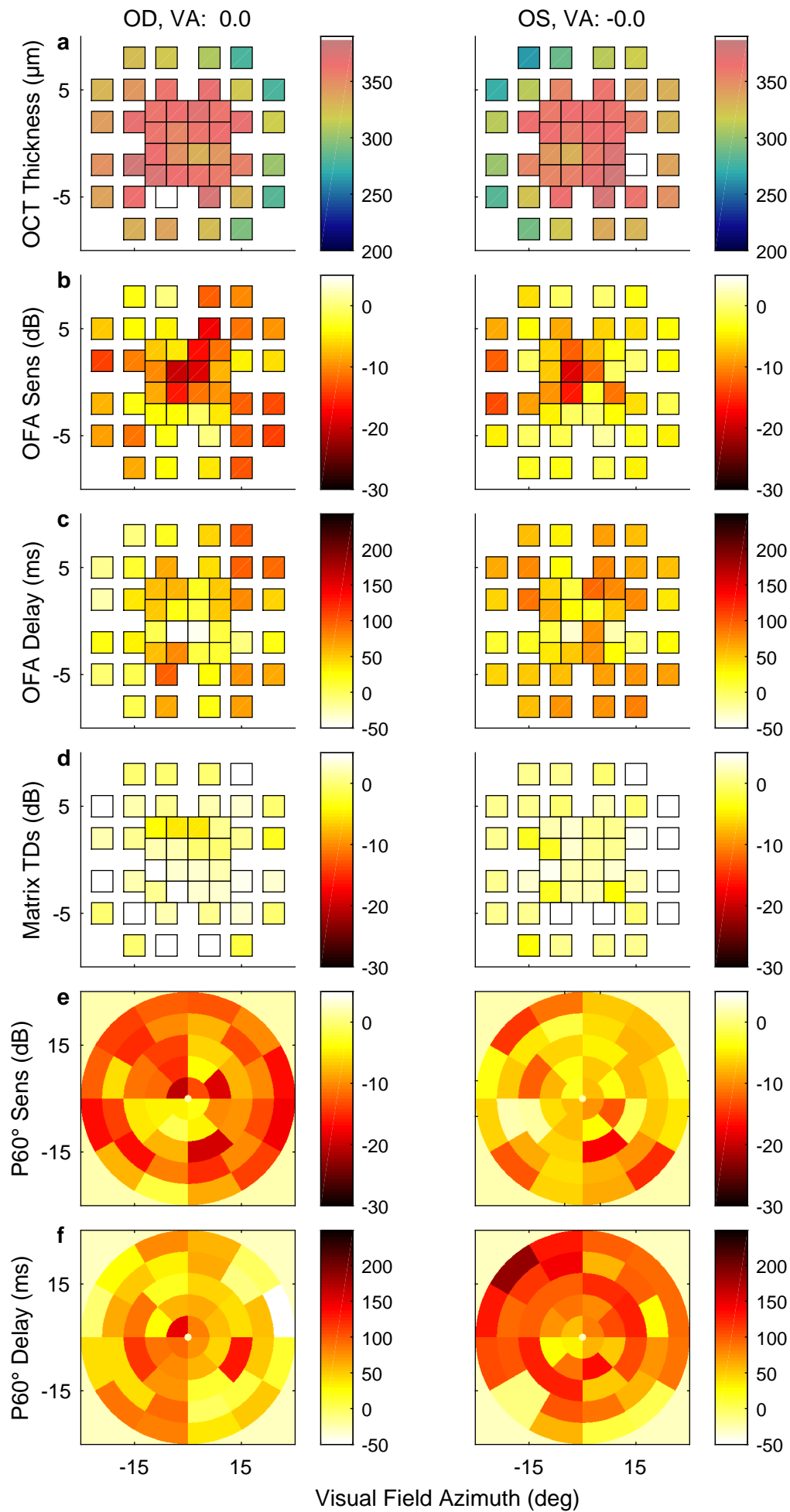

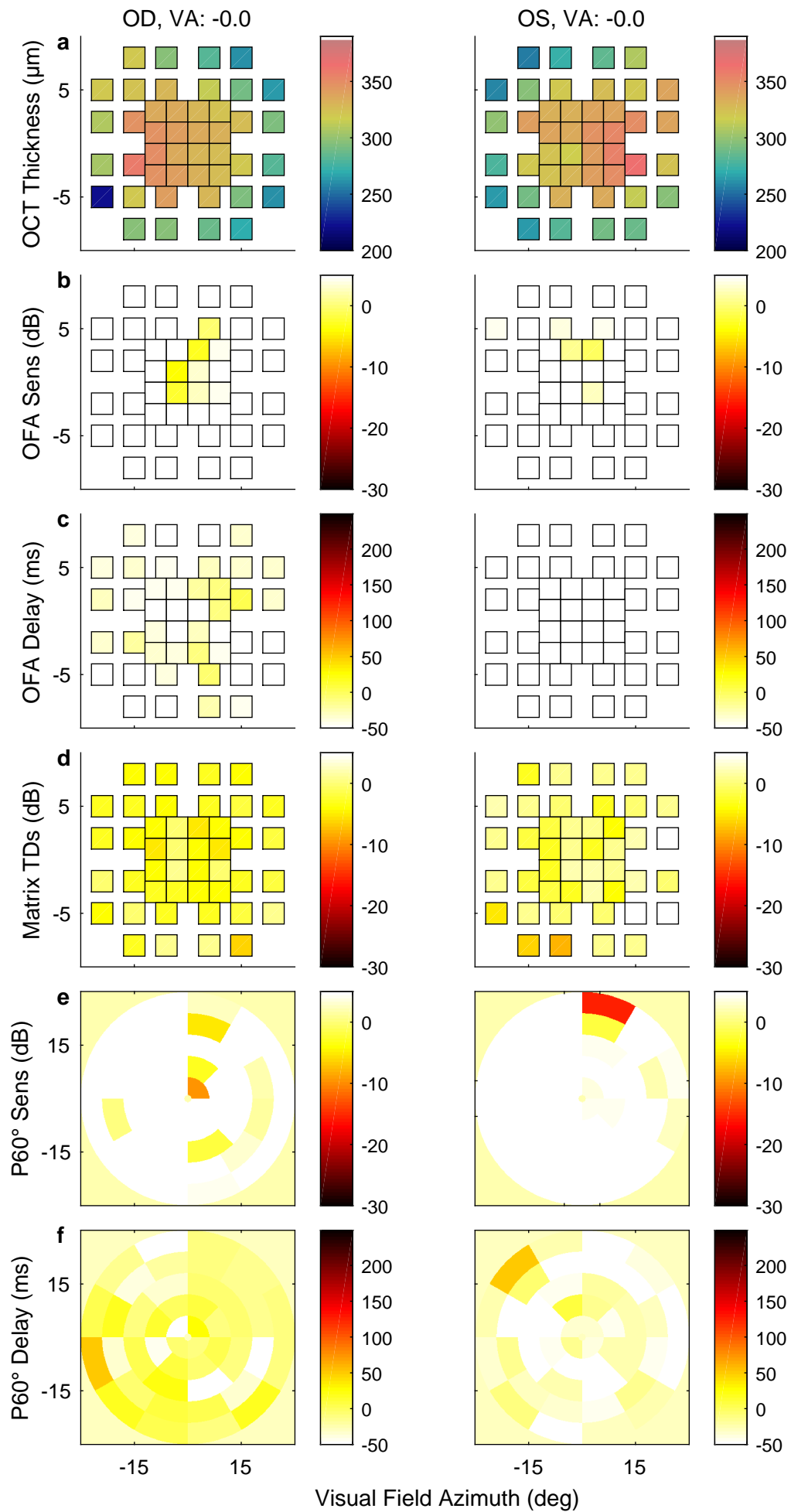

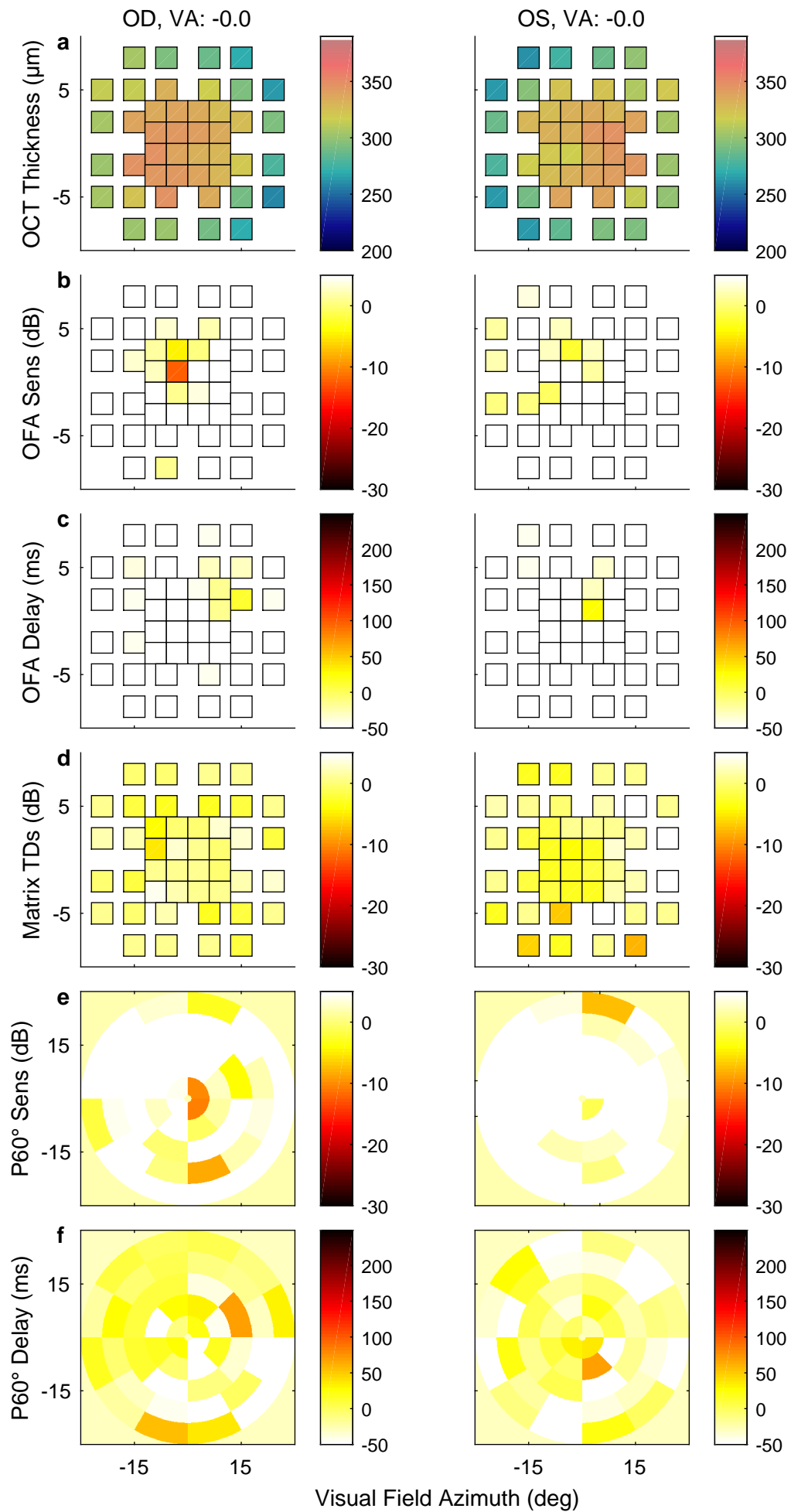

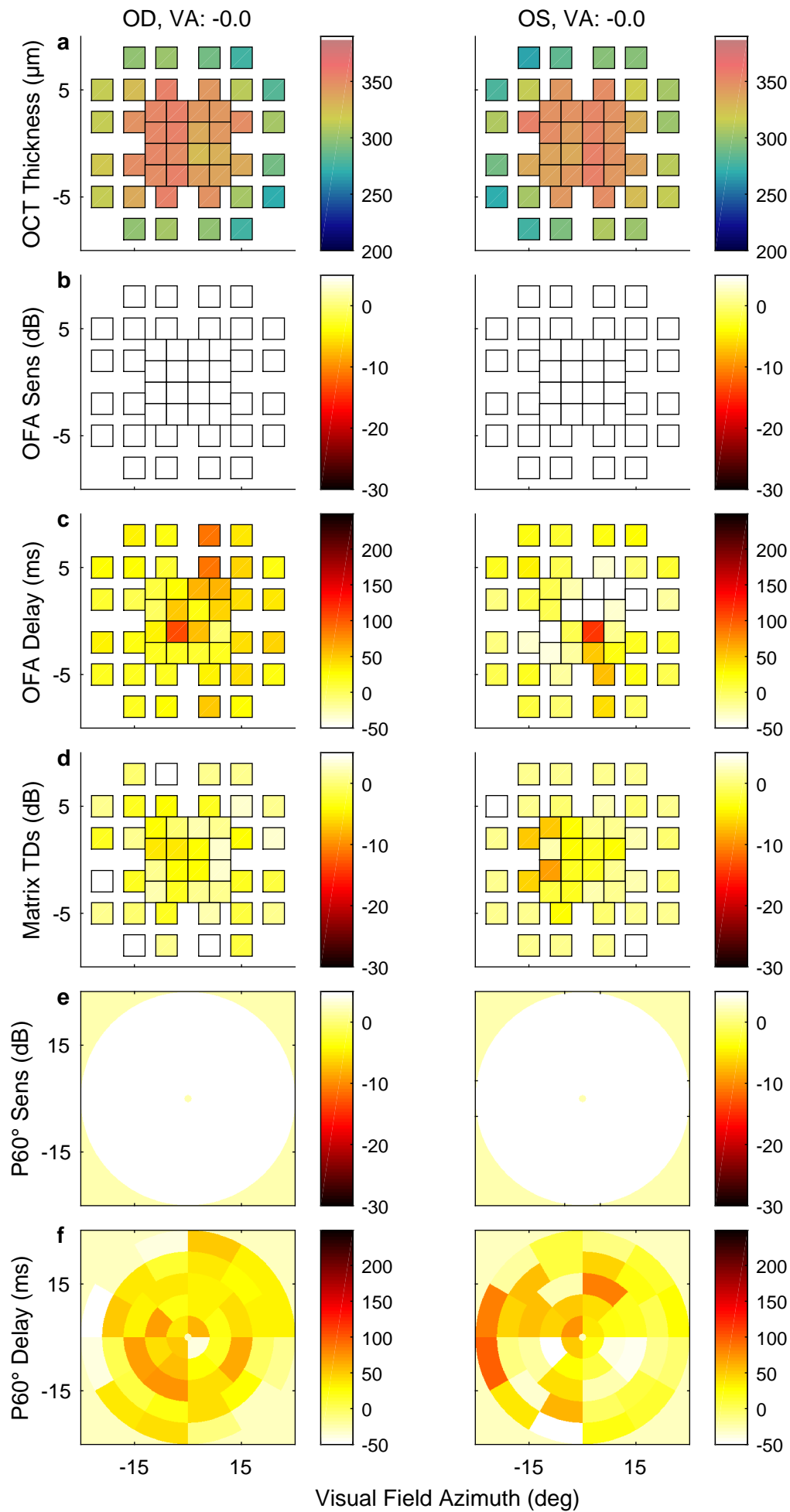

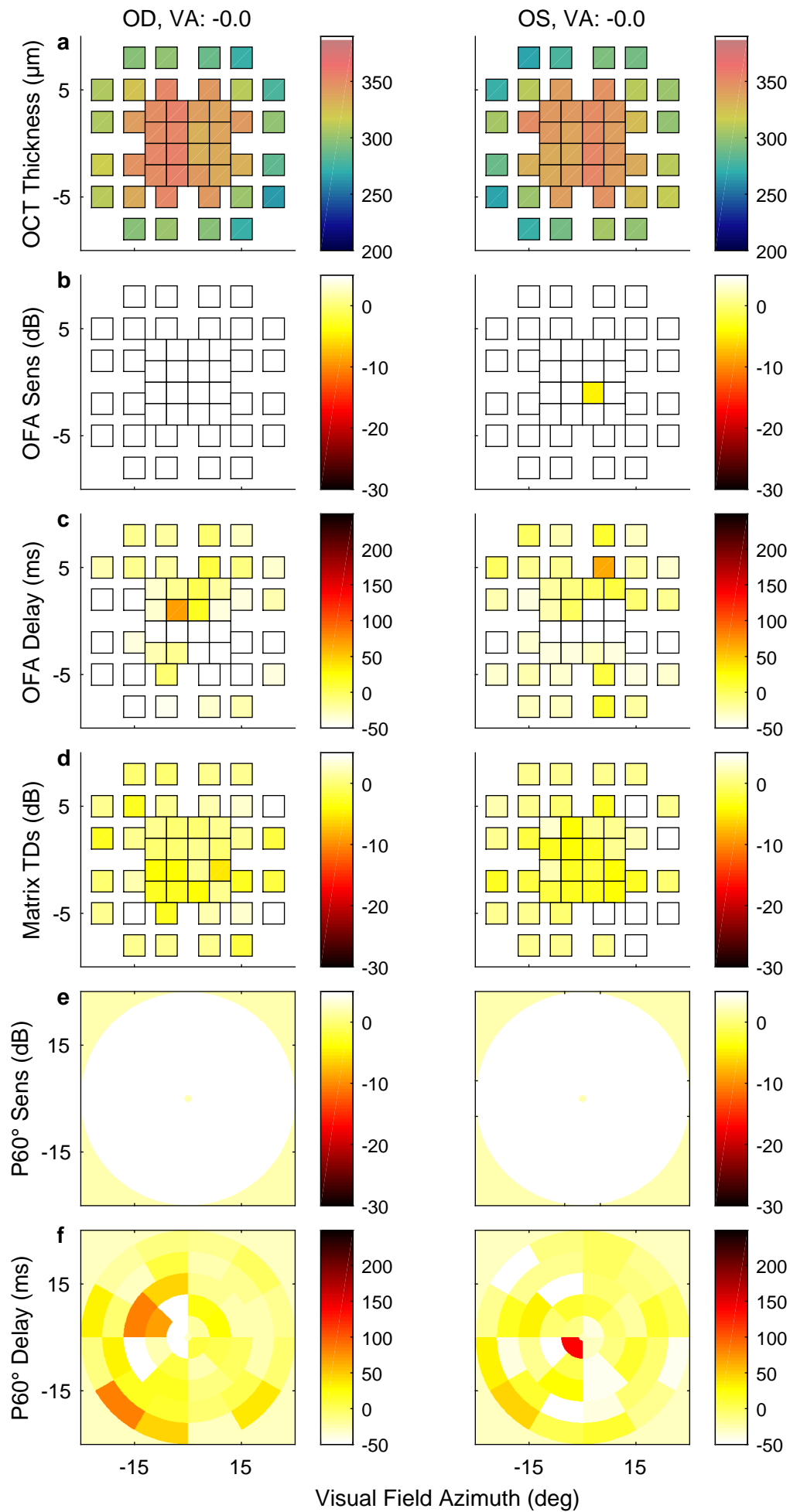

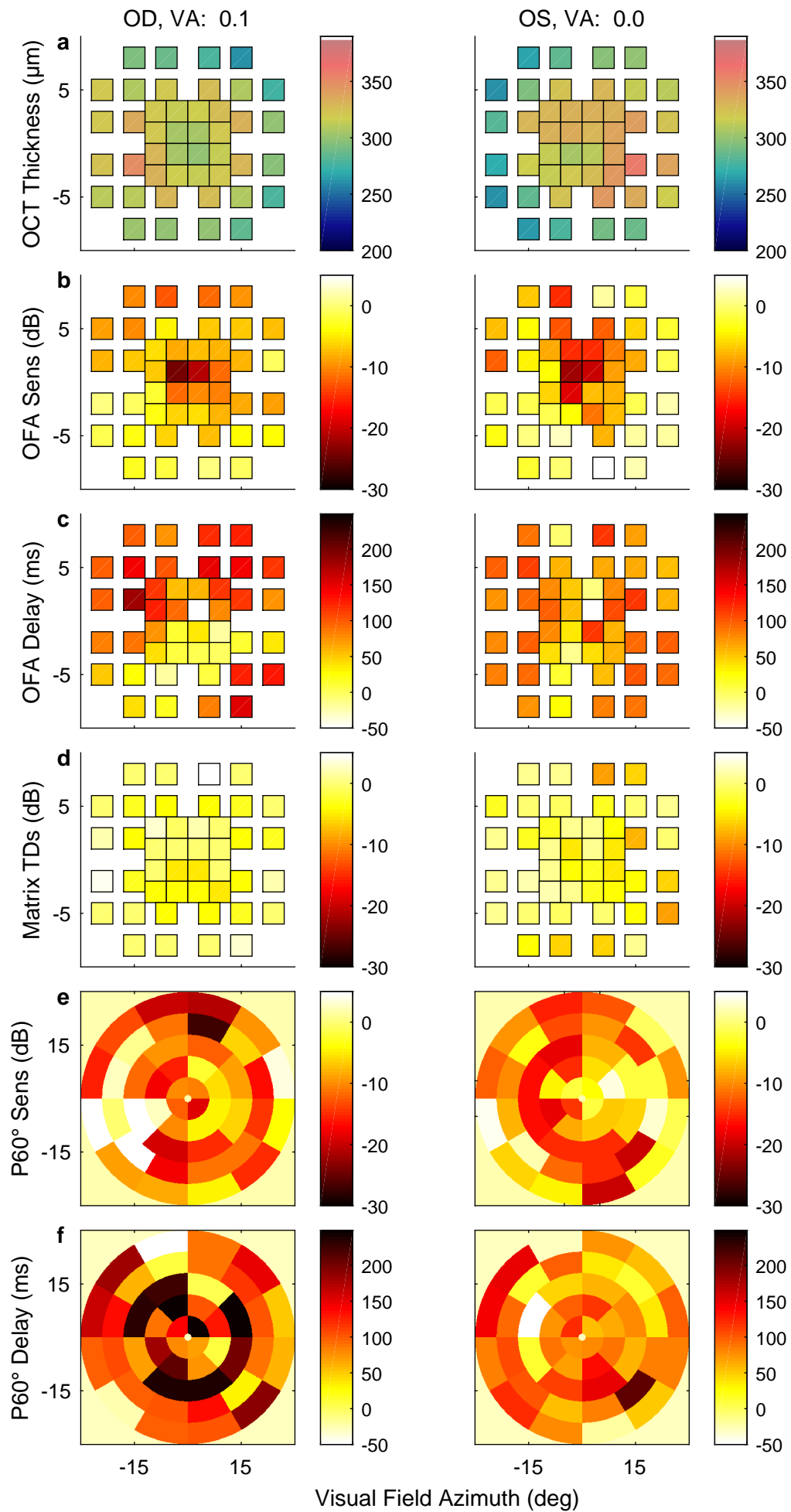

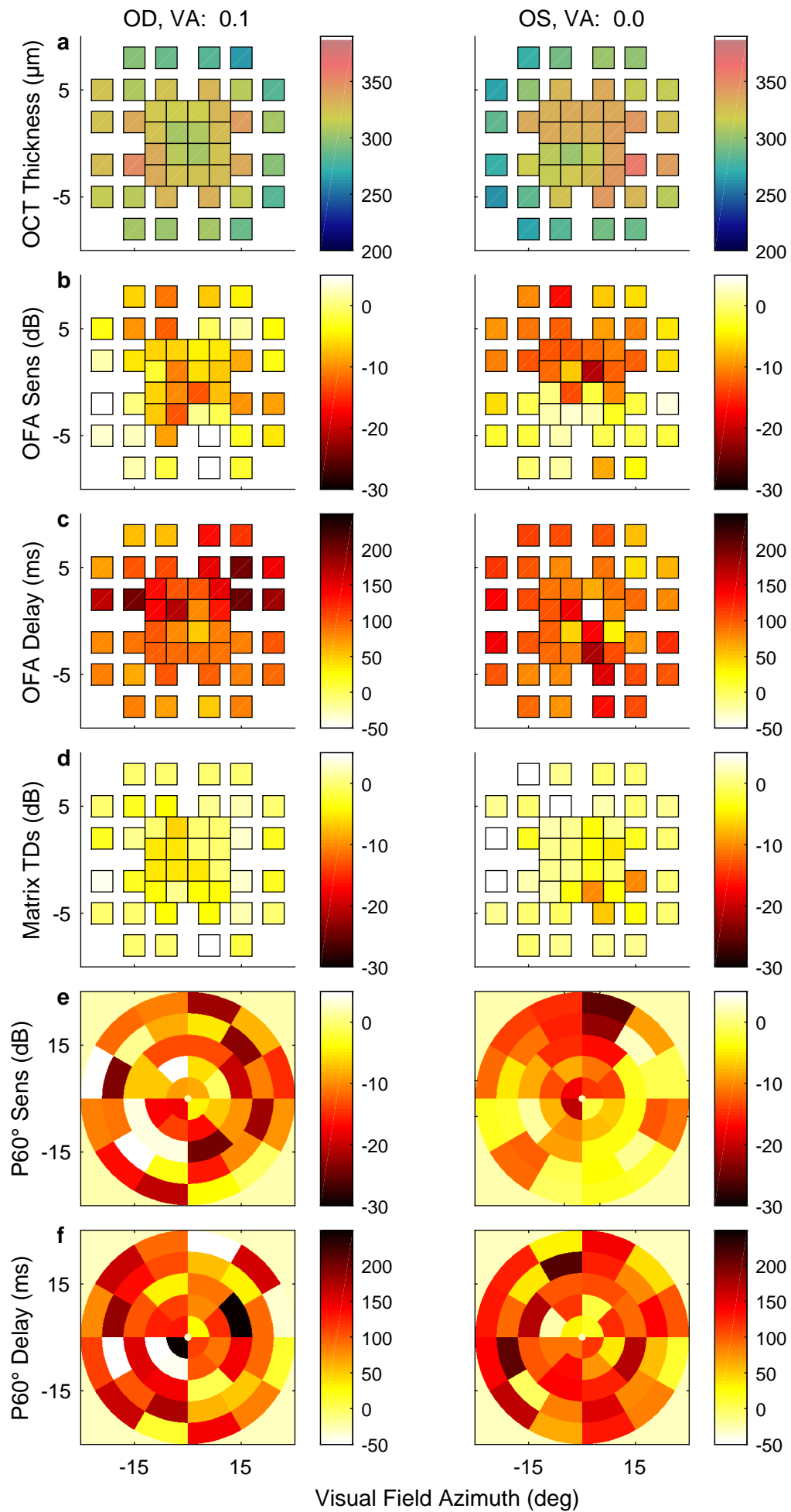

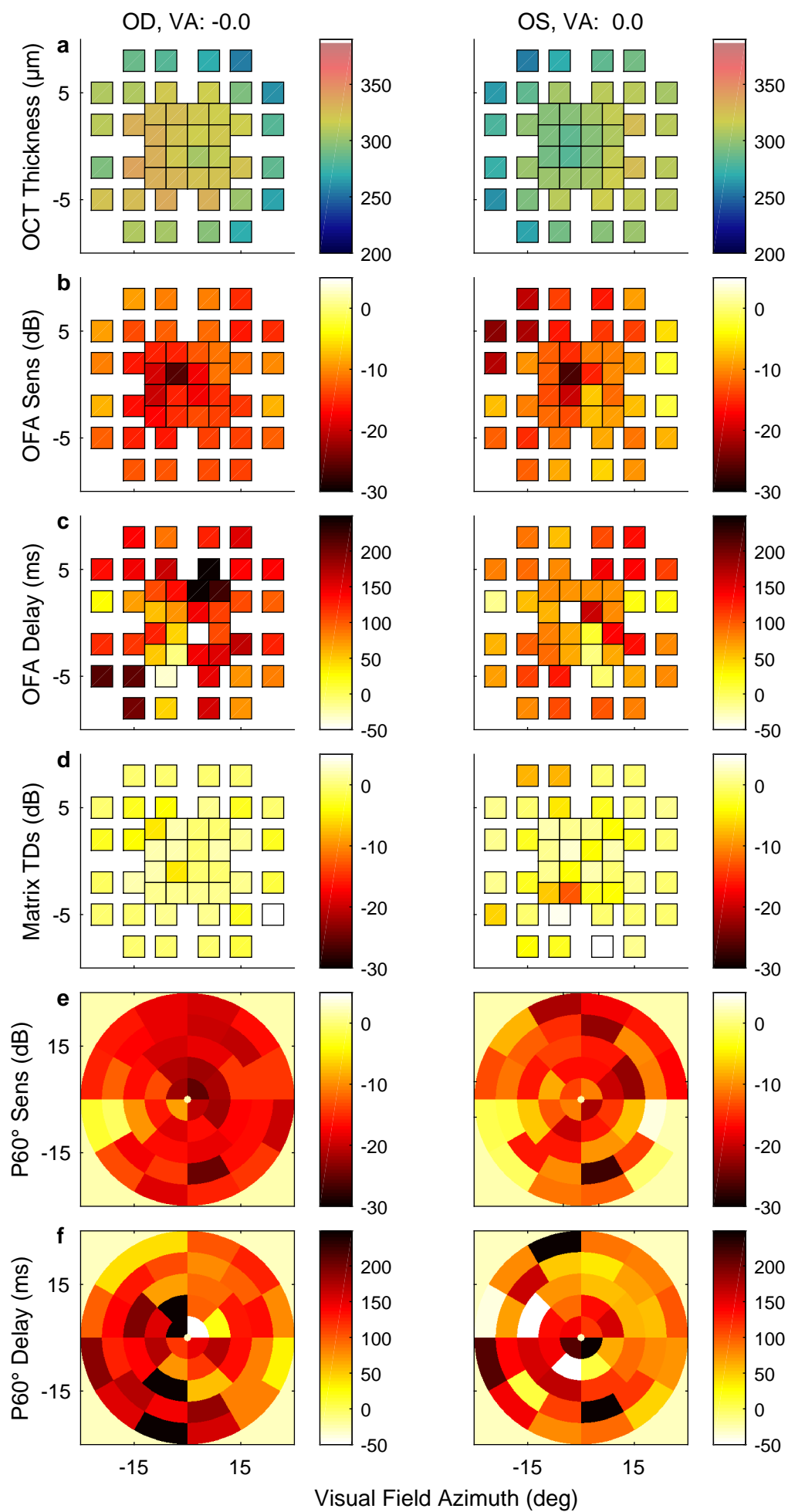

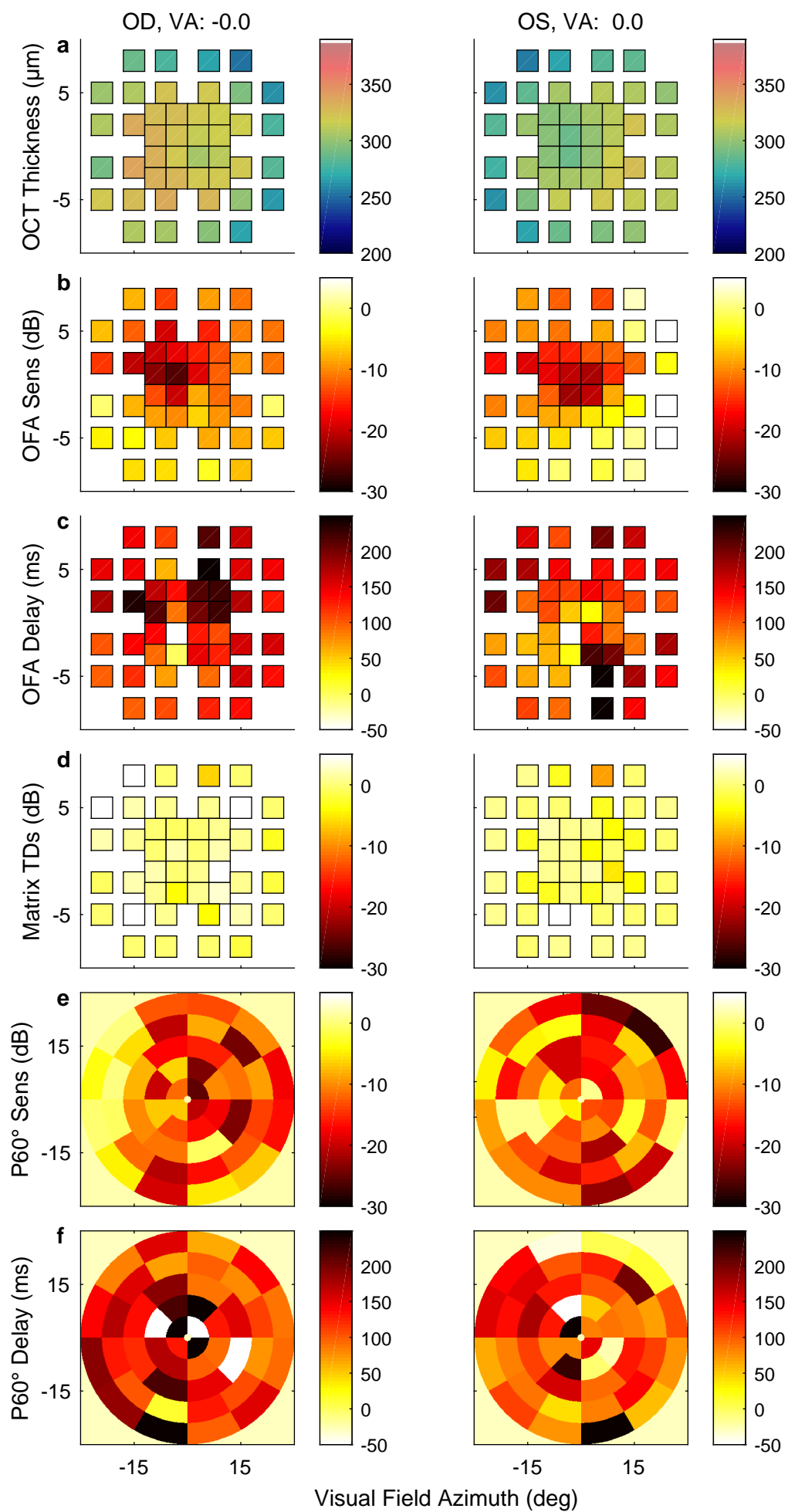

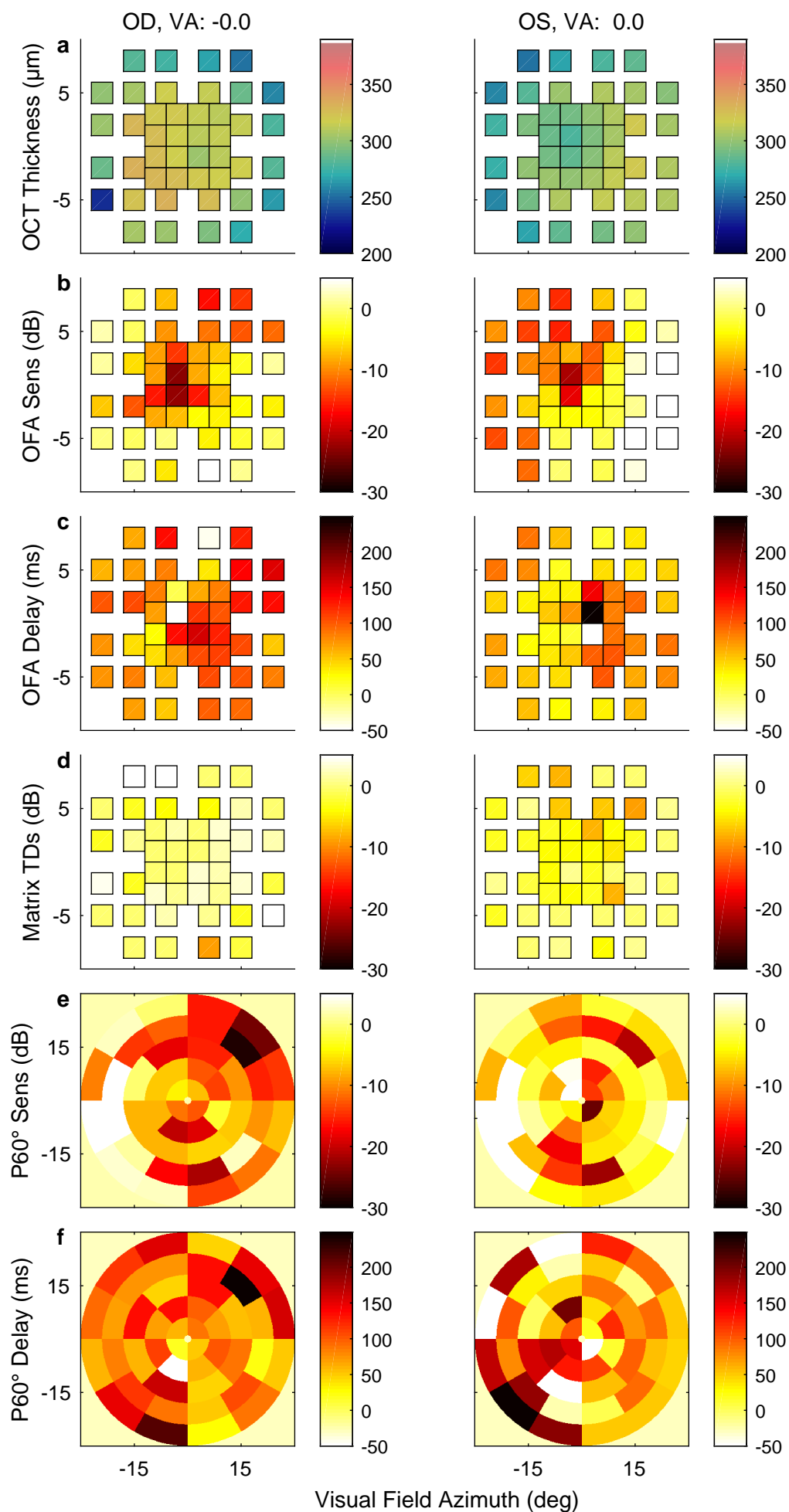

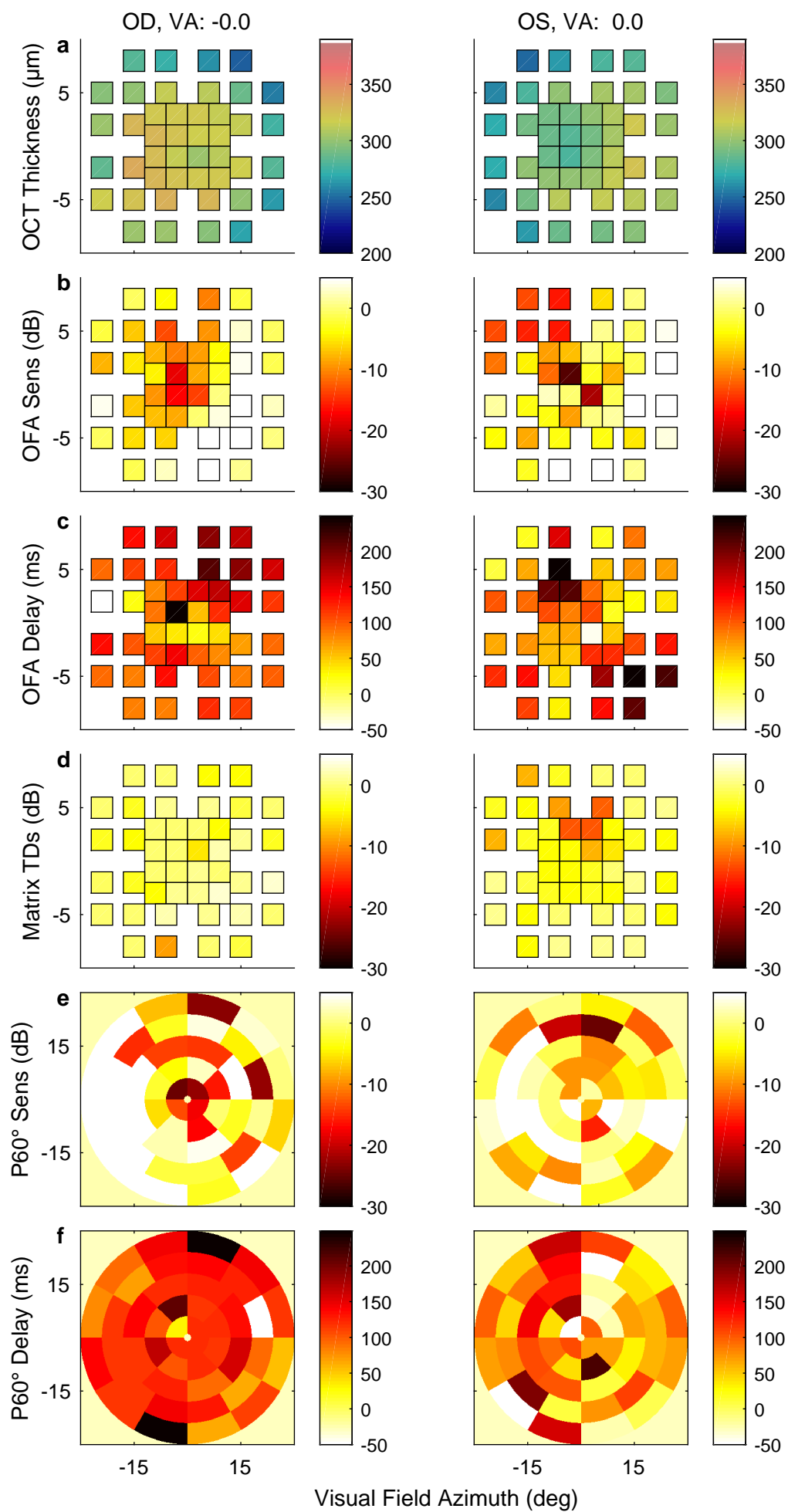

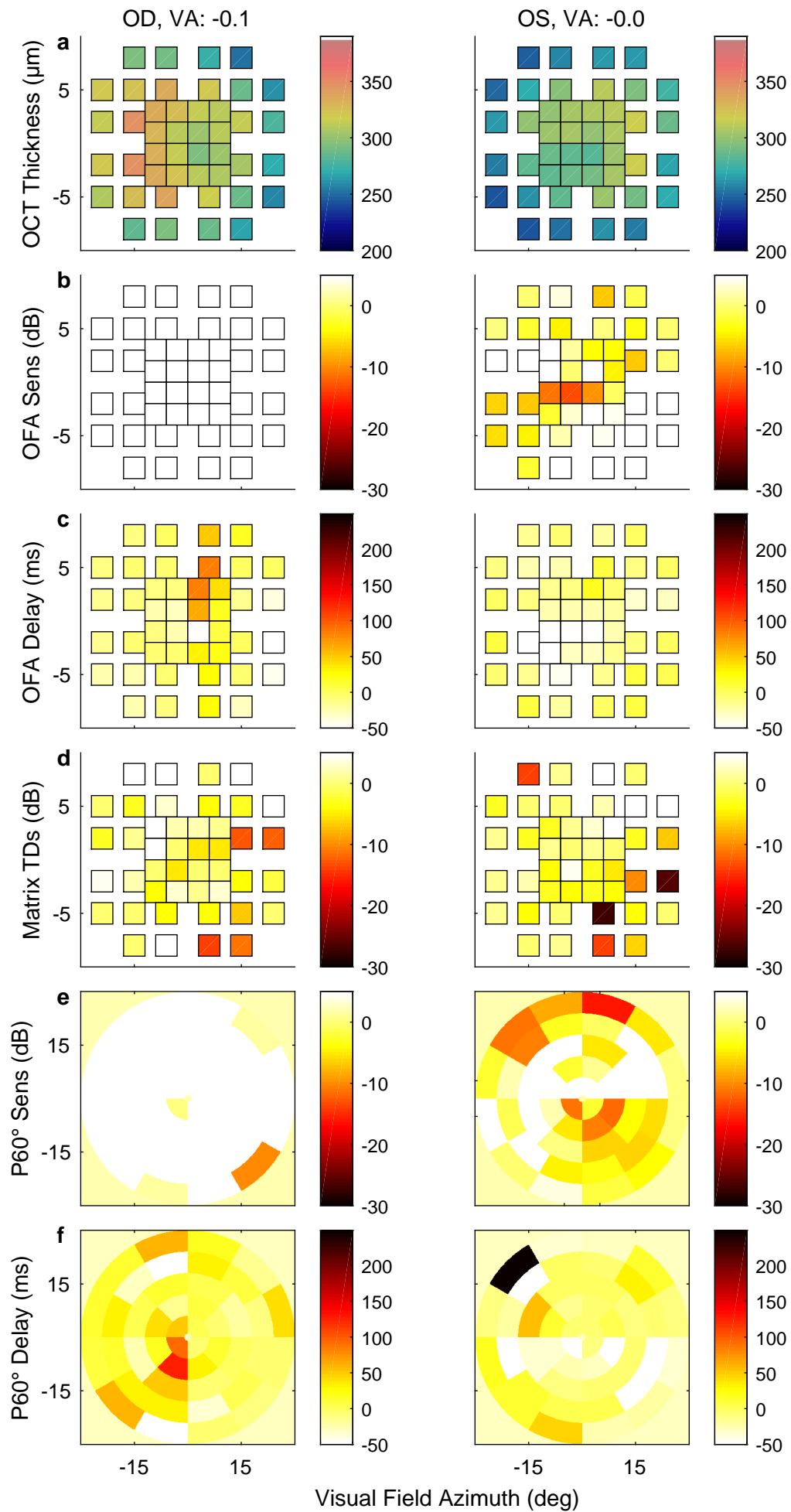

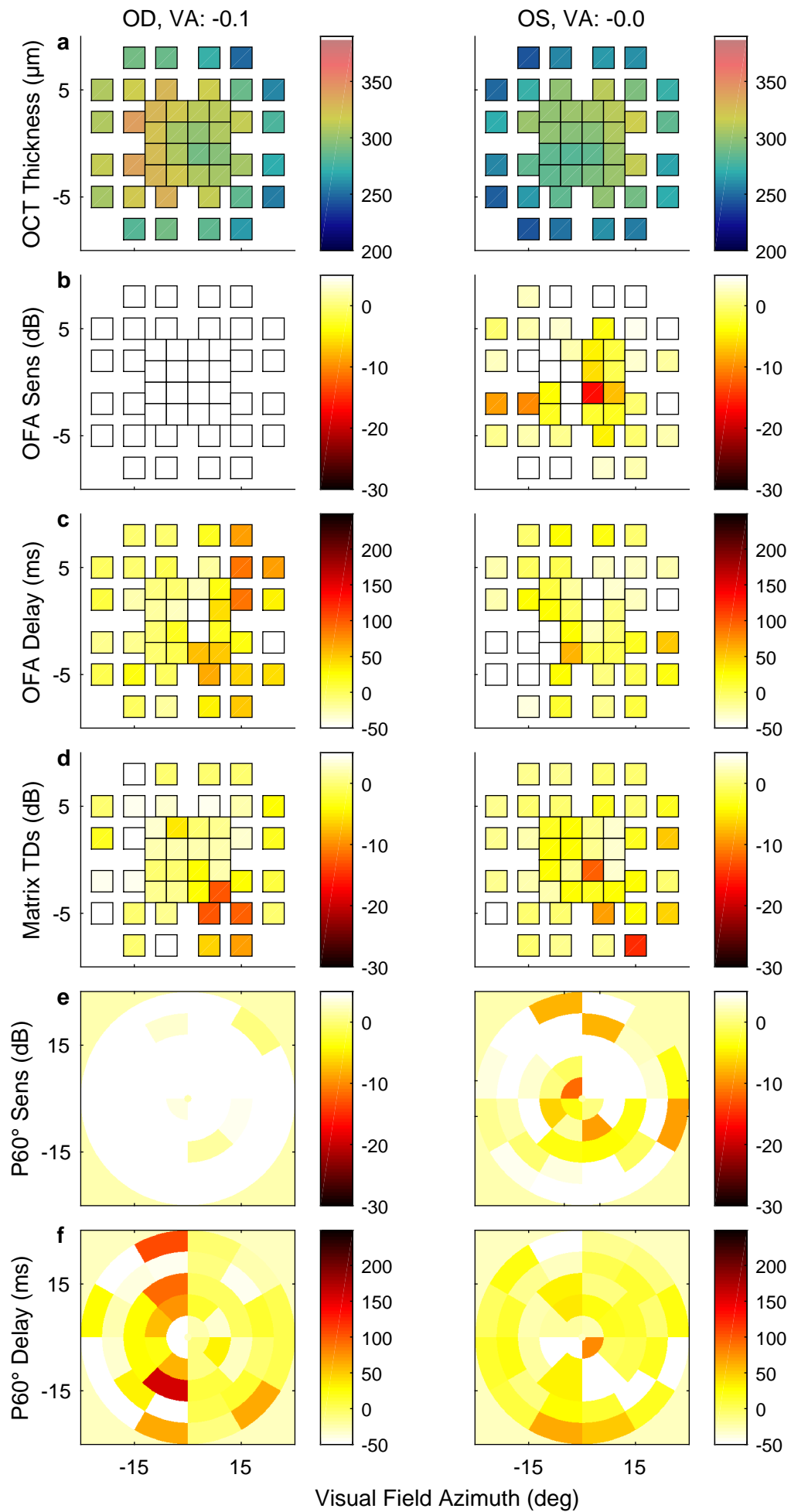

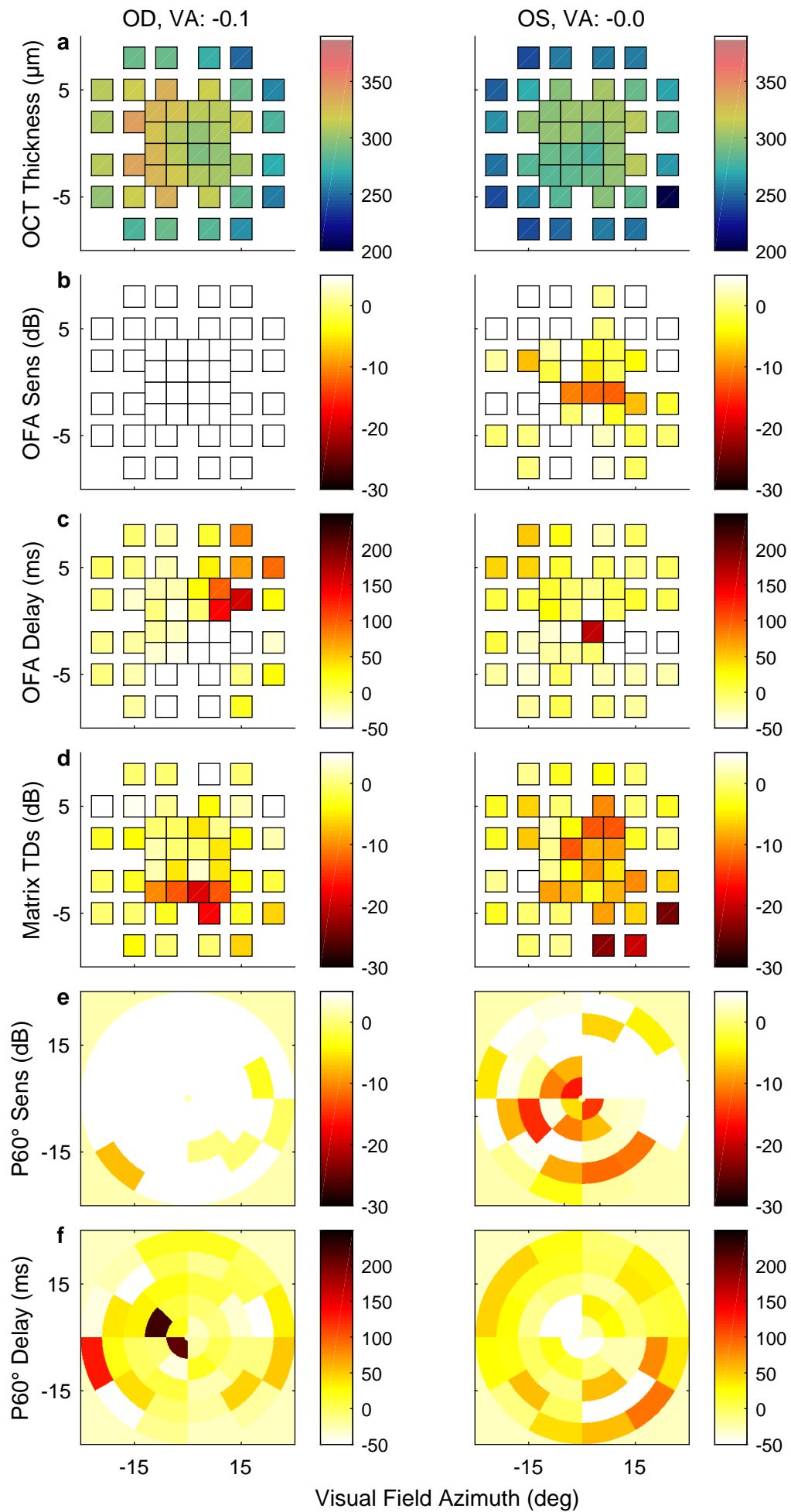

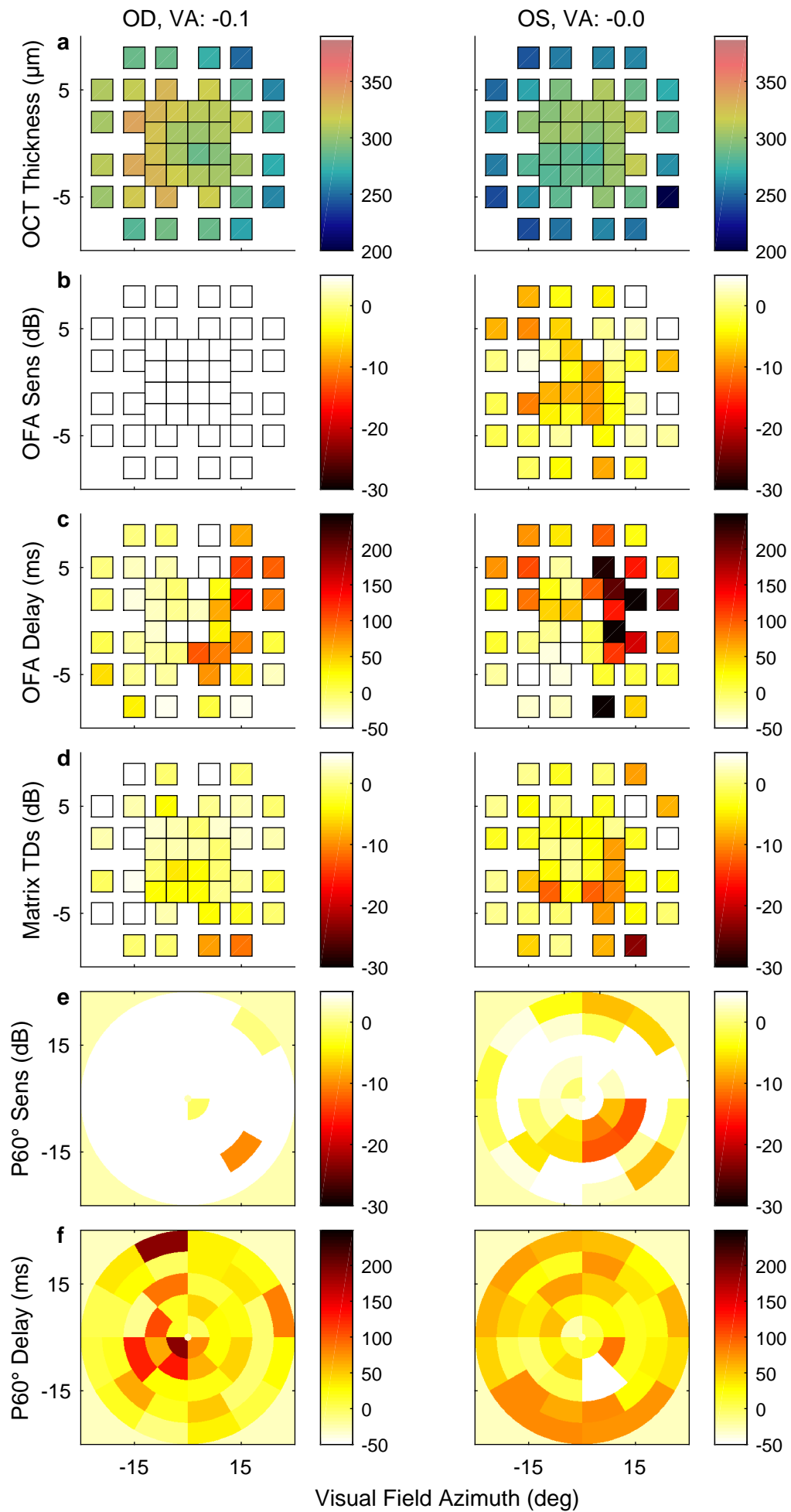

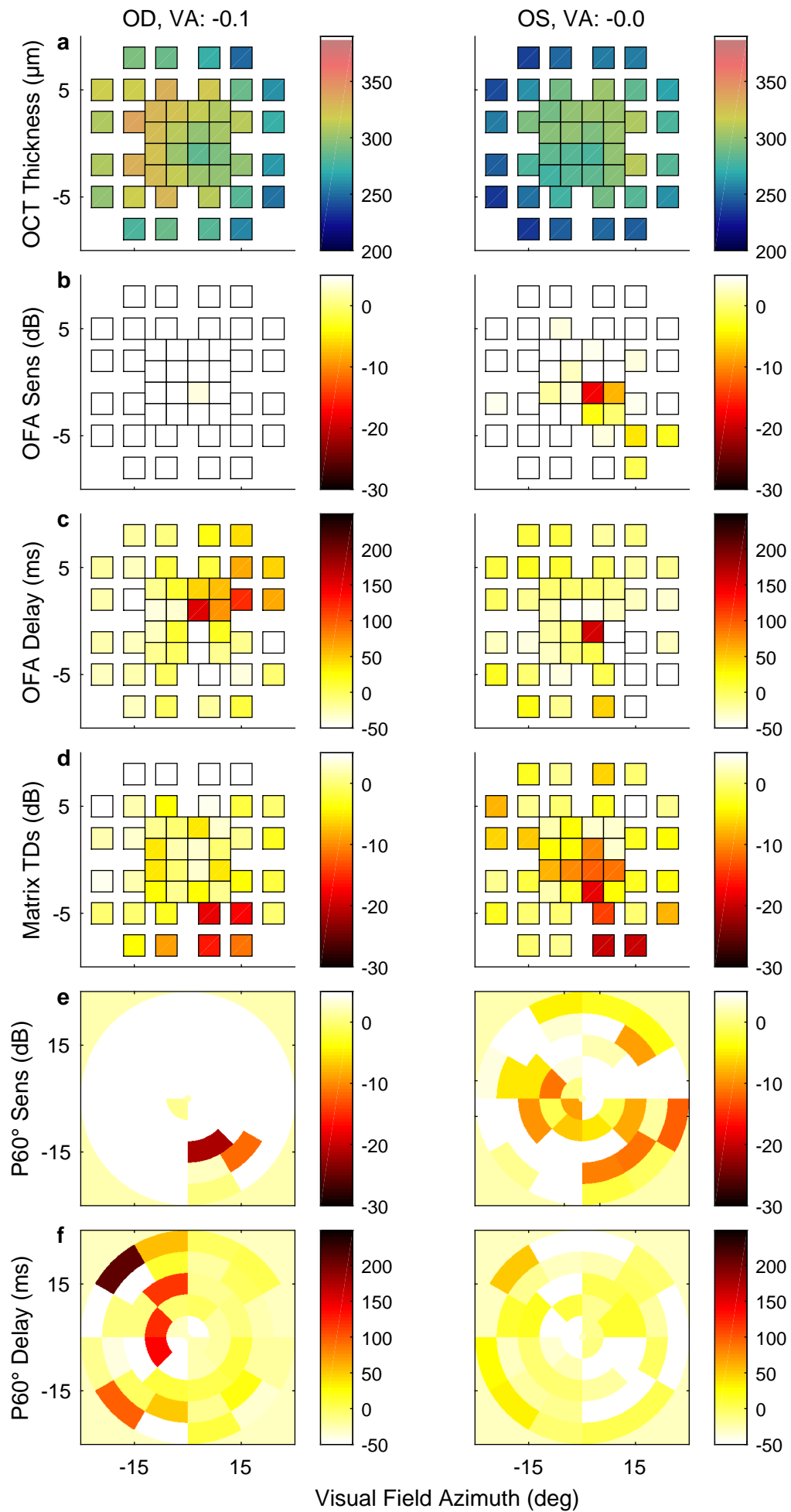

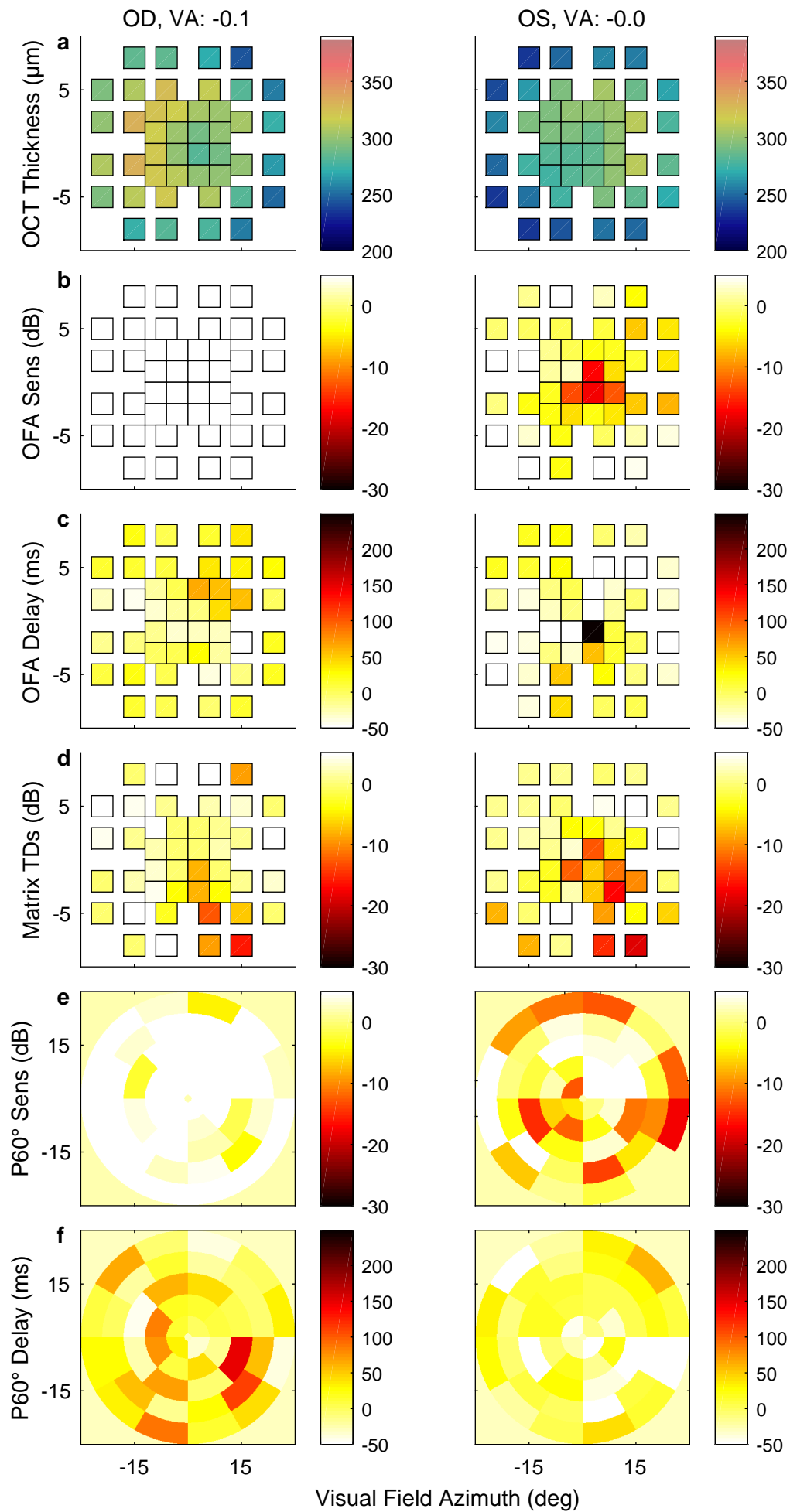

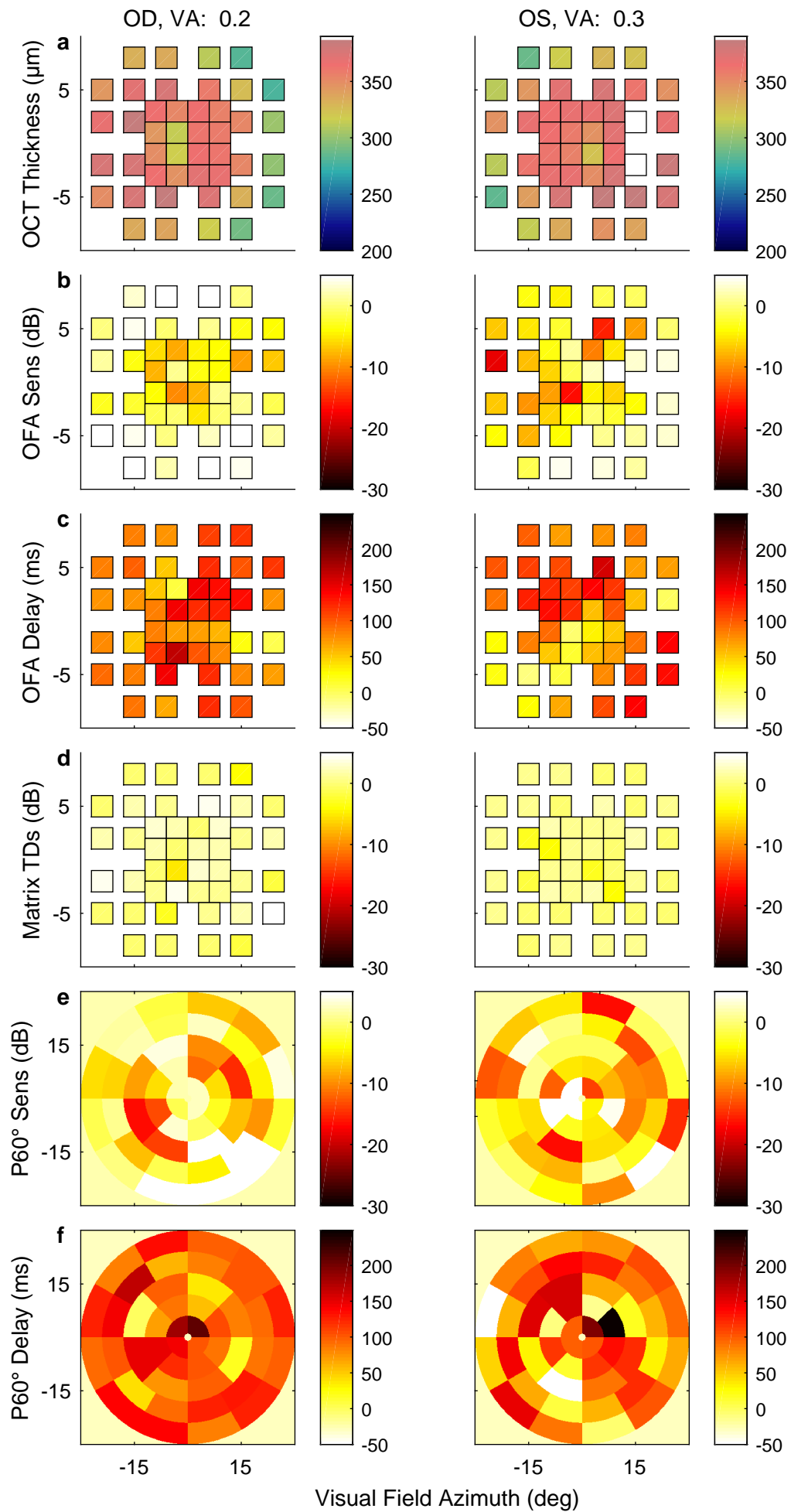

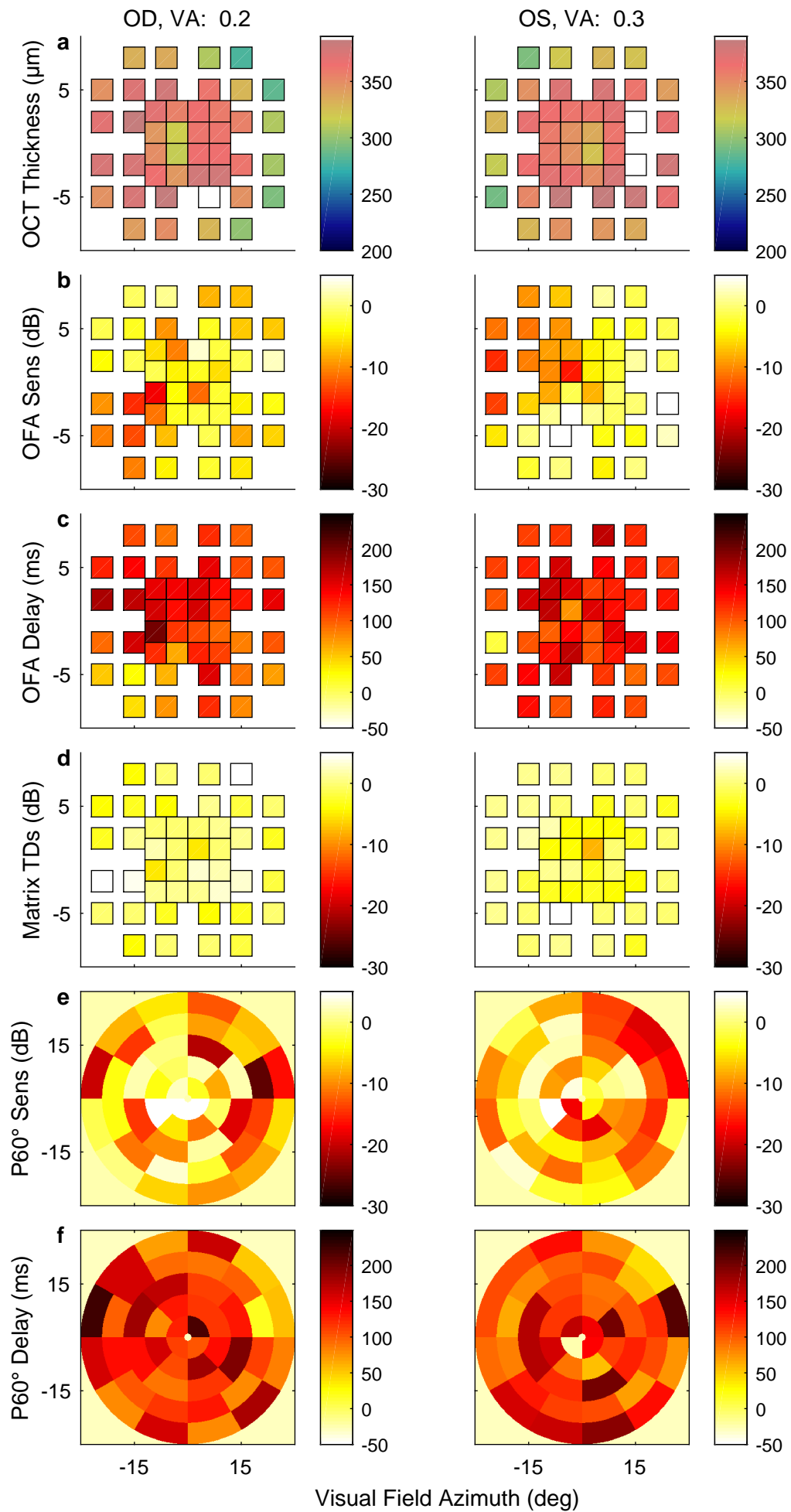

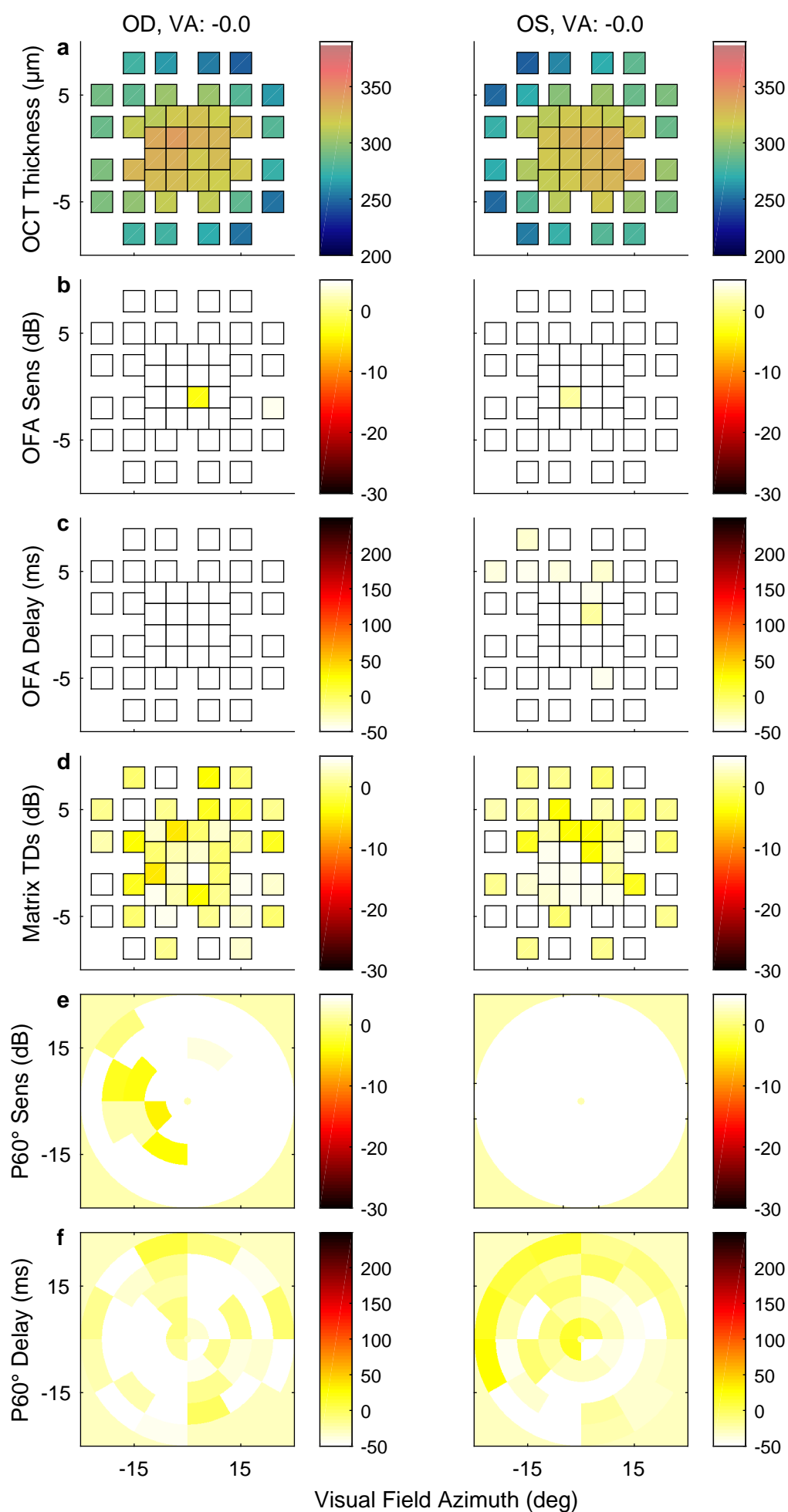

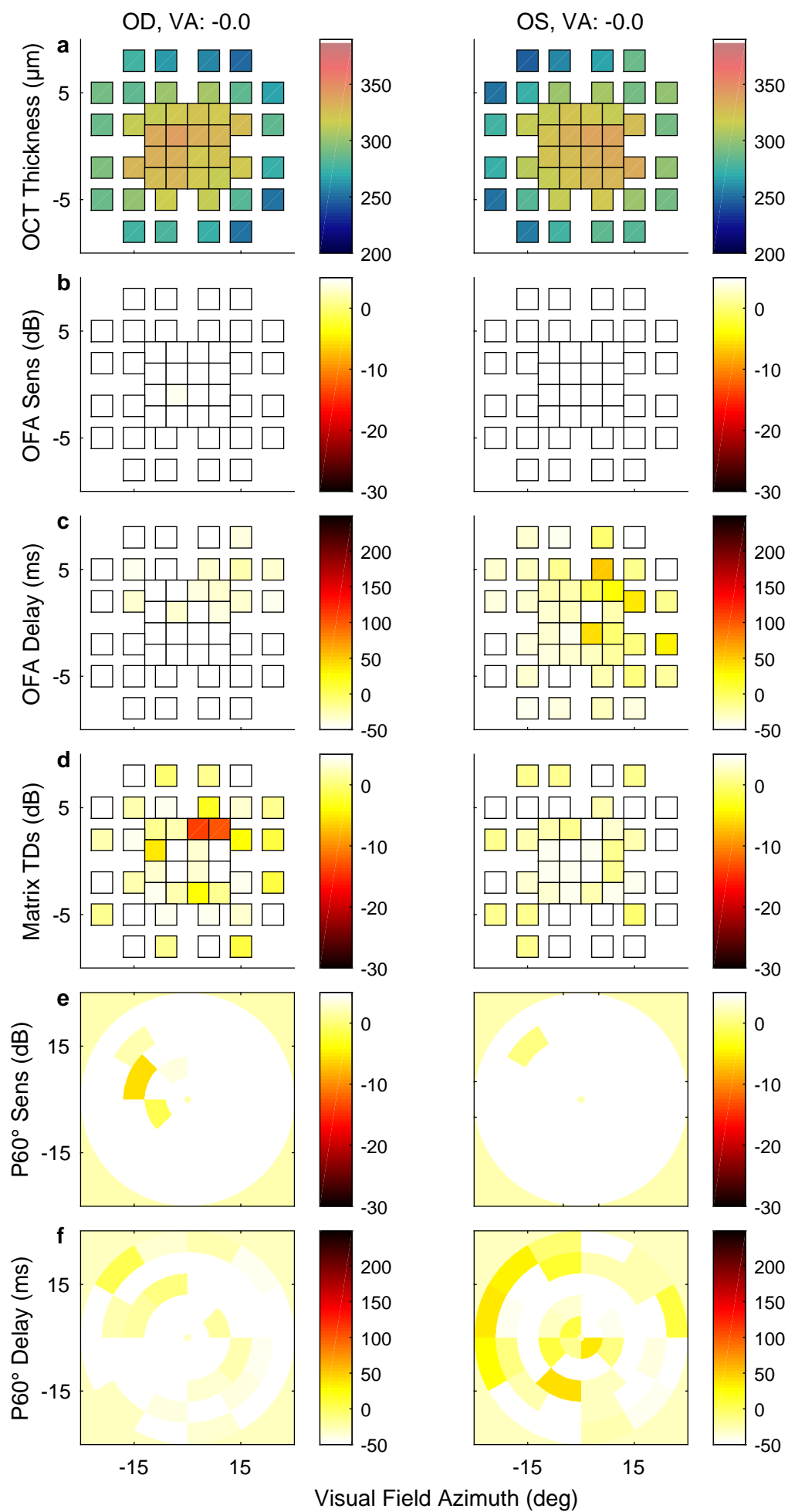

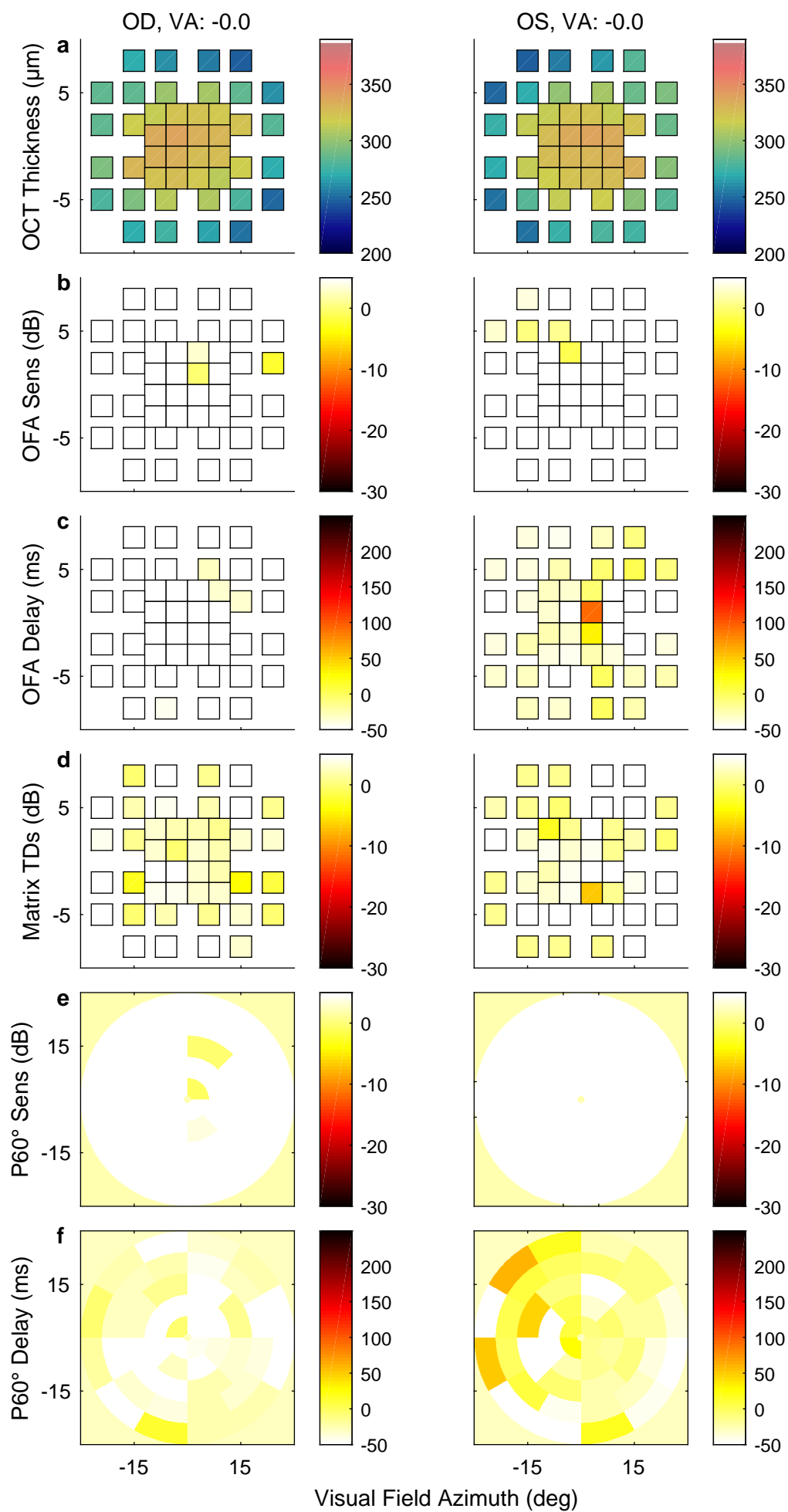

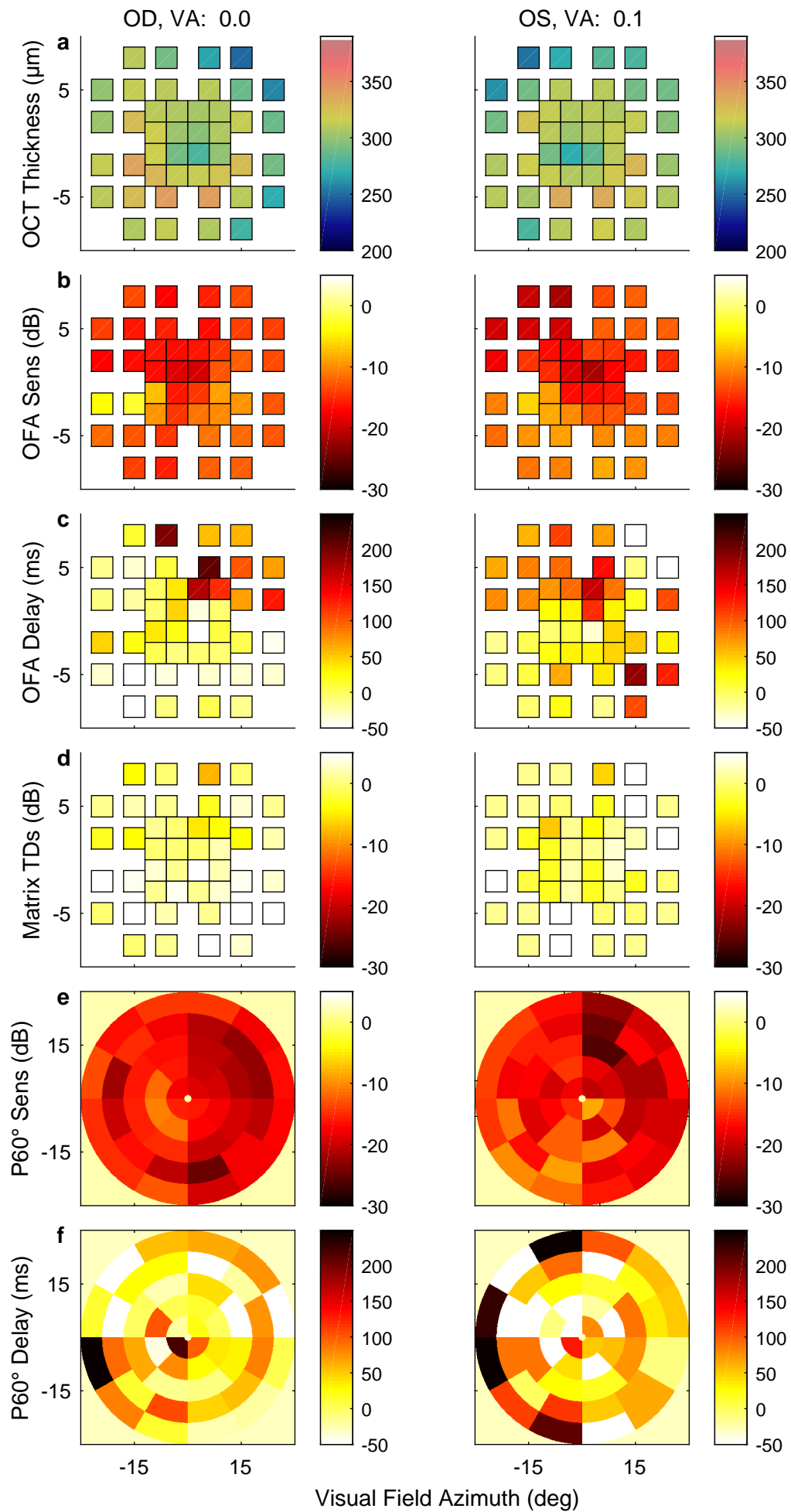

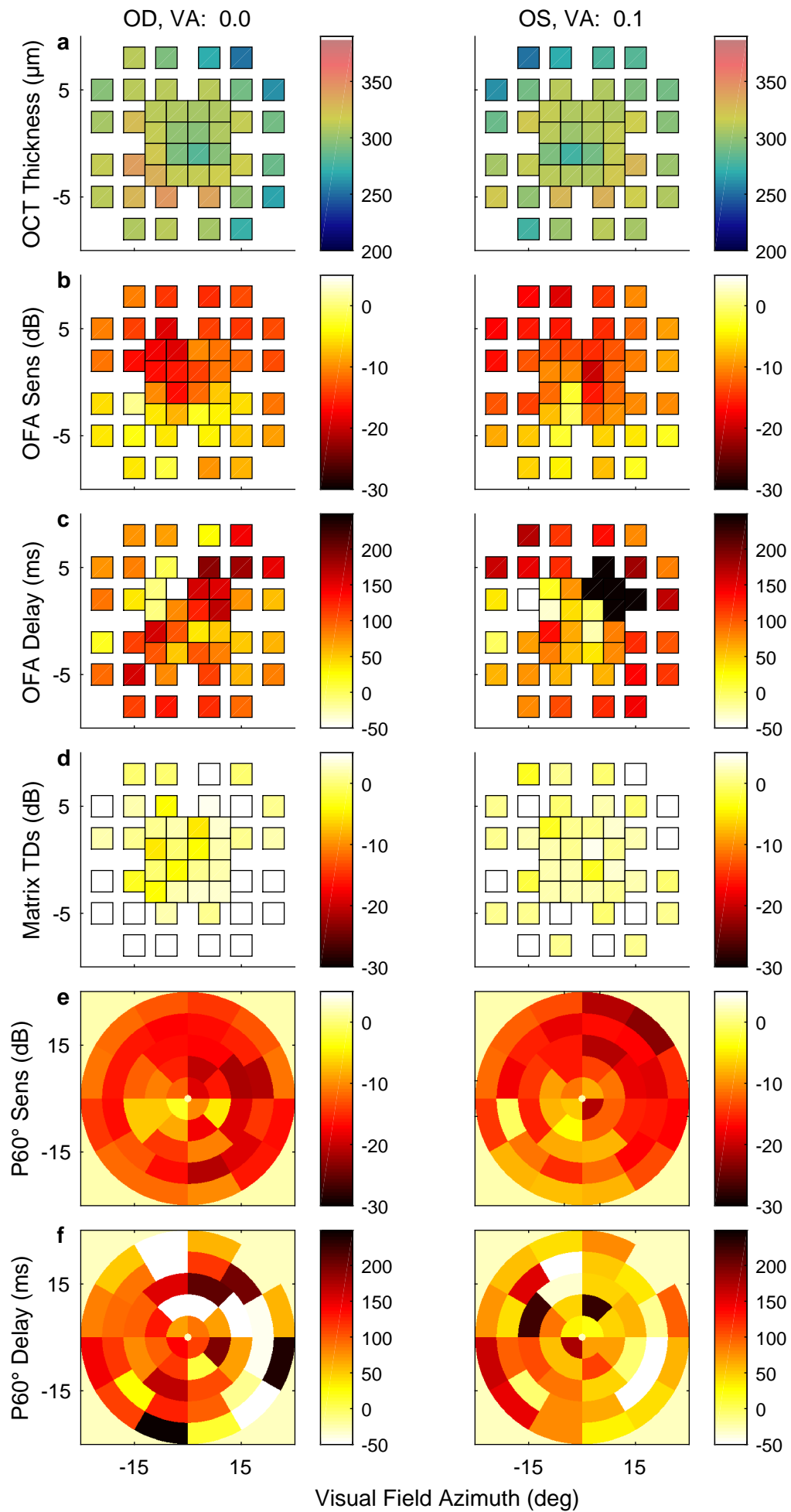

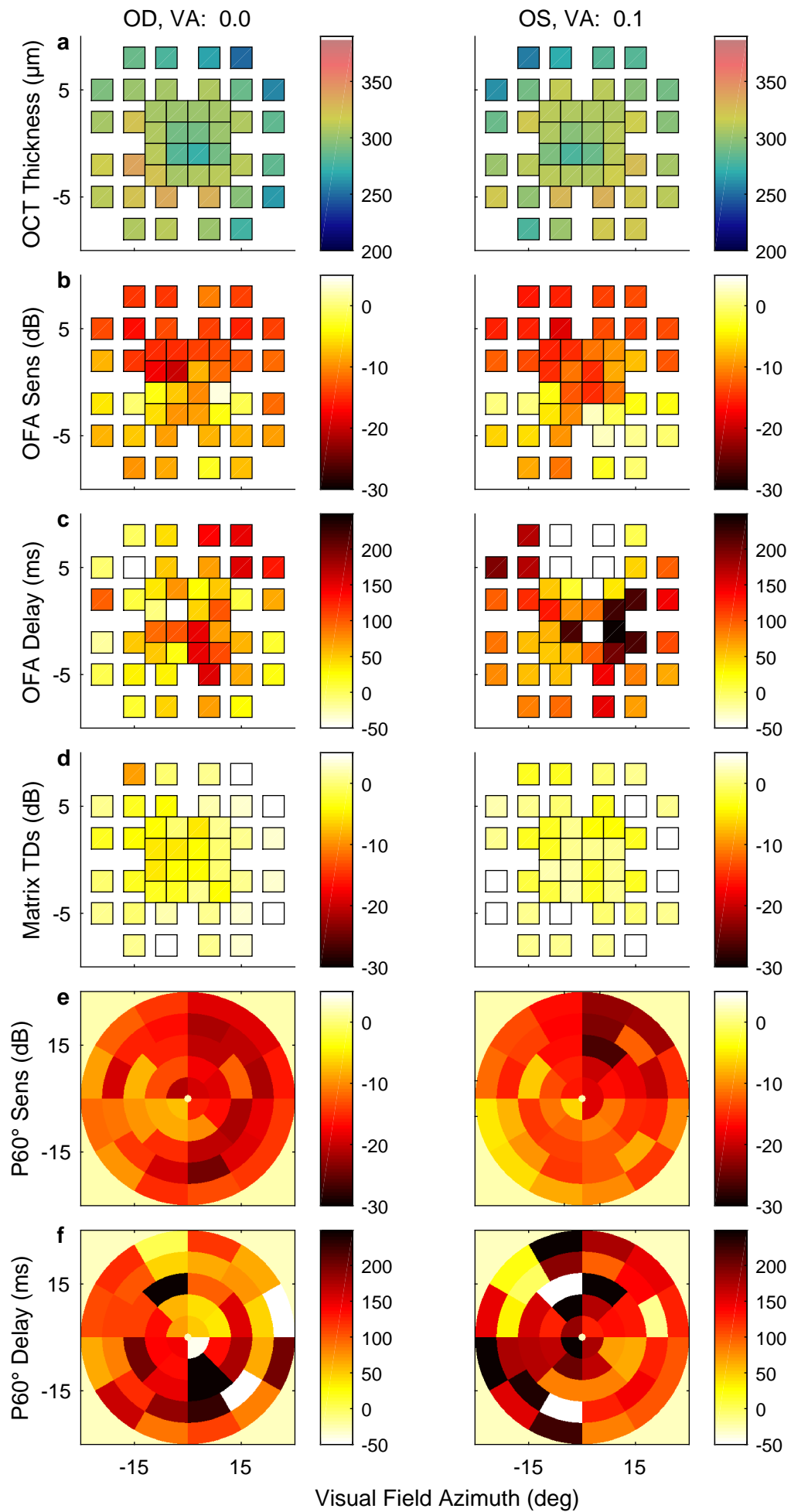

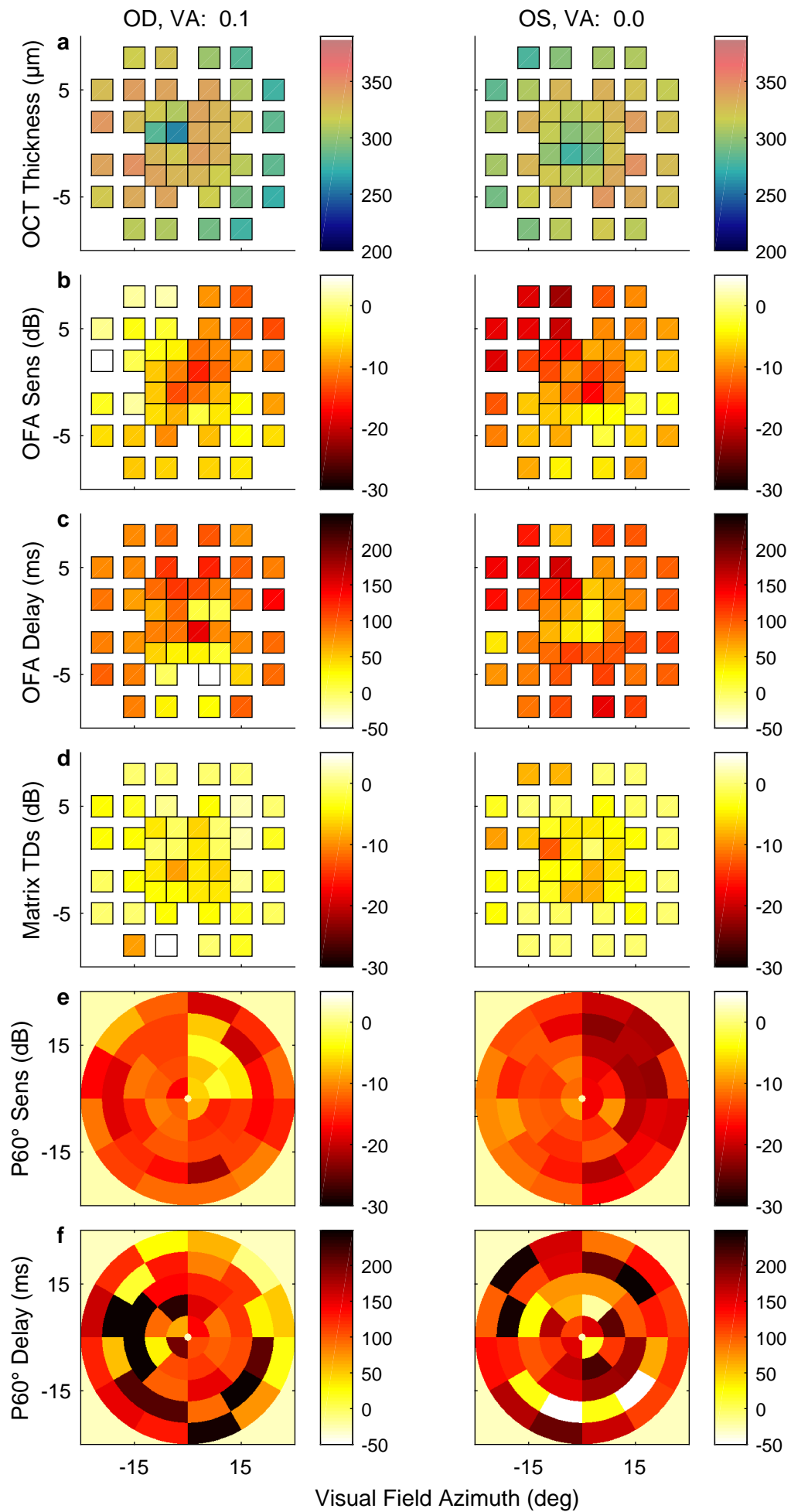

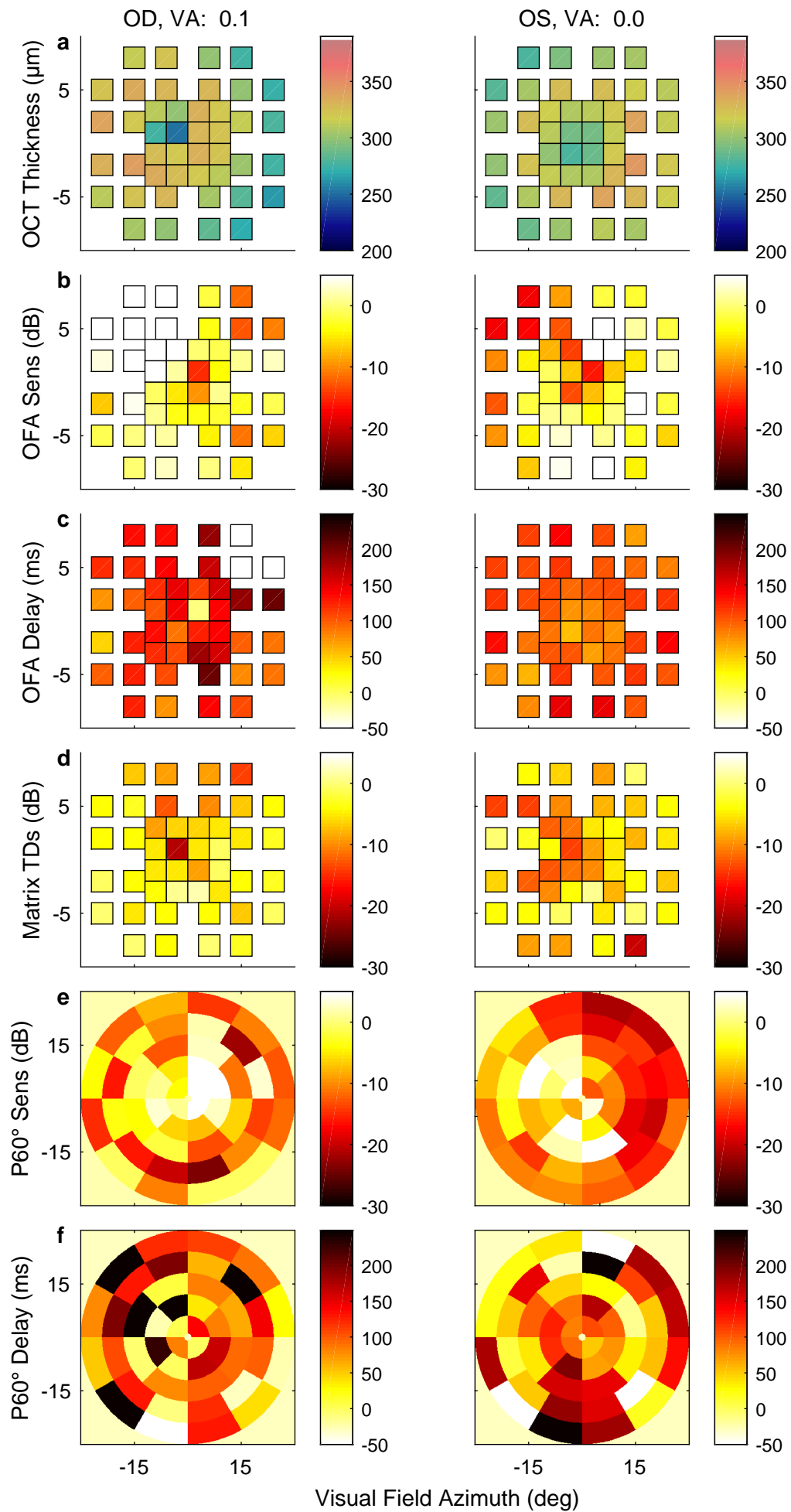

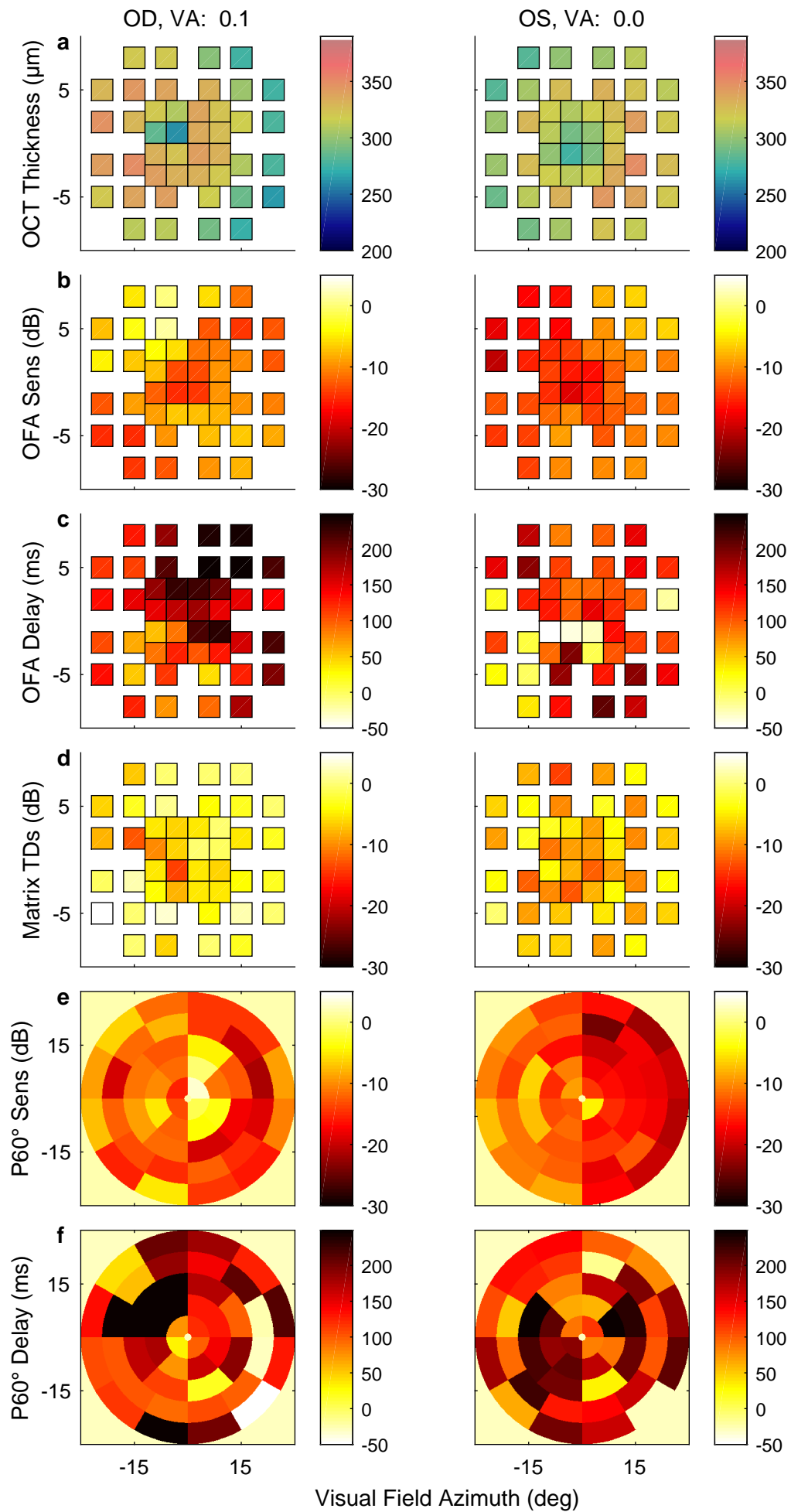

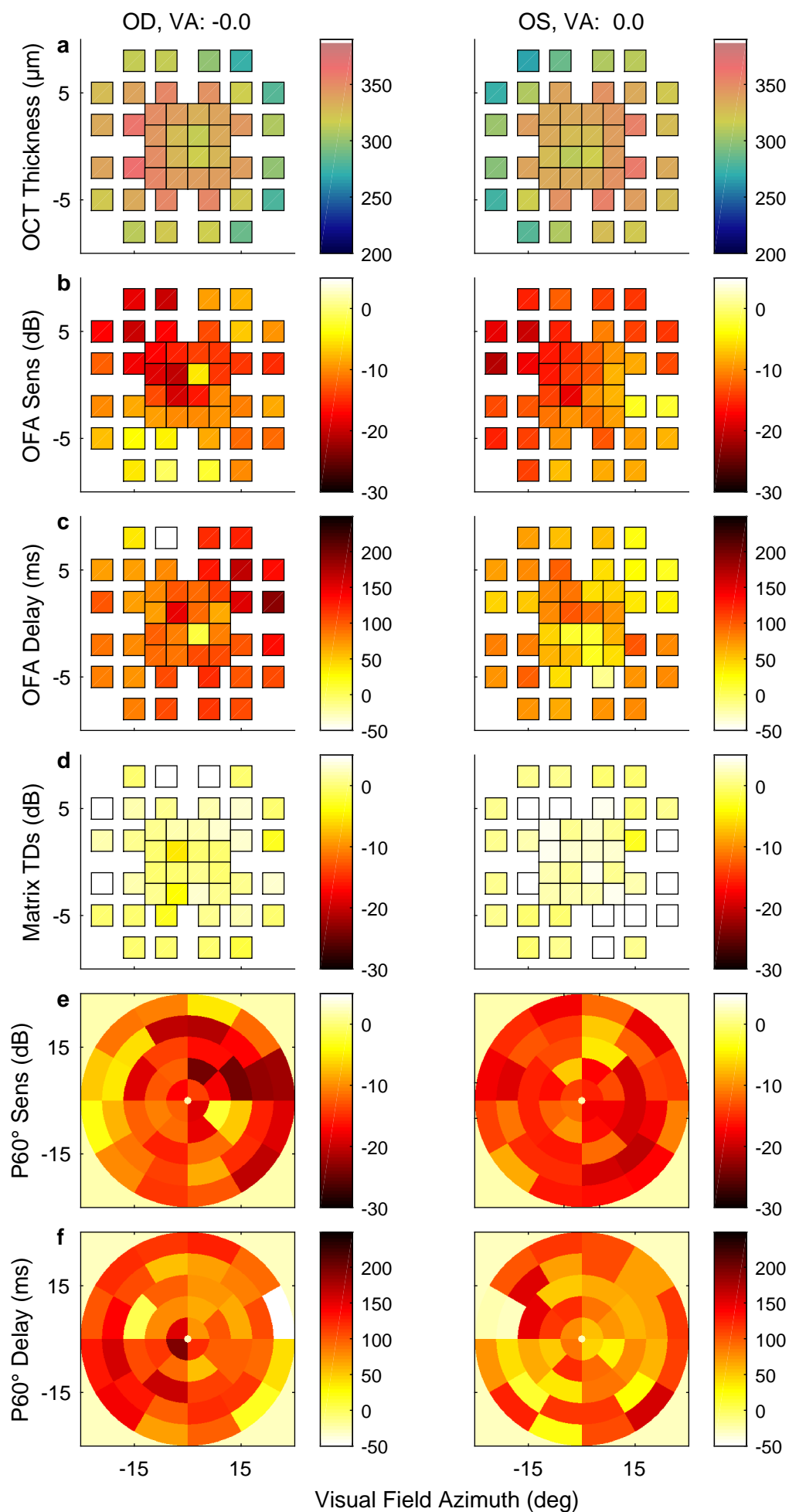

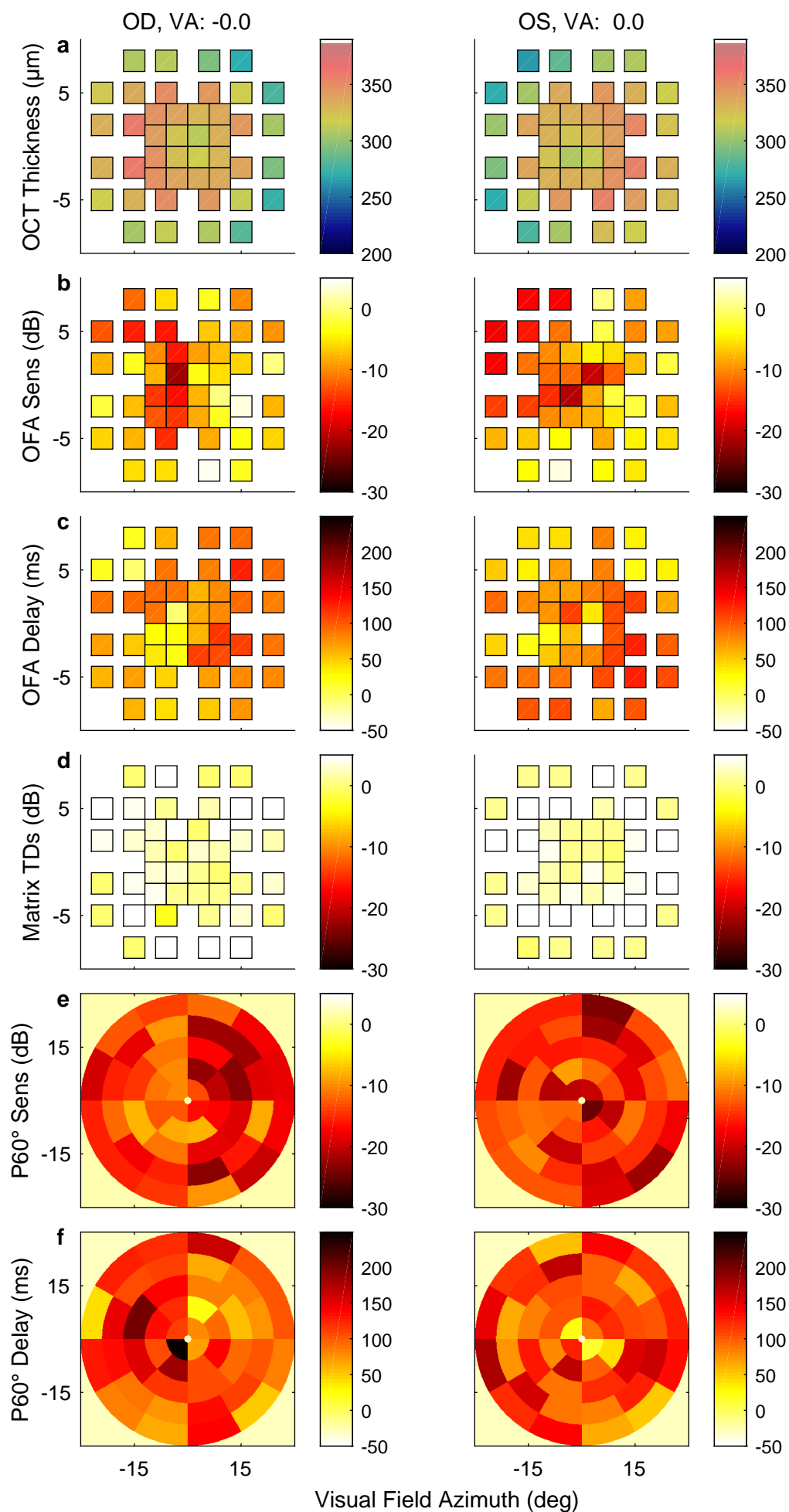

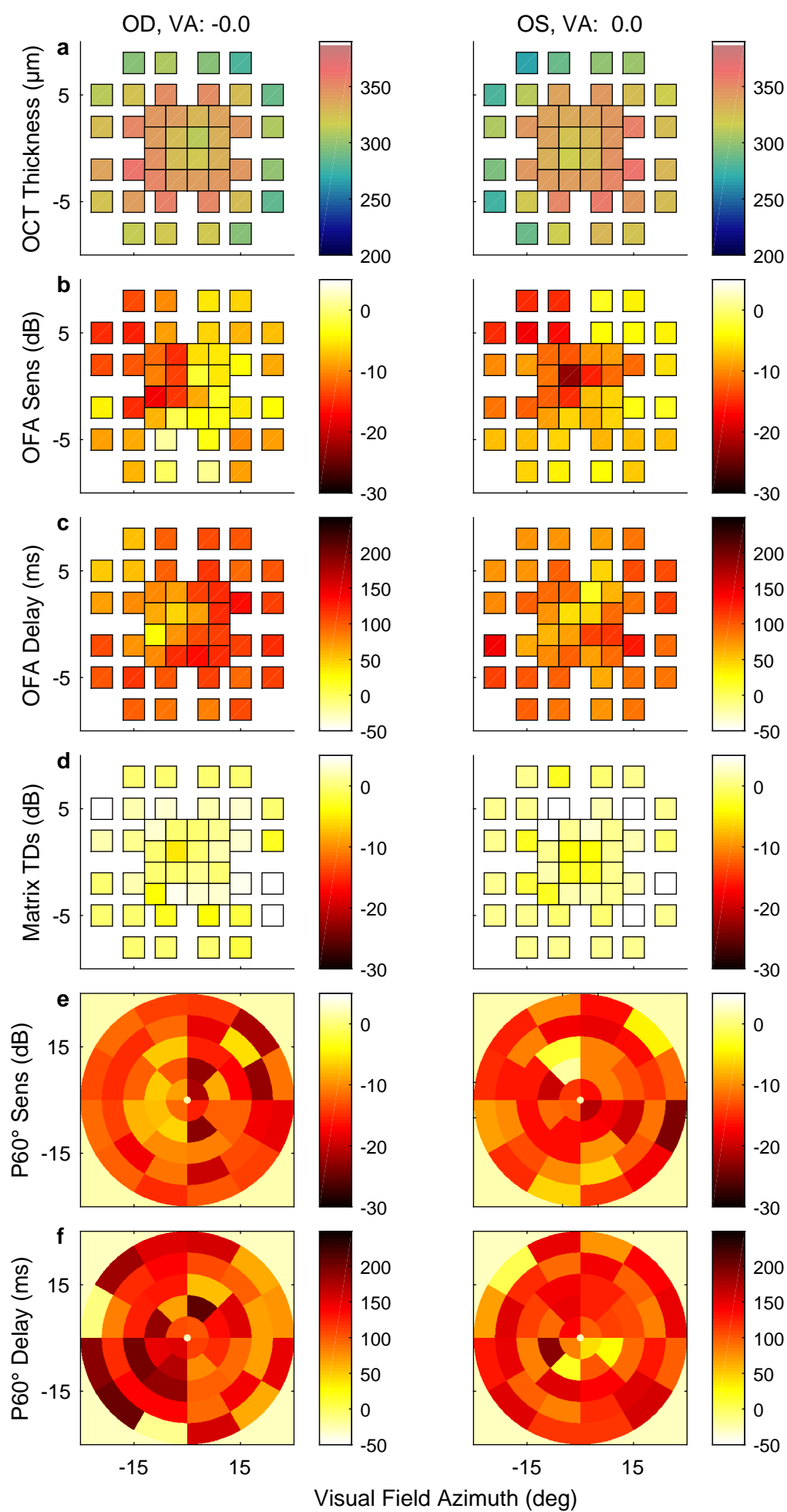

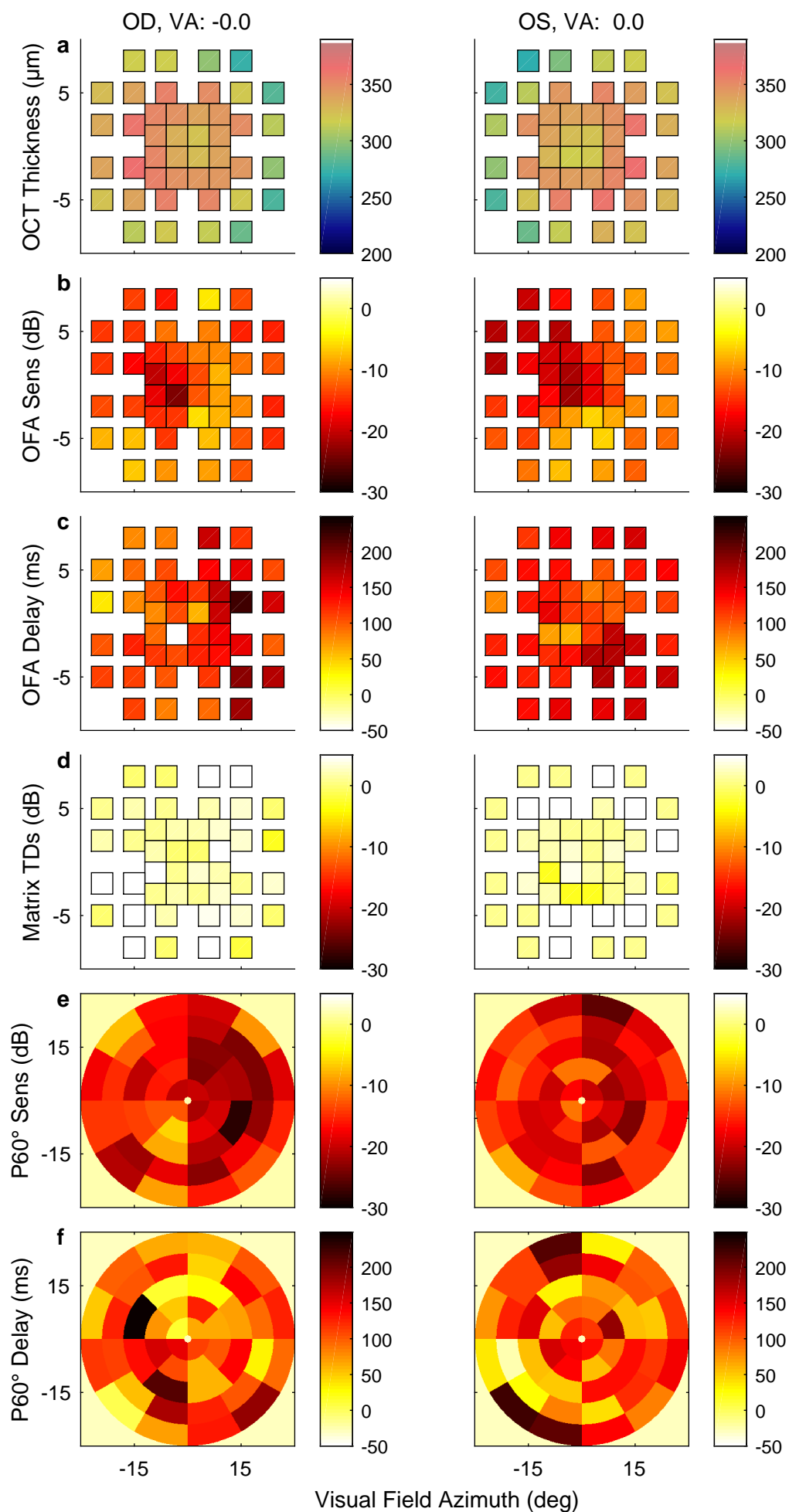

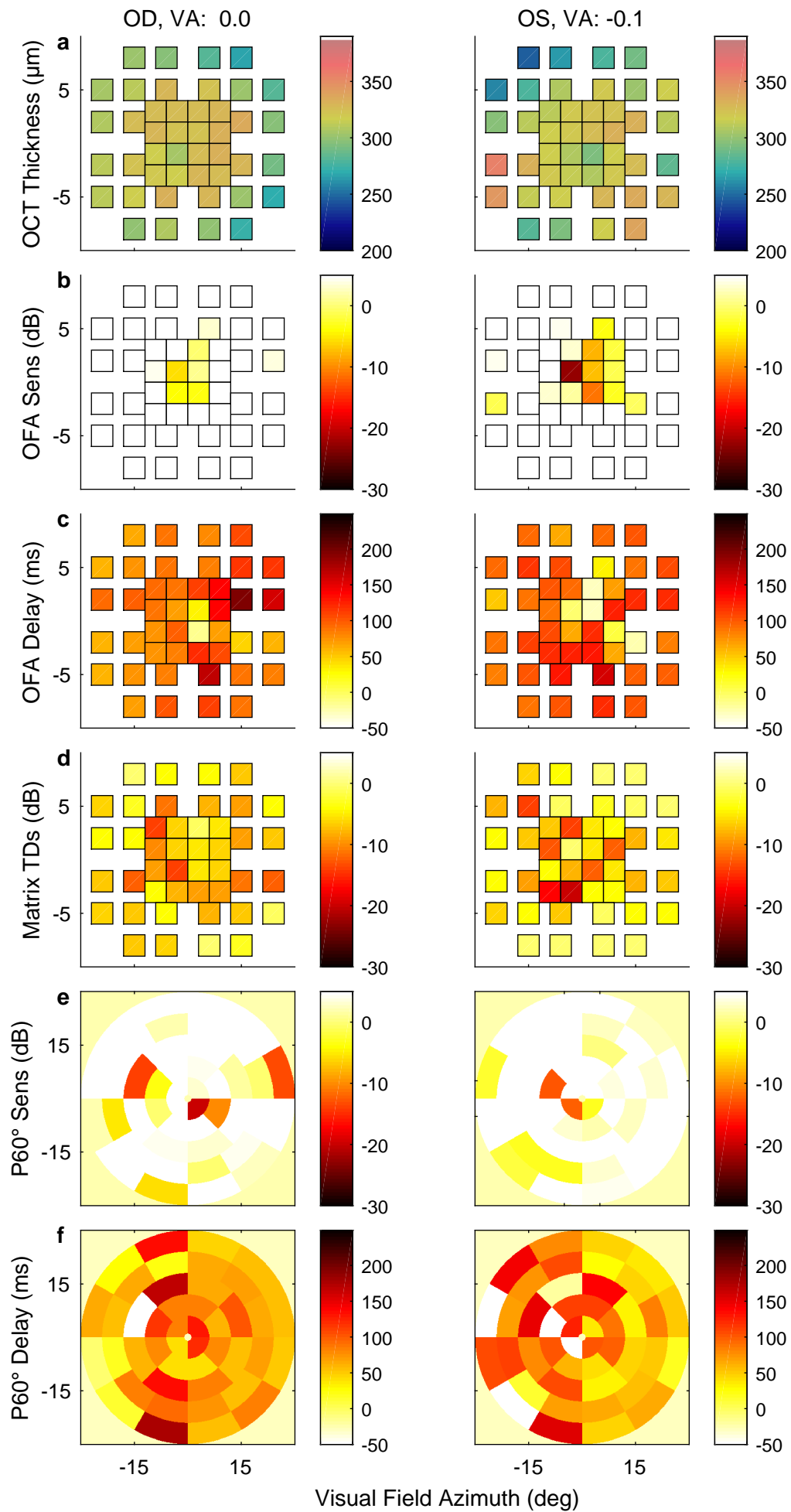

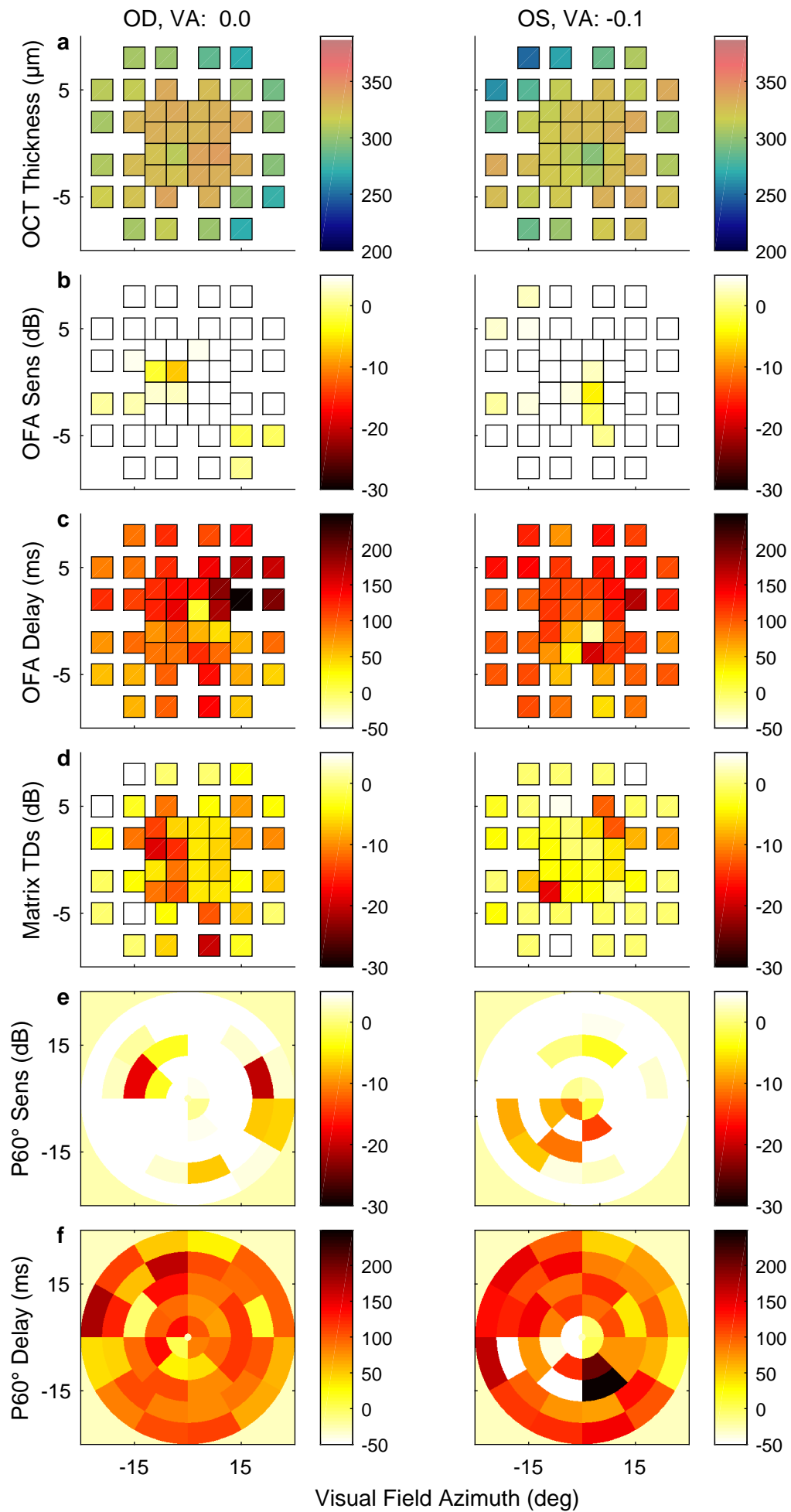

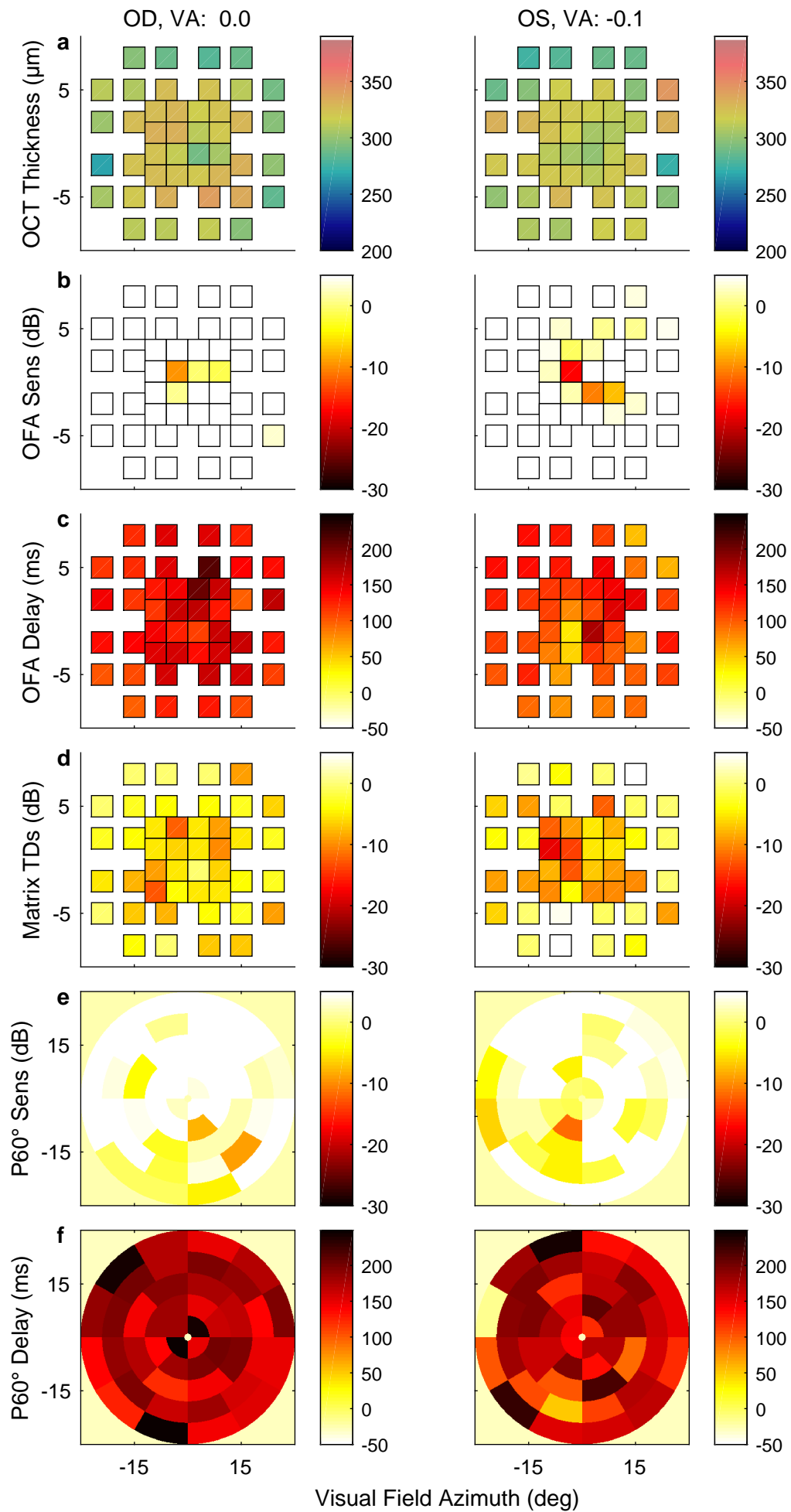

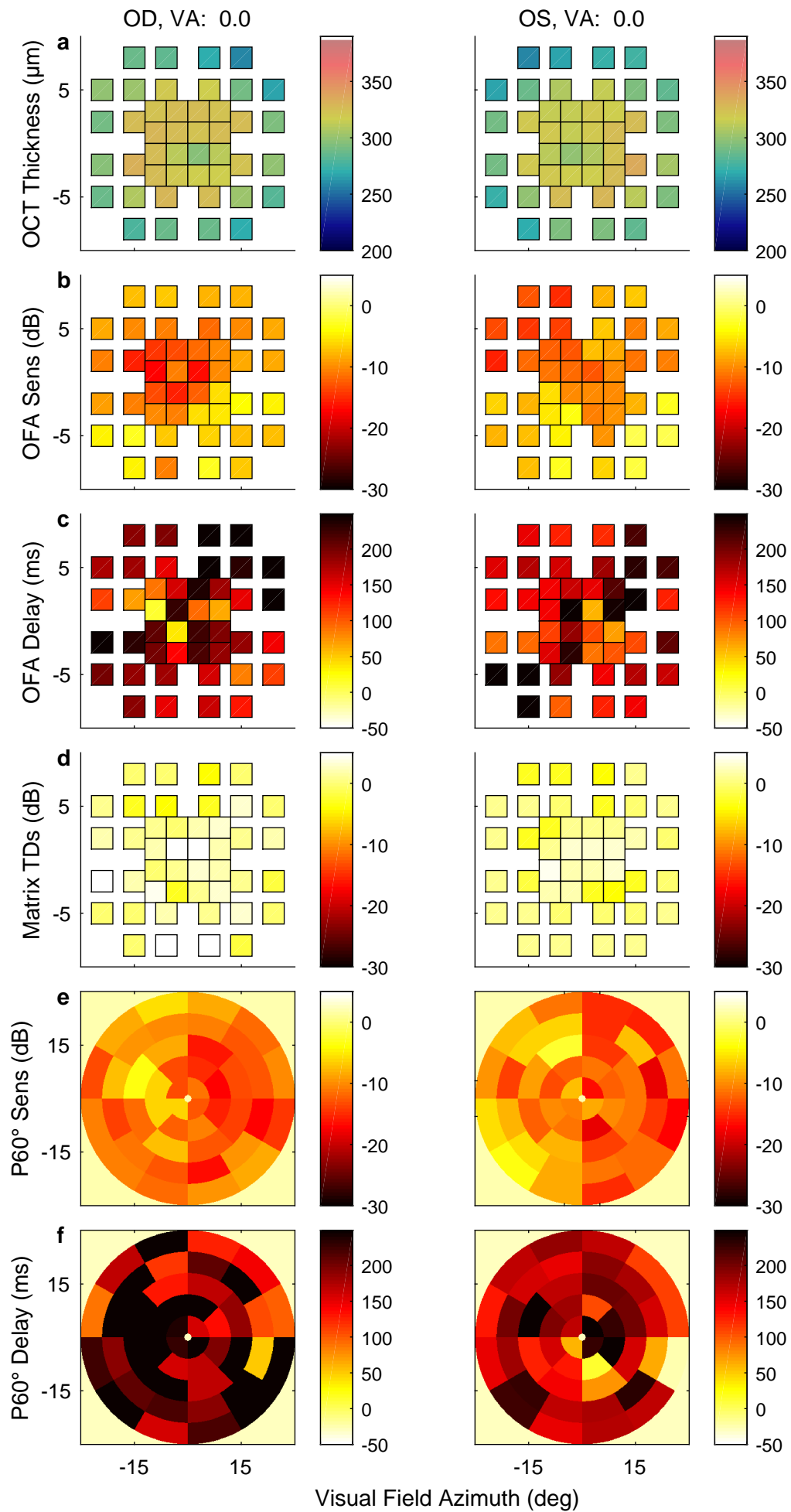

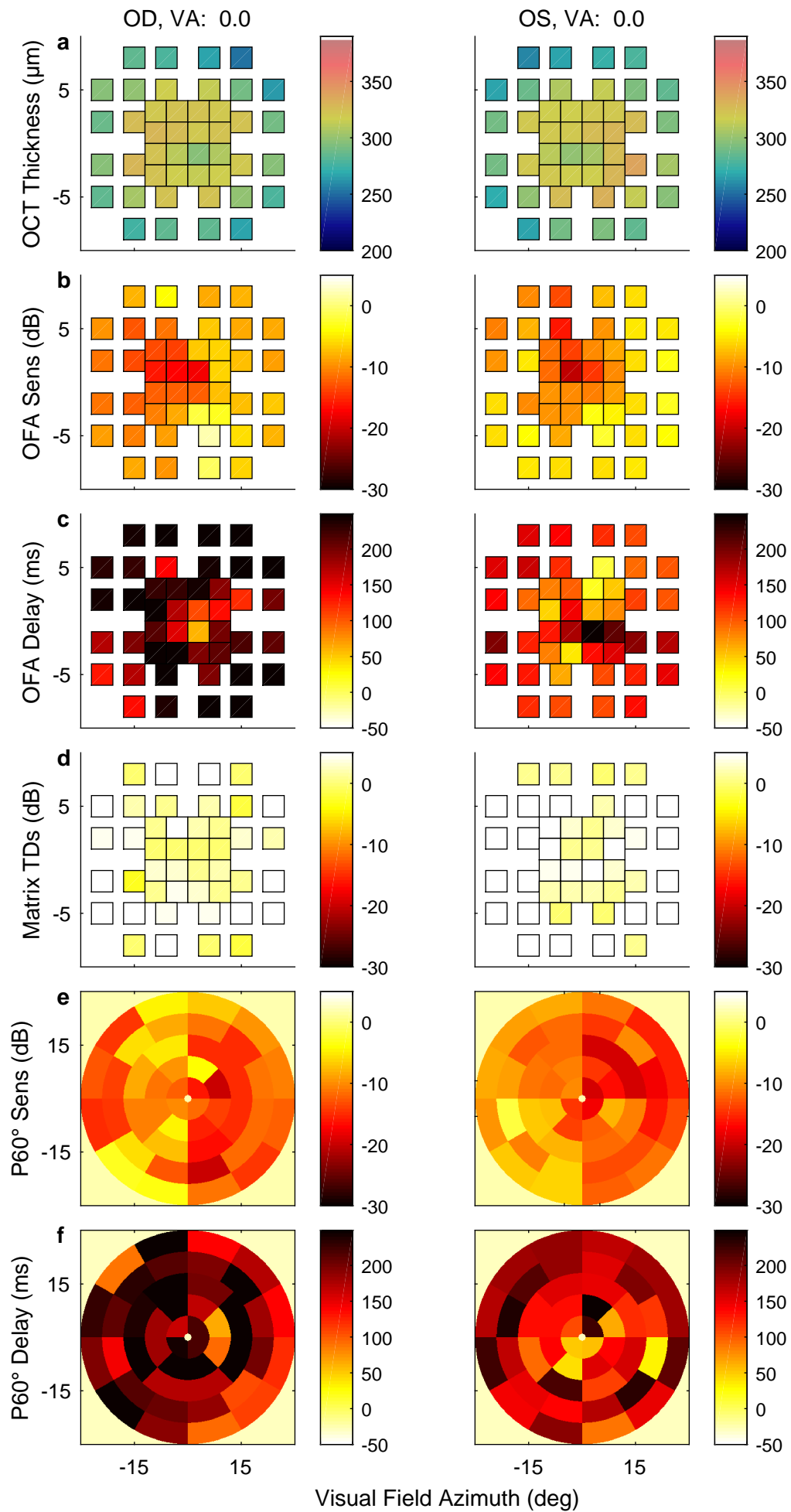

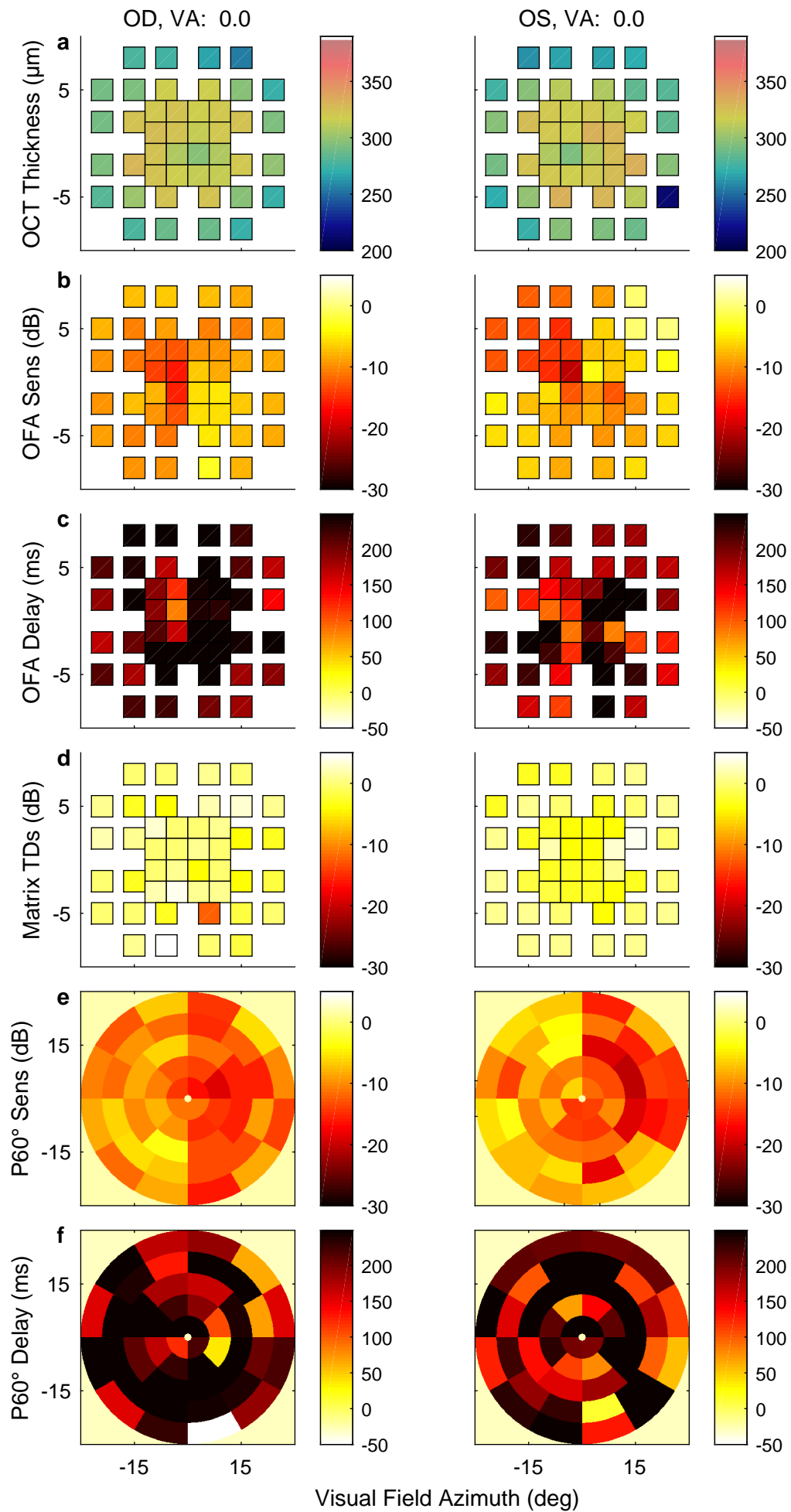

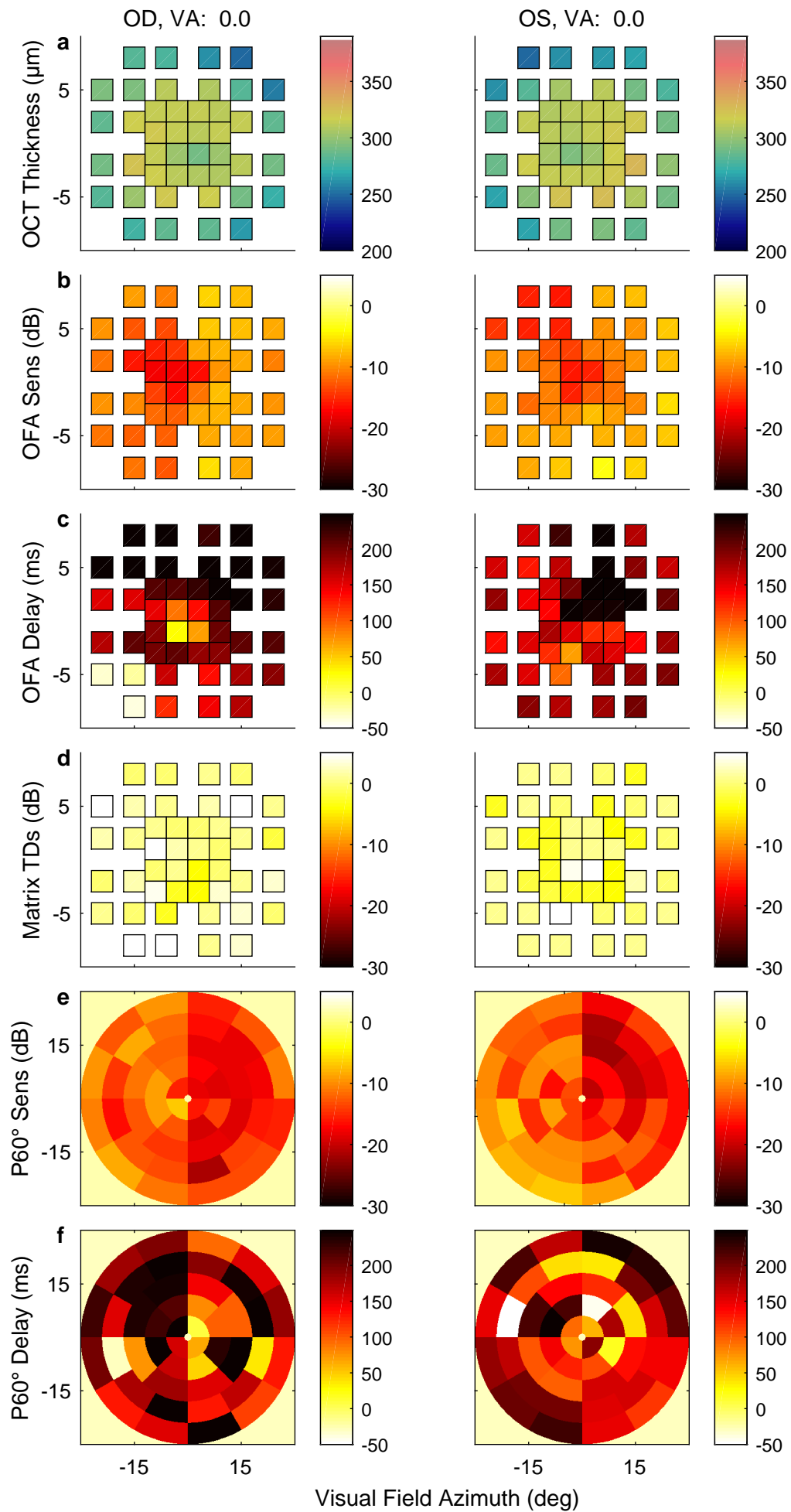

Supplement: S1 Fig — (PDF) [file pone.0287319.s001.pdf]

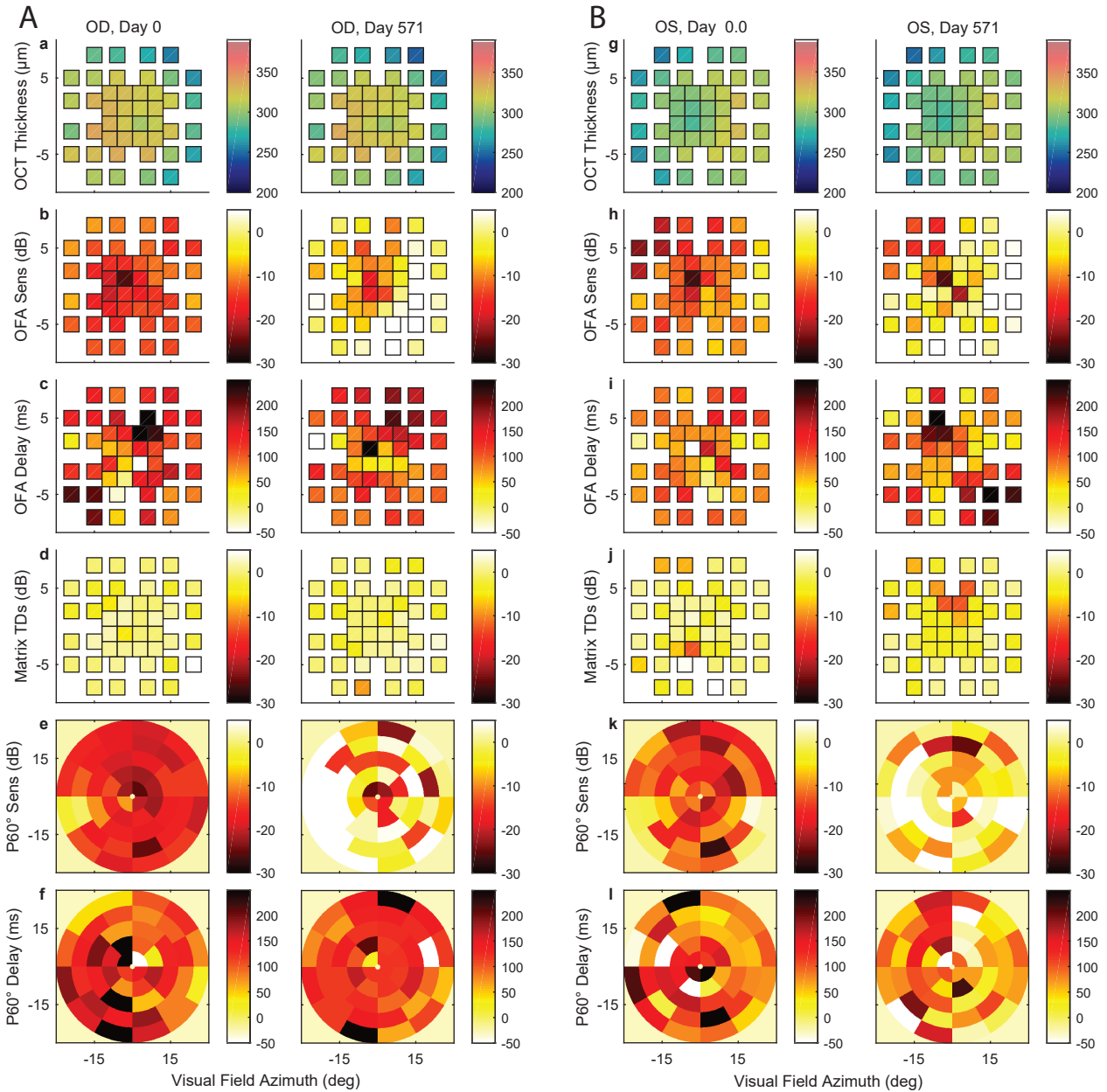

Supplement: S2 Fig — The last two rows show the sensitivity and delay results for the wide-field P60° stimulus, which cannot be mapped onto the 10–2 grid. Unlike Fig 2 data for the first and last visit are grouped by eye. (PDF) [file pone.0287319.s002.pdf]
