# Supplementary material for: Objective perimetry identifies regional functional progression and recovery in mild Diabetic Macular Oedema
Source: PLoS One. 2023 Jun 15;18(6):e0287319. doi: 10.1371/journal.pone.0287319 (PMC10270604; doi:10.1371/journal.pone.0287319)
Supplement: Attachment — Submitted filename: plos one review.pdf [file pone.0287319.s004.pdf]

This study by Sabeti et al presents interesting research findings regarding the comparison of structure function correlation in subjects with DMO. While findings are useful, it is not presented in a very readable manner. Many places assumes that reader would understand, especially the figures or regarding the variation in slopes. More details at specific locations have been requested. The presentation of data is really neat and unique. However, authors make further attempt to clarify more information in the figure legend. Important points have been made in bold.

Abstract:

Please include 1 sentence rationale for the study in the Purpose section.

Methods

What is the time interval between visits.

What is mfPOP variants?

Results

The reduction in retinal thickness seem very small. How is that deemed significant. Which test was used?

What delay are the authors referring to ? It is arbitrary. Numbers should be provided for reduction in retinal thicknesses over time.

“Matrix perimetry quantified many fewer regions that changed significantly.” Need to more specific.

How are conclusions supported by the results? Without providing the result in the abstract and claiming the conclusion does not seem appropriate.

Introduction

Line 3 DR is also functional manifestation?

Line 7 Need to qualify what is fenofibrate?

What is mild “off-centre” DMO?

Lines 14-16 limitations of erg and perimetry should be qualified respectively not grouped together as done presently.

Authors need to justify why not mf ERG which is also objective and gives focal responses? Why mfPOP over mf ERG?

Line 19-21 ; sentence construction needs to be addressed.

Lines 26-29 : how is it relevant in the present context.

The introduction is not flowing well. The exact rationale for the study not coming. Connecting sentences are missing. There are three separate chunks of paragraphs.

Lines 34-40: not suited for this context. Mostly methods related information in the introduction does not fit well.

40-42 :poorly written hypothesis; need to state why you are expecting something.

METHODS;

47-48: not agreeing with statement that both eyes had DMO

More clear information on the how the subjects were recruited needs to be provided.

Lines59-60: Exclusion criteria were absence of XXX conditions seem to suggest the opposite. Please check

Line 62: A set protocol was not followed for each of the patients in terms of the follow-up?

Line 108 : what is “significantly faster or slower than normal”?

Line 138: Expand further on how AmpStd μm to decibel sensitivity (dB)?

Line 191: What was defined as “significant change”?

Correlation heat map of OFA sensitivity changes with OCT thickness changes may be done as done in reference 25

Lines 215 -225: Which points had significant slopes? This section is not clear with respect to the figure. Please expand further.

Line 268-270: “Long-term follow-up of Matrix sensitivity in our cohort showed little significant change over time and did not reflect changes in retinal structure”

Even the OFA sensitivity did not show point-by-point correspondence with change in retinal thicknesses?

What is the implication of delay observed in pupil response?

Please clarify
“In automated perimetry measuring a sensitivity increase requires near super-human contrast sensitivity, whereas for OFA only an increase in a large objective response is required”
Line 276 It is Feigl not Feigle

Why only 10 subjects data presented instead of 16?

Comments on Figures

Fig 1

What are the dashed lines? What does it mean?

Can the rings be clearly demarcated?

Not clear about overlap of stimuli. Please explain clearly.

Panels B and D shows the two halves in each panel are in unequal size. Why is that?

“Reducing the luminance in regions with inherently higher sensitivity enhances the responses in less-responsive regions”

This is not clear and does not belong in the Figure legend.

Mention that there are 11 per each quadrant.

Fig 2

8X8 refers to mm?

Why are the blue panels in panel A tilted?

IMPORTANT: The regions where there is overlap of more than one box; how is the corresponding OCT thickness/OFA parameters considered?

Fig 3

Abstract says 16 patients with baseline DMO but the table 1 and Fig 3 says non-DMO subjects/ Needs fixing

Fig 4

Why this subject data was chosen to present?

Difference map between Visit 4 and Visit 1 may be more useful. Visually, it is difficult to compare so many individual points across the visits.

What is meant by “mapped to the 10-2 format”

What does the square inside represent.? In each of the panels?

What is on the x axis for panels a to d and g to j?

What does Y axis on the left indicate? – 5 and 5 ?

I would keep both visits next to each other for each eye separately for easy comparison?

Line 163 says day 517 and figure says 571

Fig 5

Which slopes are the authors referring to ?

OCT “Thickness” it should be?

What about the remaining 6 subjects?

What is the aim of this figure?

Authors should aim to highlight if retinal thickness and OFA sensitivity region is corresponding to the same points?

Fig 5. In heat map guide what does white mean? No change; it is clearly seen in guide in Fig 6 but not in Fig 5. Please change.

But the heat map does not seem to indicate that?

Why matrix perimetry seems largely unchanged but the retinal thickness changed noticeably in at least 3 subjects?
