# Supplementary material for: Objective perimetry identifies regional functional progression and recovery in mild Diabetic Macular Oedema
Source: PLoS One. 2023 Jun 15;18(6):e0287319. doi: 10.1371/journal.pone.0287319 (PMC10270604; doi:10.1371/journal.pone.0287319)
Supplement: Attachment — Submitted filename: Revisions2.pdf [file pone.0287319.s006.pdf]

Thank you for your responses.

Many places in the revised manuscript newly added text are not highlighted in red (for instance line 7-10 and line 37-40) and ideally where does the new text sit in the revised manuscript (in terms of line numbers) needs to be provided. It makes it difficult to identify and track the changes for the reviewer! Authors have misunderstood some of the abstract-related comments and addressed as if it were directed at the body of the paper. Please correct accordingly.

There are still methodological details missing. The results can be improved in terms of ease of reading.

1. Consider rewriting the below sentence

"Subjects were assessed at a median of 3 visits over 457 ± 198 days (mean ± SD) 75 with a median interval of 243.5 ± 145.6 (median ± SD) between visits"

Median is usually supplied with IQR. Mean and median used interchangeably. Also mention in the abstract.

"We enrolled T2D patients with mild DMO, and similar T2D patients without DMO at baseline, and

followed them longitudinally for 1.5 years."

A version of this statement needs to be in abstract methods

2. Expand mfpop and OFA in abstract methods

3. "Matrix perimetry quantified many fewer regions that changed significantly"

   Which regions need to be mentioned at least grossly

4. Authors need to justify why not mf ERG which is also objective and gives focal responses?

Why mfPOP over mf ERG? The justification is not sufficient. Is any distinct advantage?

5. Line 50 need to provide references for previous findings

6. P 30 and P 60 which one is subtending 15 and 30 degrees? Please clarify.

7. "many fewer" in the abstract results sounds paradoxical. Please change.

   Lines 109-110

8. "Total stimulus duration was 6 minutes divided into 9 segments of 40s with short breaks between segments"
   Assuming short break is 10 seconds; close to additional 1 min and 20 seconds is added to the protocol. Therefore the total testing time should be mentioned as 7 to 8 minutes.

Methods:
Important points

Spatial extent of DMO (in degrees) in each of the patients' needs to be quantified at baseline and the follow up visit. Preferably as table.

OFF centre DMO – how much was it off? How many degrees away from the macula? Need to be quantified.

This has direct implications on the structure-function mapping. That can help in understand how some points recover and not others.

Fig 4 suggestion

Keep OD basliene and follow up together instead of OD and OS together. Applies to OS as well,

If the DMO is well restricted within the macula, why are the peripheral points showing improvement or deterioration? In Fig 5 and 6

Fig 4 legend says it is Diabetic patient but the patient is DMO patient. Needs to be changed

Fig 5
Each parameter increase or decrease may mean deterioration or recovery

Increase in sensitivity is improvement but increase in thickness is perhaps worsening

So one suggestion is to add alphabet R (recovery) or W (worsening) at either end of the legend for each variable to help the reader. In fact letter choice is up to the authors

Difference is taken baseline – last visit or vice versa?. This information needs to be present in both Fig 5 and 6 legend.

Discussion point

What are the implications of delays?

What mechanisms is responsible for the delays? Can you point to me if it has been already mentioned explicitly in the paper?
