# Supplementary material for: Objective perimetry identifies regional functional progression and recovery in mild Diabetic Macular Oedema
Source: PLoS One. 2023 Jun 15;18(6):e0287319. doi: 10.1371/journal.pone.0287319 (PMC10270604; doi:10.1371/journal.pone.0287319)
Supplement: Attachment — Submitted filename: Revisions3.pdf [file pone.0287319.s009.pdf]

I am pleased that authors have taken efforts to address these key points.

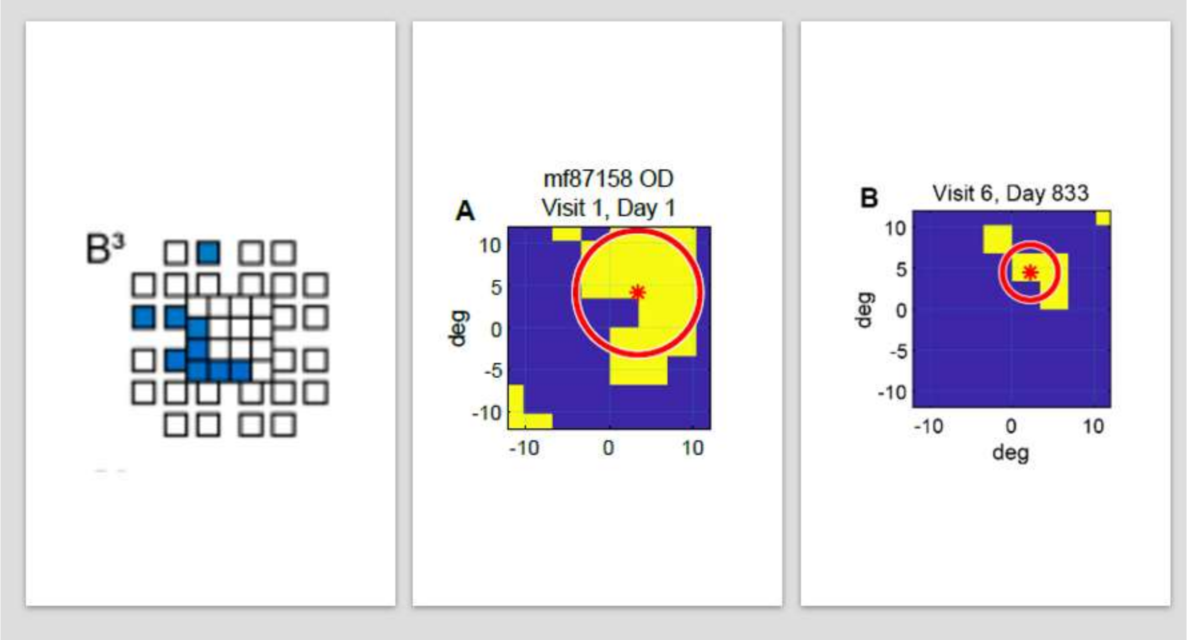

The new panels A and B (Fig 6) shows the changes in oedema seem like a mirror image of change in OCT retinal thickness in the left most panel (Fig 5). This is probably due to flipping of data to represent the corresponding region in visual space.

Therefore, add a note in Fig 6 legend that the data is presented here in retinal space.

Panel E legend not accurate. It shows all centroids not just those greater than 10 degrees
