# Supplementary material for: Objective perimetry identifies regional functional progression and recovery in mild Diabetic Macular Oedema
Source: PLoS One. 2023 Jun 15;18(6):e0287319. doi: 10.1371/journal.pone.0287319 (PMC10270604; doi:10.1371/journal.pone.0287319)
Supplement: Attachment — Submitted filename: Aran Sabeti et al.docx [file pone.0287319.s003.docx]

Aran Sabeti et al, al present an interesting and very well written study that evaluates functional progression and recovery in mild Diabetic Macular Oedema

The study results certainly suggest some degrees that Changes in retinal function measured by OFA possibly offer greater power to monitor DMO over time than Matrix perimetry data.

Besides how the magnitude of these data add new findings compare to the current standard cannot be determined based on this study

The results are encouraging and further study is warranted.

here some relevant points :

1. please add on the keywords this does not match with the manuscript

2. The authors should express why is relevant :Perimetry

in DME in a more standardized way please add it on the manuscript .What does it Change for the current standard of care ?

3. The authors should explain why their findings make a different for ophthalmologist

around the world and for the readers of *Plos one*

4. The authors should explain the source of the information and what were the criteria they used for adding to the paper Were the assessors masked? What was the ICC between them in orden to analyzed the data ? I Was the randomization digitalized?

5. Please add references and rephrase the sentence. English grammar should be applied.

“management protocol recommended by their ophthalmologist. For this reason, persons with DMO were followed up somewhat more frequently. Given the DMO patients were getting more frequent  updates about their ocular health than in their normal care, it was possible that some would  spontaneously improve their glycaemic control with resulting partial resolution of their DMO. This was helpful in the sense that we could examine whether the Matrix and OFA functional data were  correlated with either improved or worsening retinal thickness. “

6. please add in the introduction one line of previous papers published showing clinical capabilities that specifically localize and delineate pathology and ancillary test such and OCT in order to help physician and Retina specialist to properly diagnose the DME

These papers should discussed briefly and be referenced in the discussion part .

Reference these :

-Retina. 2019 Jan;39(1):44-51. doi: 10.1097/IAE.0000000000002196.

**DEXAMETHASONE IMPLANT FOR DIABETIC MACULAR EDEMA IN NAIVE COMPARED WITH REFRACTORY EYES: The International Retina Group Real-Life 24-Month Multicenter Study. The IRGREL-DEX Study.**

[Iglicki M](https://www.ncbi.nlm.nih.gov/pubmed/?term=Iglicki%20M%5BAuthor%5D&cauthor=true&cauthor_uid=29697589)1, [Busch C](https://www.ncbi.nlm.nih.gov/pubmed/?term=Busch%20C%5BAuthor%5D&cauthor=true&cauthor_uid=29697589)2, [Zur D](https://www.ncbi.nlm.nih.gov/pubmed/?term=Zur%20D%5BAuthor%5D&cauthor=true&cauthor_uid=29697589)3,4, [Okada M](https://www.ncbi.nlm.nih.gov/pubmed/?term=Okada%20M%5BAuthor%5D&cauthor=true&cauthor_uid=29697589)5, [Mariussi M](https://www.ncbi.nlm.nih.gov/pubmed/?term=Mariussi%20M%5BAuthor%5D&cauthor=true&cauthor_uid=29697589)6, [Chhablani JK](https://www.ncbi.nlm.nih.gov/pubmed/?term=Chhablani%20JK%5BAuthor%5D&cauthor=true&cauthor_uid=29697589)7, [Cebeci Z](https://www.ncbi.nlm.nih.gov/pubmed/?term=Cebeci%20Z%5BAuthor%5D&cauthor=true&cauthor_uid=29697589)8, [Fraser-Bell S](https://www.ncbi.nlm.nih.gov/pubmed/?term=Fraser-Bell%20S%5BAuthor%5D&cauthor=true&cauthor_uid=29697589)9, [Chaikitmongkol V](https://www.ncbi.nlm.nih.gov/pubmed/?term=Chaikitmongkol%20V%5BAuthor%5D&cauthor=true&cauthor_uid=29697589)10, [Couturier A](https://www.ncbi.nlm.nih.gov/pubmed/?term=Couturier%20A%5BAuthor%5D&cauthor=true&cauthor_uid=29697589)11, [Giancipoli E](https://www.ncbi.nlm.nih.gov/pubmed/?term=Giancipoli%20E%5BAuthor%5D&cauthor=true&cauthor_uid=29697589)12, [Lupidi M](https://www.ncbi.nlm.nih.gov/pubmed/?term=Lupidi%20M%5BAuthor%5D&cauthor=true&cauthor_uid=29697589)13, [Rodríguez-Valdés PJ](https://www.ncbi.nlm.nih.gov/pubmed/?term=Rodr%C3%ADguez-Vald%C3%A9s%20PJ%5BAuthor%5D&cauthor=true&cauthor_uid=29697589)14, [Rehak M](https://www.ncbi.nlm.nih.gov/pubmed/?term=Rehak%20M%5BAuthor%5D&cauthor=true&cauthor_uid=29697589)2, [Fung AT](https://www.ncbi.nlm.nih.gov/pubmed/?term=Fung%20AT%5BAuthor%5D&cauthor=true&cauthor_uid=29697589)15,16,17, [Goldstein M](https://www.ncbi.nlm.nih.gov/pubmed/?term=Goldstein%20M%5BAuthor%5D&cauthor=true&cauthor_uid=29697589)3,4, [Loewenstein A](https://www.ncbi.nlm.nih.gov/pubmed/?term=Loewenstein%20A%5BAuthor%5D&cauthor=true&cauthor_uid=29697589)3,4,17.

-Acta Diabetol. 2018 Jun;55(6):541-547. doi: 10.1007/s00592-018-1117-z. Epub 2018 Mar 1.

**Progression of diabetic retinopathy severity after treatment with dexamethasone implant: a 24-month cohort study the 'DR-Pro-DEX Study'.**

[Iglicki M](https://www.ncbi.nlm.nih.gov/pubmed/?term=Iglicki%20M%5BAuthor%5D&cauthor=true&cauthor_uid=29497837)1, [Zur D](https://www.ncbi.nlm.nih.gov/pubmed/?term=Zur%20D%5BAuthor%5D&cauthor=true&cauthor_uid=29497837)2,3, [Busch C](https://www.ncbi.nlm.nih.gov/pubmed/?term=Busch%20C%5BAuthor%5D&cauthor=true&cauthor_uid=29497837)4, [Okada M](https://www.ncbi.nlm.nih.gov/pubmed/?term=Okada%20M%5BAuthor%5D&cauthor=true&cauthor_uid=29497837)5, [Loewenstein A](https://www.ncbi.nlm.nih.gov/pubmed/?term=Loewenstein%20A%5BAuthor%5D&cauthor=true&cauthor_uid=29497837)2,3,6.

-Ophthalmologica. 2019;241(1):9-16. doi: 10.1159/000492132. Epub 2018 Nov 8.

**Effectiveness and Safety of Intravitreal Dexamethasone Implant (Ozurdex) in Patients with Diabetic Macular Edema: A Real-World Experience.**

[Mello Filho P](https://www.ncbi.nlm.nih.gov/pubmed/?term=Mello%20Filho%20P%5BAuthor%5D&cauthor=true&cauthor_uid=30408801)1, [Andrade G](https://www.ncbi.nlm.nih.gov/pubmed/?term=Andrade%20G%5BAuthor%5D&cauthor=true&cauthor_uid=30408801)2, [Maia A](https://www.ncbi.nlm.nih.gov/pubmed/?term=Maia%20A%5BAuthor%5D&cauthor=true&cauthor_uid=30408801)1, [Maia M](https://www.ncbi.nlm.nih.gov/pubmed/?term=Maia%20M%5BAuthor%5D&cauthor=true&cauthor_uid=30408801)1, [Biccas Neto L](https://www.ncbi.nlm.nih.gov/pubmed/?term=Biccas%20Neto%20L%5BAuthor%5D&cauthor=true&cauthor_uid=30408801)1, [Muralha Neto A](https://www.ncbi.nlm.nih.gov/pubmed/?term=Muralha%20Neto%20A%5BAuthor%5D&cauthor=true&cauthor_uid=30408801)3, [Moura Brasil O](https://www.ncbi.nlm.nih.gov/pubmed/?term=Moura%20Brasil%20O%5BAuthor%5D&cauthor=true&cauthor_uid=30408801)4, [Minelli E](https://www.ncbi.nlm.nih.gov/pubmed/?term=Minelli%20E%5BAuthor%5D&cauthor=true&cauthor_uid=30408801)5, [Dalloul C](https://www.ncbi.nlm.nih.gov/pubmed/?term=Dalloul%20C%5BAuthor%5D&cauthor=true&cauthor_uid=30408801)6, [Iglicki M](https://www.ncbi.nlm.nih.gov/pubmed/?term=Iglicki%20M%5BAuthor%5D&cauthor=true&cauthor_uid=30408801)7.

-Ophthalmic Res. 2019 May 2:1-6. doi: 10.1159/000499540. [Epub ahead of print]

**The Role of Steroids in the Management of Diabetic Macular Edema.**

[Zur D](https://www.ncbi.nlm.nih.gov/pubmed/?term=Zur%20D%5BAuthor%5D&cauthor=true&cauthor_uid=31048580)1, [Iglicki M](https://www.ncbi.nlm.nih.gov/pubmed/?term=Iglicki%20M%5BAuthor%5D&cauthor=true&cauthor_uid=31048580)2, [Loewenstein A](https://www.ncbi.nlm.nih.gov/pubmed/?term=Loewenstein%20A%5BAuthor%5D&cauthor=true&cauthor_uid=31048580)3.

-Acta Diabetol. 2019 Oct;56(10):1141-1147. doi: 10.1007/s00592-019-01357-y. Epub 2019 May 14.

**TRActional DIabetic reTInal detachment surgery with co-adjuvant intravitreal dexamethasONe implant: the TRADITION STUDY.**

-[Iglicki M](https://www.ncbi.nlm.nih.gov/pubmed/?term=Iglicki%20M%5BAuthor%5D&cauthor=true&cauthor_uid=31089929)1, [Zur D](https://www.ncbi.nlm.nih.gov/pubmed/?term=Zur%20D%5BAuthor%5D&cauthor=true&cauthor_uid=31089929)2,3, [Fung A](https://www.ncbi.nlm.nih.gov/pubmed/?term=Fung%20A%5BAuthor%5D&cauthor=true&cauthor_uid=31089929)4,5,6, [Gabrielle PH](https://www.ncbi.nlm.nih.gov/pubmed/?term=Gabrielle%20PH%5BAuthor%5D&cauthor=true&cauthor_uid=31089929)7, [Lupidi M](https://www.ncbi.nlm.nih.gov/pubmed/?term=Lupidi%20M%5BAuthor%5D&cauthor=true&cauthor_uid=31089929)8, [Santos R](https://www.ncbi.nlm.nih.gov/pubmed/?term=Santos%20R%5BAuthor%5D&cauthor=true&cauthor_uid=31089929)9, [Busch C](https://www.ncbi.nlm.nih.gov/pubmed/?term=Busch%20C%5BAuthor%5D&cauthor=true&cauthor_uid=31089929)10, [Rehak M](https://www.ncbi.nlm.nih.gov/pubmed/?term=Rehak%20M%5BAuthor%5D&cauthor=true&cauthor_uid=31089929)10, [Cebeci Z](https://www.ncbi.nlm.nih.gov/pubmed/?term=Cebeci%20Z%5BAuthor%5D&cauthor=true&cauthor_uid=31089929)11, [Charles M](https://www.ncbi.nlm.nih.gov/pubmed/?term=Charles%20M%5BAuthor%5D&cauthor=true&cauthor_uid=31089929)12, [Masarwa D](https://www.ncbi.nlm.nih.gov/pubmed/?term=Masarwa%20D%5BAuthor%5D&cauthor=true&cauthor_uid=31089929)2,3, [Schwarz S](https://www.ncbi.nlm.nih.gov/pubmed/?term=Schwarz%20S%5BAuthor%5D&cauthor=true&cauthor_uid=31089929)2,3, [Barak A](https://www.ncbi.nlm.nih.gov/pubmed/?term=Barak%20A%5BAuthor%5D&cauthor=true&cauthor_uid=31089929)2,3, [Loewenstein A](https://www.ncbi.nlm.nih.gov/pubmed/?term=Loewenstein%20A%5BAuthor%5D&cauthor=true&cauthor_uid=31089929)2,3,13; [International Retina Group (IRG)](https://www.ncbi.nlm.nih.gov/pubmed/?term=International%20Retina%20Group%20(IRG)%5BCorporate%20Author%5D).

-Acta Ophthalmol. 2019 Aug 17. doi: 10.1111/aos.14230. [Epub ahead of print]

**Disorganization of retinal inner layers as a biomarker in patients with diabetic macular oedema treated with dexamethasone implant.**

[Zur D](https://www.ncbi.nlm.nih.gov/pubmed/?term=Zur%20D%5BAuthor%5D&cauthor=true&cauthor_uid=31421028)1, [Iglicki M](https://www.ncbi.nlm.nih.gov/pubmed/?term=Iglicki%20M%5BAuthor%5D&cauthor=true&cauthor_uid=31421028)2, [Sala-Puigdollers A](https://www.ncbi.nlm.nih.gov/pubmed/?term=Sala-Puigdollers%20A%5BAuthor%5D&cauthor=true&cauthor_uid=31421028)3, [Chhablani J](https://www.ncbi.nlm.nih.gov/pubmed/?term=Chhablani%20J%5BAuthor%5D&cauthor=true&cauthor_uid=31421028)4, [Lupidi M](https://www.ncbi.nlm.nih.gov/pubmed/?term=Lupidi%20M%5BAuthor%5D&cauthor=true&cauthor_uid=31421028)5, [Fraser-Bell S](https://www.ncbi.nlm.nih.gov/pubmed/?term=Fraser-Bell%20S%5BAuthor%5D&cauthor=true&cauthor_uid=31421028)6, [Mendes TS](https://www.ncbi.nlm.nih.gov/pubmed/?term=Mendes%20TS%5BAuthor%5D&cauthor=true&cauthor_uid=31421028)7,8, [Chaikitmongkol V](https://www.ncbi.nlm.nih.gov/pubmed/?term=Chaikitmongkol%20V%5BAuthor%5D&cauthor=true&cauthor_uid=31421028)9, [Cebeci Z](https://www.ncbi.nlm.nih.gov/pubmed/?term=Cebeci%20Z%5BAuthor%5D&cauthor=true&cauthor_uid=31421028)10, [Dollberg D](https://www.ncbi.nlm.nih.gov/pubmed/?term=Dollberg%20D%5BAuthor%5D&cauthor=true&cauthor_uid=31421028)1, [Busch C](https://www.ncbi.nlm.nih.gov/pubmed/?term=Busch%20C%5BAuthor%5D&cauthor=true&cauthor_uid=31421028)11, [Invernizzi A](https://www.ncbi.nlm.nih.gov/pubmed/?term=Invernizzi%20A%5BAuthor%5D&cauthor=true&cauthor_uid=31421028)12,13, [Habot-Wilner Z](https://www.ncbi.nlm.nih.gov/pubmed/?term=Habot-Wilner%20Z%5BAuthor%5D&cauthor=true&cauthor_uid=31421028)1, [Loewenstein A](https://www.ncbi.nlm.nih.gov/pubmed/?term=Loewenstein%20A%5BAuthor%5D&cauthor=true&cauthor_uid=31421028)1,14; [International Retina Group (IRG)](https://www.ncbi.nlm.nih.gov/pubmed/?term=International%20Retina%20Group%20(IRG)%5BCorporate%20Author%5D).

Multicenter Study

Br J Ophthalmol.2020 May;104(5):666-671. doi: 10.1136/bjophthalmol-2019-314523. Epub 2019 Aug 7.

Outer retinal hyperreflective deposits (ORYD): a new OCT feature in naïve diabetic macular oedema after PPV with ILM peeling

-Ophthalmologica. 2019;241(1):9-16. doi: 10.1159/000492132. Epub 2018 Nov 8.Effectiveness and Safety of Intravitreal Dexamethasone Implant (Ozurdex) in Patients with Diabetic Macular Edema: A Real-World Experience.

Mello Filho P1, Andrade G2, Maia A1, Maia M1, Biccas Neto L1, Muralha Neto A3, Moura Brasil O4, Minelli E5, Dalloul C6, Iglicki M7.

-Ophthalmic Res. 2019 May 2:1-6. doi: 10.1159/000499540. [Epub ahead of print]

The Role of Steroids in the Management of Diabetic Macular Edema.

Zur D1, Iglicki M2, Loewenstein A3.

-Acta Diabetol. 2019 Oct;56(10):1141-1147. doi: 10.1007/s00592-019-01357-y. Epub 2019 May 14.

TRActional DIabetic reTInal detachment surgery with co-adjuvant intravitreal dexamethasONe implant: the TRADITION STUDY.

-Published online 2018 Jul 11. doi: 10.1371/journal.pone.0200365PMCID: PMC6040739PMID: 29995929 Biomarkers and predictors for functional and anatomic outcomes for small gauge pars plana vitrectomy and peeling of the internal limiting membrane in naïve diabetic macular edema: The VITAL Study

-Acta Diabetol2020 Apr 16. doi: 10.1007/s00592-020-01530-8. Online ahead of print.Results in comparison between 30 gauge ultrathin wall and 27 gauge needle in sutureless intraocular lens flanged technique in diabetic patients: 24-month follow-up study

-

Eye 2021 Aug 9. doi: 10.1038/s41433-021-01722-8. Online ahead of print.

Next-generation anti-VEGF agents for diabetic macular oedema

-Retina. 2019 Nov;39(11):2161-2166. doi: 10.1097/IAE.0000000000002270.

UNDERDIAGNOSED OPTIC DISK PIT MACULOPATHY: Spectral Domain Optical Coherence Tomography Features For Accurate Diagnosis

Matias Iglicki 1 , Catharina Busch 2 , Anat Loewenstein 3	4	5 , Adrian T Fung 6	7	8 , Alessandro Invernizzi 8	9 , Miriana Mariussi 10 , Romina Arias 1 , Pierre-Henry Gabrielle 11 , Zafer Cebeci 12 , Mali Okada 13 , Jerzy Nawrocki 14	15 , Zofia Michalewska 14	15 , Michaella Goldstein 3	4 , Adiel Barak 3	4 , Dinah Zur 3

-EYE-2021 Apr;35(4):1111-1116. doi: 10.1038/s41433-020-01309-9. Epub 2021 Jan 11.

-Ophthalmology.2018 Feb;125(2):267-275. doi: 10.1016/j.ophtha.2017.08.031. Epub 2017 Sep 19.

OCT Biomarkers as Functional Outcome Predictors in Diabetic Macular Edema Treated with Dexamethasone Implant

Eye 2022 Jan 18. doi: 10.1038/s41433-022-01931-9. Online ahead of print.

**Vitrectomized vs non-vitrectomized eyes in DEX implant treatment for DMO-Is there any difference? the VITDEX study**

**-EYE 2022 Aug 29. doi: 10.1038/s41433-022-02222-z. Online ahead of print.**

**Naïve subretinal haemorrhage due to neovascular age-related macular degeneration. pneumatic displacement, subretinal air, and tissue plasminogen activator: subretinal vs intravitreal aflibercept-the native study**

- Ophthalmol Retina.2021 Nov;5(11):1097-1106.

doi: 10.1016/j.oret.2021.01.013. Epub 2021 Feb 1.Detection of Diabetic Retinopathy from Ultra-Widefield Scanning Laser Ophthalmoscope Images: A Multicenter Deep Learning Analysis

7.please add how, and how long takes for a retina specialist to switch in their mind the mandatory use of Perimetry what position in the current algorithm of understanding, diagnosis and treatment of DME has or will have this technique

8. Results could be misinterpreted add a short summary of the similarities in different OCT devices and also add diferente oct modalities SD, swept source EDI etc and what can be improved in the process of detecting RAP is mandatory in the discussion section

9. Please apply correction for misspelling and English grammar

10 . This paper should be corrected by an english redactor
