# Supplementary material for: Objective perimetry identifies regional functional progression and recovery in mild Diabetic Macular Oedema
Source: PLoS One. 2023 Jun 15;18(6):e0287319. doi: 10.1371/journal.pone.0287319 (PMC10270604; doi:10.1371/journal.pone.0287319)
Supplement: Attachment — Submitted filename: Response to Reviewers_v2.docx [file pone.0287319.s005.docx]

**RESPONSE TO REVIEWER’S COMMENTS**

We are very grateful for the reviews of this manuscript provided by the editor and each of the reviewers. The comments are encouraging and the reviewers appear to share our consideration that this study and its results are clinically important. We appreciate all suggestions from the reviewers, and tried to clarify the purpose and demonstrate some possible limitations of our study. Please see below, in black fonts, our detailed response to comments. Amendments to manuscript highlighted in red.

Reviewer Comments:

**Reviewer #1:**

Faran Sabeti et al, al present an interesting and very well written study that evaluates functional progression and recovery in mild Diabetic Macular Oedema

The study results certainly suggest some degrees that Changes in retinal function measured by OFA possibly offer greater power to monitor DMO over time than Matrix perimetry data.

Besides how the magnitude of these data add new findings compare to the current standard cannot be determined based on this study

The results are encouraging and further study is warranted.

Thank you for your positive appraisal of our work. We agree that this work is still in it’s infancy and represents a preliminary investigation of the longitudinal changes in retinal function in patients with DMO measured by subjective and objective perimetry.

Here some relevant points :

1. please add on the keywords this does not match with the manuscript

Thank you for point this out, we have now updated keywords to match the manuscript.

2. The authors should express why is relevant: Perimetry in DME in a more standardized way please add it on the manuscript. What does it Change for the current standard of care?

The current standard of care in DME management only includes visual acuity as the primary measure of visual function; however, a growing body of evidence demonstrates that visual dysfunction precedes structural changes but only the central 1 deg of visual space is being functionally sampled by acuity. The advantage of mfPOP in measuring changes in retinal function in DME includes its rapid, easy to administer nature and that it identifies localised regions of retinal dysfunction that can be followed-up overtime. To convey this in the manuscript we have added the following statement to the Discussion:-

The advantage of mfPOP is that it is rapid and easily administered and objectively measures local retinal function peripheral the fovea complementing BCVA.

We had mentioned the evidence that changes in peripheral macula prognostically useful in the subsequent paragraph.

“We confirm our previous reports of a peripheral sensitivity loss as a potential surrogate marker of progression in DMO (1). Figures showing 128 sets of OCT, OFA and Matrix are available as Supplementary material for that paper (1).”

3. The authors should explain why their findings make a different for ophthalmologist

around the world and for the readers of *Plos one*

Current clinical practice guidelines for the management of DME is guided by anatomical evidence that occurs in late disease. In early disease, functional measures may help identify neuropathy of the retina. This study advances the concept of functional lesions before classical diabetic retinopathy develops and treatments may be developed to target functional lesions and may be an approach to circumvent structural lesions. We have now added the following statement in the Discussion:-

These findings support a shift in assessment guidelines to include functional measures outside the fovea before classical diabetic retinopathy develops and may lead to management strategies that circumvent structural lesions developing.

4. The authors should explain the source of the information and what were the criteria they used for adding to the paper Were the assessors masked? What was the ICC between them in orden to analyzed the data ? I Was the randomization digitalized?

As mentioned in the Methods the inclusion criteria were:-

“The inclusion criteria were LogMAR visual acuity < 0.3 and evidence of mild DMO, defined as retinal thickness ≥320 µm on spectral domain optical coherence tomography (OCT, Spectralis, Heidelberg Engineering, Germany) within the boundaries of the macula but not at the centre.”

Thus, in this study we did not have a bank of clinical judgments that needed to be made about the participants which would require examination of agreement between assessors. Instead, the two clinicians had to agree that the inclusion criteria were met, i.e. the ICC between them was 1.0. We were remis in not mentioning this and do so now in that section saying.

The two primary clinicians, FS and BBR, had to agree that the inclusion criteria were met.

5. Please add references and rephrase the sentence. English grammar should be applied.

“management protocol recommended by their ophthalmologist. For this reason, persons with DMO were followed up somewhat more frequently. Given the DMO patients were getting more frequent  updates about their ocular health than in their normal care, it was possible that some would  spontaneously improve their glycaemic control with resulting partial resolution of their DMO. This was helpful in the sense that we could examine whether the Matrix and OFA functional data were correlated with either improved or worsening retinal thickness. “

Thank you for pointing this out. We have now amended the paragraph to read:

Therefore, patients with DMO were followed up more frequently and received updates of their ocular health more often than they otherwise may have received. The frequency of examination feedback may have motivated some of the participants to improve their glycaemic control contributing to the resolution of DMO. In this light, examining Matrix and OFA functional data correlations with any changes in retinal thickness would be possible.

6. please add in the introduction one line of previous papers published showing clinical capabilities that specifically localize and delineate pathology and ancillary test such and OCT in order to help physician and Retina specialist to properly diagnose the DME

We have now added the following sentence to the Introduction:

Such advances in localised measures of retinal function(17,18,20,21) may assist clinicians to identify early functional changes before the onset of new signs of DR(22). Patients with diabetes may also exhibit thinning of retinal nerve fibre layer(23,24), and other layers prior to the onset of clinical DR(25,26).

7. These papers should discussed briefly and be referenced in the discussion part .Reference these :

-Retina. 2019 Jan;39(1):44-51. doi: 10.1097/IAE.0000000000002196.

DEXAMETHASONE IMPLANT FOR DIABETIC MACULAR EDEMA IN NAIVE COMPARED WITH REFRACTORY EYES: The International Retina Group Real-Life 24-Month Multicenter Study. The IRGREL-DEX Study.

[Iglicki M](https://www.ncbi.nlm.nih.gov/pubmed/?term=Iglicki%20M%5BAuthor%5D&cauthor=true&cauthor_uid=29697589)1, [Busch C](https://www.ncbi.nlm.nih.gov/pubmed/?term=Busch%20C%5BAuthor%5D&cauthor=true&cauthor_uid=29697589)2, [Zur D](https://www.ncbi.nlm.nih.gov/pubmed/?term=Zur%20D%5BAuthor%5D&cauthor=true&cauthor_uid=29697589)3,4, [Okada M](https://www.ncbi.nlm.nih.gov/pubmed/?term=Okada%20M%5BAuthor%5D&cauthor=true&cauthor_uid=29697589)5, [Mariussi M](https://www.ncbi.nlm.nih.gov/pubmed/?term=Mariussi%20M%5BAuthor%5D&cauthor=true&cauthor_uid=29697589)6, [Chhablani JK](https://www.ncbi.nlm.nih.gov/pubmed/?term=Chhablani%20JK%5BAuthor%5D&cauthor=true&cauthor_uid=29697589)7, [Cebeci Z](https://www.ncbi.nlm.nih.gov/pubmed/?term=Cebeci%20Z%5BAuthor%5D&cauthor=true&cauthor_uid=29697589)8, [Fraser-Bell S](https://www.ncbi.nlm.nih.gov/pubmed/?term=Fraser-Bell%20S%5BAuthor%5D&cauthor=true&cauthor_uid=29697589)9, [Chaikitmongkol V](https://www.ncbi.nlm.nih.gov/pubmed/?term=Chaikitmongkol%20V%5BAuthor%5D&cauthor=true&cauthor_uid=29697589)10, [Couturier A](https://www.ncbi.nlm.nih.gov/pubmed/?term=Couturier%20A%5BAuthor%5D&cauthor=true&cauthor_uid=29697589)11, [Giancipoli E](https://www.ncbi.nlm.nih.gov/pubmed/?term=Giancipoli%20E%5BAuthor%5D&cauthor=true&cauthor_uid=29697589)12, [Lupidi M](https://www.ncbi.nlm.nih.gov/pubmed/?term=Lupidi%20M%5BAuthor%5D&cauthor=true&cauthor_uid=29697589)13, [Rodríguez-Valdés PJ](https://www.ncbi.nlm.nih.gov/pubmed/?term=Rodr%C3%ADguez-Vald%C3%A9s%20PJ%5BAuthor%5D&cauthor=true&cauthor_uid=29697589)14, [Rehak M](https://www.ncbi.nlm.nih.gov/pubmed/?term=Rehak%20M%5BAuthor%5D&cauthor=true&cauthor_uid=29697589)2, [Fung AT](https://www.ncbi.nlm.nih.gov/pubmed/?term=Fung%20AT%5BAuthor%5D&cauthor=true&cauthor_uid=29697589)15,16,17, [Goldstein M](https://www.ncbi.nlm.nih.gov/pubmed/?term=Goldstein%20M%5BAuthor%5D&cauthor=true&cauthor_uid=29697589)3,4, [Loewenstein A](https://www.ncbi.nlm.nih.gov/pubmed/?term=Loewenstein%20A%5BAuthor%5D&cauthor=true&cauthor_uid=29697589)3,4,17.

-Acta Diabetol. 2018 Jun;55(6):541-547. doi: 10.1007/s00592-018-1117-z. Epub 2018 Mar 1.

Progression of diabetic retinopathy severity after treatment with dexamethasone implant: a 24-month cohort study the 'DR-Pro-DEX Study'.

[Iglicki M](https://www.ncbi.nlm.nih.gov/pubmed/?term=Iglicki%20M%5BAuthor%5D&cauthor=true&cauthor_uid=29497837)1, [Zur D](https://www.ncbi.nlm.nih.gov/pubmed/?term=Zur%20D%5BAuthor%5D&cauthor=true&cauthor_uid=29497837)2,3, [Busch C](https://www.ncbi.nlm.nih.gov/pubmed/?term=Busch%20C%5BAuthor%5D&cauthor=true&cauthor_uid=29497837)4, [Okada M](https://www.ncbi.nlm.nih.gov/pubmed/?term=Okada%20M%5BAuthor%5D&cauthor=true&cauthor_uid=29497837)5, [Loewenstein A](https://www.ncbi.nlm.nih.gov/pubmed/?term=Loewenstein%20A%5BAuthor%5D&cauthor=true&cauthor_uid=29497837)2,3,6.

-Ophthalmologica. 2019;241(1):9-16. doi: 10.1159/000492132. Epub 2018 Nov 8.

Effectiveness and Safety of Intravitreal Dexamethasone Implant (Ozurdex) in Patients with Diabetic Macular Edema: A Real-World Experience.

[Mello Filho P](https://www.ncbi.nlm.nih.gov/pubmed/?term=Mello%20Filho%20P%5BAuthor%5D&cauthor=true&cauthor_uid=30408801)1, [Andrade G](https://www.ncbi.nlm.nih.gov/pubmed/?term=Andrade%20G%5BAuthor%5D&cauthor=true&cauthor_uid=30408801)2, [Maia A](https://www.ncbi.nlm.nih.gov/pubmed/?term=Maia%20A%5BAuthor%5D&cauthor=true&cauthor_uid=30408801)1, [Maia M](https://www.ncbi.nlm.nih.gov/pubmed/?term=Maia%20M%5BAuthor%5D&cauthor=true&cauthor_uid=30408801)1, [Biccas Neto L](https://www.ncbi.nlm.nih.gov/pubmed/?term=Biccas%20Neto%20L%5BAuthor%5D&cauthor=true&cauthor_uid=30408801)1, [Muralha Neto A](https://www.ncbi.nlm.nih.gov/pubmed/?term=Muralha%20Neto%20A%5BAuthor%5D&cauthor=true&cauthor_uid=30408801)3, [Moura Brasil O](https://www.ncbi.nlm.nih.gov/pubmed/?term=Moura%20Brasil%20O%5BAuthor%5D&cauthor=true&cauthor_uid=30408801)4, [Minelli E](https://www.ncbi.nlm.nih.gov/pubmed/?term=Minelli%20E%5BAuthor%5D&cauthor=true&cauthor_uid=30408801)5, [Dalloul C](https://www.ncbi.nlm.nih.gov/pubmed/?term=Dalloul%20C%5BAuthor%5D&cauthor=true&cauthor_uid=30408801)6, [Iglicki M](https://www.ncbi.nlm.nih.gov/pubmed/?term=Iglicki%20M%5BAuthor%5D&cauthor=true&cauthor_uid=30408801)7.

-Ophthalmic Res. 2019 May 2:1-6. doi: 10.1159/000499540. [Epub ahead of print]

The Role of Steroids in the Management of Diabetic Macular Edema.

[Zur D](https://www.ncbi.nlm.nih.gov/pubmed/?term=Zur%20D%5BAuthor%5D&cauthor=true&cauthor_uid=31048580)1, [Iglicki M](https://www.ncbi.nlm.nih.gov/pubmed/?term=Iglicki%20M%5BAuthor%5D&cauthor=true&cauthor_uid=31048580)2, [Loewenstein A](https://www.ncbi.nlm.nih.gov/pubmed/?term=Loewenstein%20A%5BAuthor%5D&cauthor=true&cauthor_uid=31048580)3.

-Acta Diabetol. 2019 Oct;56(10):1141-1147. doi: 10.1007/s00592-019-01357-y. Epub 2019 May 14.

TRActional DIabetic reTInal detachment surgery with co-adjuvant intravitreal dexamethasONe implant: the TRADITION STUDY.

-[Iglicki M](https://www.ncbi.nlm.nih.gov/pubmed/?term=Iglicki%20M%5BAuthor%5D&cauthor=true&cauthor_uid=31089929)1, [Zur D](https://www.ncbi.nlm.nih.gov/pubmed/?term=Zur%20D%5BAuthor%5D&cauthor=true&cauthor_uid=31089929)2,3, [Fung A](https://www.ncbi.nlm.nih.gov/pubmed/?term=Fung%20A%5BAuthor%5D&cauthor=true&cauthor_uid=31089929)4,5,6, [Gabrielle PH](https://www.ncbi.nlm.nih.gov/pubmed/?term=Gabrielle%20PH%5BAuthor%5D&cauthor=true&cauthor_uid=31089929)7, [Lupidi M](https://www.ncbi.nlm.nih.gov/pubmed/?term=Lupidi%20M%5BAuthor%5D&cauthor=true&cauthor_uid=31089929)8, [Santos R](https://www.ncbi.nlm.nih.gov/pubmed/?term=Santos%20R%5BAuthor%5D&cauthor=true&cauthor_uid=31089929)9, [Busch C](https://www.ncbi.nlm.nih.gov/pubmed/?term=Busch%20C%5BAuthor%5D&cauthor=true&cauthor_uid=31089929)10, [Rehak M](https://www.ncbi.nlm.nih.gov/pubmed/?term=Rehak%20M%5BAuthor%5D&cauthor=true&cauthor_uid=31089929)10, [Cebeci Z](https://www.ncbi.nlm.nih.gov/pubmed/?term=Cebeci%20Z%5BAuthor%5D&cauthor=true&cauthor_uid=31089929)11, [Charles M](https://www.ncbi.nlm.nih.gov/pubmed/?term=Charles%20M%5BAuthor%5D&cauthor=true&cauthor_uid=31089929)12, [Masarwa D](https://www.ncbi.nlm.nih.gov/pubmed/?term=Masarwa%20D%5BAuthor%5D&cauthor=true&cauthor_uid=31089929)2,3, [Schwarz S](https://www.ncbi.nlm.nih.gov/pubmed/?term=Schwarz%20S%5BAuthor%5D&cauthor=true&cauthor_uid=31089929)2,3, [Barak A](https://www.ncbi.nlm.nih.gov/pubmed/?term=Barak%20A%5BAuthor%5D&cauthor=true&cauthor_uid=31089929)2,3, [Loewenstein A](https://www.ncbi.nlm.nih.gov/pubmed/?term=Loewenstein%20A%5BAuthor%5D&cauthor=true&cauthor_uid=31089929)2,3,13; [International Retina Group (IRG)](https://www.ncbi.nlm.nih.gov/pubmed/?term=International%20Retina%20Group%20(IRG)%5BCorporate%20Author%5D).

-Acta Ophthalmol. 2019 Aug 17. doi: 10.1111/aos.14230. [Epub ahead of print]

Disorganization of retinal inner layers as a biomarker in patients with diabetic macular oedema treated with dexamethasone implant.

[Zur D](https://www.ncbi.nlm.nih.gov/pubmed/?term=Zur%20D%5BAuthor%5D&cauthor=true&cauthor_uid=31421028)1, [Iglicki M](https://www.ncbi.nlm.nih.gov/pubmed/?term=Iglicki%20M%5BAuthor%5D&cauthor=true&cauthor_uid=31421028)2, [Sala-Puigdollers A](https://www.ncbi.nlm.nih.gov/pubmed/?term=Sala-Puigdollers%20A%5BAuthor%5D&cauthor=true&cauthor_uid=31421028)3, [Chhablani J](https://www.ncbi.nlm.nih.gov/pubmed/?term=Chhablani%20J%5BAuthor%5D&cauthor=true&cauthor_uid=31421028)4, [Lupidi M](https://www.ncbi.nlm.nih.gov/pubmed/?term=Lupidi%20M%5BAuthor%5D&cauthor=true&cauthor_uid=31421028)5, [Fraser-Bell S](https://www.ncbi.nlm.nih.gov/pubmed/?term=Fraser-Bell%20S%5BAuthor%5D&cauthor=true&cauthor_uid=31421028)6, [Mendes TS](https://www.ncbi.nlm.nih.gov/pubmed/?term=Mendes%20TS%5BAuthor%5D&cauthor=true&cauthor_uid=31421028)7,8, [Chaikitmongkol V](https://www.ncbi.nlm.nih.gov/pubmed/?term=Chaikitmongkol%20V%5BAuthor%5D&cauthor=true&cauthor_uid=31421028)9, [Cebeci Z](https://www.ncbi.nlm.nih.gov/pubmed/?term=Cebeci%20Z%5BAuthor%5D&cauthor=true&cauthor_uid=31421028)10, [Dollberg D](https://www.ncbi.nlm.nih.gov/pubmed/?term=Dollberg%20D%5BAuthor%5D&cauthor=true&cauthor_uid=31421028)1, [Busch C](https://www.ncbi.nlm.nih.gov/pubmed/?term=Busch%20C%5BAuthor%5D&cauthor=true&cauthor_uid=31421028)11, [Invernizzi A](https://www.ncbi.nlm.nih.gov/pubmed/?term=Invernizzi%20A%5BAuthor%5D&cauthor=true&cauthor_uid=31421028)12,13, [Habot-Wilner Z](https://www.ncbi.nlm.nih.gov/pubmed/?term=Habot-Wilner%20Z%5BAuthor%5D&cauthor=true&cauthor_uid=31421028)1, [Loewenstein A](https://www.ncbi.nlm.nih.gov/pubmed/?term=Loewenstein%20A%5BAuthor%5D&cauthor=true&cauthor_uid=31421028)1,14; [International Retina Group (IRG)](https://www.ncbi.nlm.nih.gov/pubmed/?term=International%20Retina%20Group%20(IRG)%5BCorporate%20Author%5D).

Multicenter Study

Br J Ophthalmol.2020 May;104(5):666-671. doi: 10.1136/bjophthalmol-2019-314523. Epub 2019 Aug 7.

Outer retinal hyperreflective deposits (ORYD): a new OCT feature in naïve diabetic macular oedema after PPV with ILM peeling

-Ophthalmologica. 2019;241(1):9-16. doi: 10.1159/000492132. Epub 2018 Nov 8.Effectiveness and Safety of Intravitreal Dexamethasone Implant (Ozurdex) in Patients with Diabetic Macular Edema: A Real-World Experience.

Mello Filho P1, Andrade G2, Maia A1, Maia M1, Biccas Neto L1, Muralha Neto A3, Moura Brasil O4, Minelli E5, Dalloul C6, Iglicki M7.

-Ophthalmic Res. 2019 May 2:1-6. doi: 10.1159/000499540. [Epub ahead of print]

The Role of Steroids in the Management of Diabetic Macular Edema.

Zur D1, Iglicki M2, Loewenstein A3.

-Acta Diabetol. 2019 Oct;56(10):1141-1147. doi: 10.1007/s00592-019-01357-y. Epub 2019 May 14.

TRActional DIabetic reTInal detachment surgery with co-adjuvant intravitreal dexamethasONe implant: the TRADITION STUDY.

-Published online 2018 Jul 11. doi: 10.1371/journal.pone.0200365PMCID: PMC6040739PMID: 29995929 Biomarkers and predictors for functional and anatomic outcomes for small gauge pars plana vitrectomy and peeling of the internal limiting membrane in naïve diabetic macular edema: The VITAL Study

-Acta Diabetol2020 Apr 16. doi: 10.1007/s00592-020-01530-8. Online ahead of print.Results in comparison between 30 gauge ultrathin wall and 27 gauge needle in sutureless intraocular lens flanged technique in diabetic patients: 24-month follow-up study

Eye 2021 Aug 9. doi: 10.1038/s41433-021-01722-8. Online ahead of print.

Next-generation anti-VEGF agents for diabetic macular oedema

-Retina. 2019 Nov;39(11):2161-2166. doi: 10.1097/IAE.0000000000002270.

UNDERDIAGNOSED OPTIC DISK PIT MACULOPATHY: Spectral Domain Optical Coherence Tomography Features For Accurate Diagnosis

Matias Iglicki 1 , Catharina Busch 2 , Anat Loewenstein 3	4	5 , Adrian T Fung 6	7	8 , Alessandro Invernizzi 8	9 , Miriana Mariussi 10 , Romina Arias 1 , Pierre-Henry Gabrielle 11 , Zafer Cebeci 12 , Mali Okada 13 , Jerzy Nawrocki 14	15 , Zofia Michalewska 14	15 , Michaella Goldstein 3	4 , Adiel Barak 3	4 , Dinah Zur 3

-EYE-2021 Apr;35(4):1111-1116. doi: 10.1038/s41433-020-01309-9. Epub 2021 Jan 11.

-Ophthalmology.2018 Feb;125(2):267-275. doi: 10.1016/j.ophtha.2017.08.031. Epub 2017 Sep 19.

OCT Biomarkers as Functional Outcome Predictors in Diabetic Macular Edema Treated with Dexamethasone Implant

Eye 2022 Jan 18. doi: 10.1038/s41433-022-01931-9. Online ahead of print.

Vitrectomized vs non-vitrectomized eyes in DEX implant treatment for DMO-Is there any difference? the VITDEX study

-EYE 2022 Aug 29. doi: 10.1038/s41433-022-02222-z. Online ahead of print.

Naïve subretinal haemorrhage due to neovascular age-related macular degeneration. pneumatic displacement, subretinal air, and tissue plasminogen activator: subretinal vs intravitreal aflibercept-the native study

- Ophthalmol Retina.2021 Nov;5(11):1097-1106.

doi: 10.1016/j.oret.2021.01.013. Epub 2021 Feb 1.Detection of Diabetic Retinopathy from Ultra-Widefield Scanning Laser Ophthalmoscope Images: A Multicenter Deep Learning Analysis

Following the sections on the utility of assessing peripheral visual function in DMO and DR in the discussion were have cited 9 of these papers added the following:-

One of the motivations for this study is the desire to understand when to treat macular oedema given the growing number of effective treatments including dexamethasone (52-57), other steroids, (58) and next generation anti-VEGFs (59). Further studies on when to treat early-stage DMO with some of these may be aided by information on peripheral visual function.

8.please add how, and how long takes for a retina specialist to switch in their mind the mandatory use of Perimetry what position in the current algorithm of understanding, diagnosis and treatment of DME has or will have this technique

We have now added the following paragraph to the Discussion:-

In the current clinical guidelines, perimetry is not used in the evaluation of DMO. This may be due to time factor; however, visual acuity is the only functional measure. Newer OFA protocols are shorter in duration and can measure retinal functional more broadly(2). Considering the value of functional lesions that cannot be measured by imaging or clinical examination, changing to functional measures my be advantageous.

9. Results could be misinterpreted add a short summary of the similarities in different OCT devices and also add diferente oct modalities SD, swept source EDI etc and what can be improved in the process of detecting RAP is mandatory in the discussion section

We agree that the current study was limited by using just one spectral domain OCT to characterise structural changes. We have now added to the Discussion just before the limitations section:-

This study took OCT thickness measurements to be the best indicator of DMO. We used one only one spectral domain device. It is possible that other types of OCT may yield a more accurate assessment such as swept source OCT, or complementary information about the choroid using enhanced depth imaging (EDI). We have recently reported on macular OCT-angiography in young Type 1 diabetics (60) and compared that to an OFA method that tests both eyes in <90 seconds (61). The rapid OFA test is ideal for children. Future studies of early-stage diabetic eye disease and OFA need to consider comparisons with newer OCT modalities.

9. Please apply correction for misspelling and English grammar

We have made these corrections thank you.

10 . This paper should be corrected by an English redactor

Thank you for the suggestion. All the authors are native speakers of English. That does not mean that our writing style is perfect, however. Accordingly we have had several senior authors involved in re-reading the revised version.

**Reviewer #2:**

This study by Sabeti et al presents interesting research findings regarding the comparison of structure function correlation in subjects with DMO. While findings are useful, it is not presented in a very readable manner. Many places assumes that reader would understand, especially the figures or regarding the variation in slopes. More details at specific locations have been requested. The presentation of data is really neat and unique. However, authors make further attempt to clarify more information in the figure legend. Important points have been made in bold.

Abstract:

Please include 1 sentence rationale for the study in the Purpose section.

We have now added the following statement to the Purpose:-

Retinal function beyond foveal vision is not routinely examined in the clinical screening and management of diabetic retinopathy although growing evidence suggests it may precede structural changes. In this study we compare optical coherence tomography (OCT) based macular structure with function measured objectively with the ObjectiveFIELD Analyzer (OFA), and with Matrix perimetry. We did that longitudinally in Type 2 diabetes (T2D) patients with mild Diabetic Macular Oedema (DMO) and good vision to evaluate changes in retinal function more peripherally over the natural course of retinopathy.

Methods

What is the time interval between visits.

This study measured changes in retinal structure and function as part of their routine standard of clinical care by their managing ophthalmologist. Therefore, as described in the Methods line 69, there were a “median of 3 visits over 457 ± 198 days (mean ± SD)”. To make the interval between visits clearer in the methods we have now added that:-

with a median interval of 243.5 ± 145.6 days (mean ± SD) between visits.

What is mfPOP variants?

As described in the methods 2 mfPOP variants were used in this study with the primary difference being the visual eccentricity that was sampled. To make this clearer in the text we have added the following sentence to the methods:-

The primary difference between the two stimulus variants were that Tthe stimuli were scaled with eccentricity and subtended either ±15° or ± 30° from fixation with a maximum luminance of 288 cd/m^2^ presented on a 10 cd/m^2^ dim yellow starburst background with a small central fixation cross.

Results

The reduction in retinal thickness seem very small. How is that deemed significant. Which test was used?

Multivariant linear regression was used to evaluate the change in thickness overtime for each region and this was stated in the Results on line 210. We have now added the following statement at line 166 to the Methods to make the analysis clearer:=

We used multiple regression-linear models to examine the changes in retinal thickness , and changes in sensitivity and response delay overtime (see below).. Thus, significant change was quantified by slopes with p-values < 0.05.

What delay are the authors referring to ? It is arbitrary. Numbers should be provided for reduction in retinal thicknesses over time.

In terms of the Response delays these refer to the time from stimulus onset to peak pupil contraction. To make this clearer in the text we have added the following statement to the methods:-

Response delays were represented by time from stimulus onset to peak contraction.

The 128 numbers for the significant reduction, or increase, in thickness over time are presented in the right-most 2 columns of Figure 5. That is each coloured check represent a region at which there was a significant change in thickness. The calibration bar at the bottom of the two columns give the magnitude and sign of the slopes. It would be difficult to tabulate the 128 numbers and indicate where they were in the visual field. By comparison Matrix perimetry identified 7 regions.

“Matrix perimetry quantified many fewer regions that changed significantly.” Need to more specific.

How are conclusions supported by the results? Without providing the result in the abstract and claiming the conclusion does not seem appropriate.

This statement is in the Abstract, so it is difficult to provide much explanation. However, we have added words to indicate significant change over the 27 months of the study. Therefore, the last sentence of the Abstract Results section now reads.

‘Matrix perimetry quantified many fewer regions that changed significantly over the 27 months.’

Introduction

Line 3 DR is also functional manifestation?

Agreed, DR represents functional and structural change, therefore we have modified the text on line 3 to read:-

Diabetic retinopathy, a structural and functional manifestation of proinflammatory activity within the retina in chronic hyperglycemia,

Line 7 Need to qualify what is fenofibrate?

Thank you for pointing this out we have now added to the text on line 7 to read:

Possibilities for treatment with fenofibrate, a peroxisome proliferator activated receptor alpha agonist, used primarily to treat dyslipidaemia and high-density lipoprotein cholesterol or other interventions are also emerging.

What is mild “off-centre” DMO?

We have defined mild “off-centre” DMO on line 69 of the methods.

and evidence of mild off-centre DMO, defined as retinal thickness ≥320 µm,

Lines 14-16 limitations of erg and perimetry should be qualified respectively not grouped together as done presently.

Thank you for bringing this to our attention. We have now qualified the limitations of each functional measure in the introduction:-

Whilst these functional measures have shown promise, electroretinography suffers from long setup times (15) and poor repeatability due to the subjective nature of testing and small stimulus sizes in standard automated perimetry(16)

Authors need to justify why not mf ERG which is also objective and gives focal responses? Why mfPOP over mf ERG?

We have now added the following sentence to the introduction:-

Thus, 4 visual field maps are generated in under 7 minutes and may be an advantage over other forms of objective perimetry, such as multifocal electroretinography.

Line 19-21 ; sentence construction needs to be addressed.

Addressed.

Lines 26-29 : how is it relevant in the present context.

Agreement between visual evoked potentials and pupillography on the same patients, measured on the same day, confirms and strengthens the evidence of the impact of diabetic neuropathy on the visual system. To convey this in the text we have added the following statement:-

This confirms and builds evidence of the impact of diabetic neuropathy on the visual system at various stages of DR.

The introduction is not flowing well. The exact rationale for the study not coming. Connecting sentences are missing. There are three separate chunks of paragraphs.

Thank you for identifying this weakness in the introduction. We have now added the following text in the introduction to link the previous work from our lab and other groups that has led to the purpose of our investigation.

Although structural retinal changes evaluated by stereoscopic ophthalmoscopy remains the mainstay clinical assessment, managing DR becomes a reactionary procedure. One approach, which may circumvent progression to sight threatening forms of DR would be to identify early localisd changes in the retina. We believe that changes in retinal function may represent the earliest signs of distress in the retina. The purpose of this study was to evaluate the ability of objective and subjective perimetry to detect longitudinal change in the natural course of DMO.

Lines 34-40: not suited for this context. Mostly methods related information in the introduction does not fit well.

To address this comment we have now amended the final paragraph in the introduction to read:-

We enrolled T2D patients with mild DMO, and similar T2D patients without DMO at baseline, and followed them longitudinally for 1.5 years. Given the presence of DMO our primary measure was macular retinal thickness as measured by Optical Coherence Tomography (OCT), and we did regression analyses to identify significant changes in regional thickness over the study period. Secondary measures were similar regression analyses of functional changes measured by Matrix per-region sensitivities, and OFA per-region sensitivities and delays. We also sought to examine the correlation between regional structural and functional changes and mapped the OCT, Matrix and OFA central 30° data (sensitives and delays) to a common 44-region grid. Based on previous findings of retinal dysfunction early in DR, we hypothesize that the presence of peripheral changes in retinal function is associated with patients with off-centre-DMO and good vision. We seek to evaluate these changes utilising objective and subjective visual sensitivity measures to aid our understanding of the impact mild DMO may have on retinal function, thus guiding clinical monitoring and management of this condition.

40-42 :poorly written hypothesis; need to state why you are expecting something.

Addressed. We have now added the following statement to the concluding remarks of the introduction:-

Based on previous findings of retinal dysfunction early in DR, we hypothesize that the presence of peripheral changes in retinal function is associated with patients with off-centre DMO and good vision.

**METHODS;**

47-48: not agreeing with statement that both eyes had DMO

More clear information on the how the subjects were recruited needs to be provided.

In fact, we did not say that the patients had DMO in both eyes, quite the opposite. In the first sentence of the Methods we indicated that of the 26 diabetes patients were recruited 10 of whom had mild DMO in at least 1 eye. Table 1 also shows the 6 non-DMO subjects and 10 DMO subjects at baseline. Figure 3 similarly splits the subjects. Some of the eyes that were initially without DMO developed it over the study period. This was mentioned in respect of the bottom row of Figure 5 , now at lines 220 to 223 of the Results

‘Patient mf87171 (row I), a T2D patient without initial DMO showed significant retinal thickening towards the end of the study period (I^3^, green) that aligned with reduced OFA sensitivity (I^0^) slopes and shorter response delays (I^1^, blue). ‘

We have now amended the methods to read:-

Subjects were identified following their DR screening by their attending clinician from private ophthalmology (RCB) and optometry (FS) practices in Canberra. Patients were then written to and invited to participate in the study.

The issue may have arisen from the first sentence of the abstract which has been correct to read

‘Both eyes of 16 T2D patients (65.0 ± 10.1, 10 females), 10 with baseline DMO, were followed for up to 27 months providing 94 data sets.’

Lines59-60: Exclusion criteria were absence of XXX conditions seem to suggest the opposite. Please check

Addressed. The criteria are accurate.

Line 62: A set protocol was not followed for each of the patients in terms of the follow-up?

That is correct, this was a convenience sample that followed the standard clinical management by their attending ophthalmologist over time. As such, patients were not followed-up at specific intervals but rather at the scheduled follow-up examinations. This has been conveyed in the text on line 62-67.

Line 108 : what is “significantly faster or slower than normal”?

To make this statement clearer we have now amended the statement to read:-

Where per-region time to peak contraction can be significantly faster or slower than normal in the same eye.

Line 138: Expand further on how AmpStd μm to decibel sensitivity (dB)?

Sorry, this method has been given in detail elsewhere, but we forgot to reference that paper. We do now and so the sentence a line 157 now reads

For regression analysis we transformed the AmpStd µm to decibel sensitivity (dB) using a generalised log-transform (32), which also stabilized the variance.

Line 191: What was defined as “significant change”?

We have now amended the line to read:-

The longitudinal data over time were fitted by linear regression and those regions with significant change in retinal sensitivity or thickness over time between baseline and final visit are coloured.

**Correlation heat map of OFA sensitivity changes with OCT thickness changes may be done as done in reference 25**

Yes, we agree and so we have used exactly the method of reference 25 (now ref 34) to create the first four rows of Figure 4 and all of the plots in Figure 5.

**Lines 215 -225:** Which points had significant slopes? This section is not clear with respect to the figure. Please expand further.

The checks in Figure 5 that are coloured. The magnitude and sign of the significant slopes is given by the calibration bars at the bottom of each column. In answer to a previous question we have added the text

The longitudinal data over time were fitted by linear regression and those regions with significant change in retinal sensitivity or thickness over time between baseline and final visit are coloured. The colour-map legends at the bottom of the pairs of columns indicate the magnitude and sign of those per-region slopes.

Line 268-270: “Long-term follow-up of Matrix sensitivity in our cohort showed little significant change over time and did not reflect changes in retinal structure”

Even the OFA sensitivity did not show point-by-point correspondence with change in retinal thicknesses?

What is the implication of delay observed in pupil response?

Please clarify

We had mentioned this in the Methods. We have added the word delay to that text to make it clearer

‘Pupil responses were measured as real-time pupil diameter, relative to the population mean pupil diameter of 3.5 mm. In addition, pupil neuropathy would produce a generalised depression of sensitivity or global delay in responses, unlike the focal per-region changes found in our studies of type 2 diabetes(17,30), where per-region time to peak contraction delay can be significantly faster or slower than normal in the same eye (30).’

Thus, the delay at each region indicates aspects of the physiology of that patch of retina.

Also hypothesized in the Introduction, we believe that there may be signs of distress in the retina represented by functional changes measured more peripherally. Our findings suggest that changes in OFA sensitivity and response delays were more informative than retinal sensitivity measured by Matrix perimetry. This was previously articulated in the Discussion on line 289-292.

“In automated perimetry measuring a sensitivity increase requires near super-human contrast sensitivity, whereas for OFA only an increase in a large objective response is required”

Changed to

‘only a small increase in a large objective response is required.’

Line 276 It is Feigl not Feigle

Addressed.

Why only 10 subjects data presented instead of 16?

We are not quite sure what you are referring to here. There were only 10 subjects presenting with DMO at baseline. But in terms of reported data in the results there is no case of data for 10 subjects being shown. Figures 5 and 6 present data for the 9 subjects who showed significant change over the study period. As mentioned in the text the bottom row was for a person who initial did not have DMO but developed it over the course of the study, subject mf87171.

**Comments on Figures**

**Fig 1**

What are the dashed lines? What does it mean?

Both the dashed and solid contours represent the borders of the lozenge shaped stimuli. The dashed lines allow the degree of potential spatial overlap of the stimuli to be understood. We have now added this in the legend for Figure 1. As mentioned in the legend the pseudo-random presentation means no pairs of overlapping stimuli are ever presented.

Can the rings be clearly demarcated?

Not clear about overlap of stimuli. Please explain clearly.

We have added the following to the legend of figure 1

Both P30° and P60° consisted of 5 rings of lozenge-shaped stimuli. Going from central to outer the borders of the rings are dashed (rings 1,3,5) or solid (rings 2,4).

In theory, if stimuli are all presented simultaneously they would overlap in space but his never happened. Rather, they were presented in a random sequence and adjustments to stimulus brightness were applied across regions to balance response sizes from each stimulus region. This is explained in the legend in Figure 1. We now referenced this in the Methods lines 92-96.

Panels B and D shows the two halves in each panel are in unequal size. Why is that?

This represents the one half of all overlapping regions turned on for both the wide eccentricity (±60°) and macular (±15°) stimulus array.

We have now amended the legend to read:-

(B,D) show half the left eye stimuli turned-on to illustrate the luminance-balancing method that adjusts response sizes from normal subjects to be more even across the examined field.

And later

The two halves show rings 1,3,5 on the left and 2,4 on the right. There are thus 11 stimuli per quadrant.

“Reducing the luminance in regions with inherently higher sensitivity enhances the responses in less-responsive regions”

This is not clear and does not belong in the Figure legend.

Agreed. This statement was explained in the Methods and therefore was removed from the Figure legend.

Mention that there are 11 per each quadrant.

Addressed, see above.

**Fig 2**

8X8 refers to mm?

This refers to the number squares imaged in the posterior pole scans (30° horizontally x 25° vertically). We have added this to the text on line 137

Why are the blue panels in panel A tilted?

The blue dashed panels are tilted because the default Heidelberg posterior pole scans imaged with a 7-degree tilt to align with the fovea to optics disc. We have now added this on line 138.

**IMPORTANT: The regions where there is overlap of more than one box; how is the corresponding OCT thickness/OFA parameters considered?**

We applied weights to each segment that was overlapping between 10-2 Matrix stimulus array and OFA and OCT maps. We have described this in the methods and refer to a previous published paper detailing this method on line 169-173.

Fig 3

Abstract says 16 patients with baseline DMO but the table 1 and Fig 3 says non-DMO subjects/ Needs fixing

Sorry the sentence of the abstract has now been correct to read:-

Both eyes of 16 T2D patients (65.0 ± 10.1, 10 females), 10 with baseline DMO, were followed for up to 27 months providing 94 data sets.

**Fig 4**

Why this subject data was chosen to present?

This subject was selected because they represented a clear example of corresponding changes between retinal structure and function over time. We have now added the following sentence to the results:-

This subject was selected due to the clear corresponding changes between retinal thickness and function changes over time.

Difference map between Visit 4 and Visit 1 may be more useful. Visually, it is difficult to compare so many individual points across the visits.

Well, as shown by Figure 3 only 4 subjects had 4 visits, so that would not leave much data.

What is meant by “mapped to the 10-2 format”

Here we are presenting the weighted OFA sensitivity and response delay per-region data corresponding to the 10-2 stimulus positions. In response to reviewer 1 we modified the text in the introduction to explain the mapping process further on line 48-50.

What does the square inside represent.? In each of the panels?

The inner square represents the higher spatial sampling of the central visual field with the Matrix perimeter.

What is on the x axis for panels a to d and g to j?

The x-axis presents the degrees of visual eccentricity along the horizontal meridian in similar scale to the y-axis. To make this clearer in the text we added a short statement to the figure legend.

What does Y axis on the left indicate? – 5 and 5 ?

Addressed in the response to the preceding comment above and added to the figure 4 legend.

I would keep both visits next to each other for each eye separately for easy comparison?

We suppose there are merits to both ways of doing it. That is one can keep the visit data together or the eyes. This way it is clearer that for OFA Sensitivity both OS and OD improved on the second visit for both P30° and P60°. The other presentation method might be better for the OCT data.

Much the same applies to the eyes. In neuro-ophthalmology when data from a pair of eyes is presented they generally put OS on the left, but in generally ophthalmology od is presented on the left. So, if we grouped the visits as pairs of columns, which would be best on the left os or od?

Line 163 says day 517 and figure says 571

Thank you for pointing this out, addressed.

**Fig 5**

Which slopes are the authors referring to ?

Significant slopes are highlighted in the figure regions by colour. Regions that are not coloured represent non-significant regions. To make this clearer in the text, I have added the following statement to the legend of Figure 5:-

Data for subjects with significant slopes (regional progression or recovery colour coded, non-significant regions white)

OCT “Thickness” it should be?

Addressed

What about the remaining 6 subjects?

Figures 5 and 6 present data from 9 of the 16 subjects, not 10. Subject shown are those who had significant changes over time. This information was edited out of the text but was mentioned in the legend. We now modified the first reference to Figure 5 in the text to read.

Figure 5 shows data from the 9 subjects who showed significant (p < 0.05) longitudinal changes (slopes) in per-region retinal function…

What is the aim of this figure?

To demonstrate the regions showing significant changes across all functional and structural measures of a sample of participants. We have added to the text in the results section (line 218-224) in response to a comment from reviewer 2 above and believe that this has now been addressed.

Authors should aim to highlight if retinal thickness and OFA sensitivity region is corresponding to the same points?

We have pointed the correspondence between retinal thickness and OFA sensitivity on lines 213 – 215.

Fig 5. In heat map guide what does white mean? No change; it is clearly seen in guide in Fig 6 but not in Fig 5. Please change.

Addressed, please see response to Review 2 Figure 5 legend above.

But the heat map does not seem to indicate that?

It does actually

Why matrix perimetry seems largely unchanged but the retinal thickness changed noticeably in at least 3 subjects?

Commentary on this result was raised in the Discussion on line 300 – 305. Basically, Matrix perimetry does not seem to work that well, possibly due to being a subjective method with significant test-retest variability.

**Responses to Editorial Office Comments**

A. If there are ethical or legal restrictions on sharing a de-identified data set, please explain them in detail (e.g., data contain potentially identifying or sensitive patient information, data are owned by a third-party organization, etc.)

In terms of the human ethics committee, deidentification is always difficult. For example, a person’s age, sex, and disease diagnosis and stage, years of diabetes, eyeglass use and prescription, prescription medication, past eye surgery etc can be used to identify people with reasonable accuracy. We would need to seek permission from the Ethics committee to disclose the data.

B. Please also name the institution who has imposed these restrictions upon your data (e.g., a Research Ethics Committee or Institutional Review Board, etc.).

The ANU Ethics Committee, local Health Department’s Human Ethics Committee and ANU commercialisation have imposed this restriction on the dataset being shared.

C. Please note that it is not acceptable for authors of the study to be either the sole listed point of contact for data or the ultimate provider of data that is made available upon request. At this time, please provide direct, non-author contact information (preferably email) for the body imposing the restrictions upon the data, to which data access requests can be sent.

The ANU commercialisation office, would need to approve the data to be shared. They can be reached on [commercialisation@anu.edu.au](mailto:commercialisation@anu.edu.au)

We could provide all that data publicly, with the exception of some data that produced Table 1, the Patient Demographics, given the issue of deidentification. Taking these factors into account we would like to add the following statement to the manuscript

“The authors have made all the data that was analysed to make the Figures and Table 1 available in a public data base. The data underlying the demographics data of Table 1 have been partially redacted in order to de-identify the data.”
