# Supplementary material for: Objective perimetry identifies regional functional progression and recovery in mild Diabetic Macular Oedema
Source: PLoS One. 2023 Jun 15;18(6):e0287319. doi: 10.1371/journal.pone.0287319 (PMC10270604; doi:10.1371/journal.pone.0287319)
Supplement: Attachment — Submitted filename: Response to Reviewers_v4.docx [file pone.0287319.s007.docx]

**RESPONSE TO REVIEWER’S COMMENTS**

Reviewer Comments:

**Reviewer #1:** All comments have been addressed.

**Reviewer #2:**

Many places in the revised manuscript newly added text are not highlighted in red (for instance line 7-10 and line 37-40) and ideally where does the new text sit in the revised manuscript (in terms of line numbers) needs to be provided. It makes it difficult to identify and track the changes for the reviewer! Authors have misunderstood some of the abstract-related comments and addressed as if it were directed at the body of the paper. Please correct accordingly.
There are still methodological details missing. The results can be improved in terms of ease of reading.

Thank you for pointing this out. We apologise that we did not rigorously check that the additions were highlighted prior to our last revised submission. We have now reviewed the amendments following the reviewer’s comments and correctly identified them in RED if original and GREEN if secondary revisions apply for this round.

1. Consider rewriting the below sentence
“Subjects were assessed at a median of 3 visits over 457 ± 198 days (mean ± SD) 75 with a median interval of 243.5 ± 145.6 (median ± SD) between visits”
Median is usually supplied with IQR. Mean and median used interchangeably. Also mention in the abstract.
“We enrolled T2D patients with mild DMO, and similar T2D patients without DMO at baseline, and
followed them longitudinally for 1.5 years.”
A version of this statement needs to be in abstract methods

We have now amended the abstract to read:

“We did that longitudinally in Type 2 diabetes (T2D) patients with mild Diabetic Macular Oedema (DMO) with good vision and a similar number of T2D patients without DMO”

2. Expand mfpop and OFA in abstract methods

Addressed, thank you.

3. “Matrix perimetry quantified many fewer regions that changed significantly”

Which regions need to be mentioned at least grossly

We have now added the following statement to the Abstract:

“mostly presenting in the central 8 degrees.”

4. Authors need to justify why not mf ERG which is also objective and gives focal responses?
Why mfPOP over mf ERG? The justification is not sufficient. Is any distinct advantage?

Thank you for pointing this out. We have now added the following statement to the introduction

“In addition, OFA requires minimal training to administer with rapid setup times.”

5. Line 50 need to provide references for previous findings

Addressed, thank you.

6. P 30 and P 60 which one is subtending 15 and 30 degrees? Please clarify.

We have now added the following statement to the Methods:

“We refer to the two variants as P30° and P60° pertaining to the ±15° and ± 30° stimulus array, respectively”

7. “many fewer” in the abstract results sounds paradoxical. Please change.

We have now amended the text in the abstract.

Lines 109-110
8. “Total stimulus duration was 6 minutes divided into 9 segments of 40s with short breaks between segments”
Assuming short break is 10 seconds; close to additional 1 min and 20 seconds is added to the protocol. Therefore the total testing time should be mentioned as 7 to 8 minutes.

Corrected, thank you for pointing this out.

Methods:
Important points

Spatial extent of DMO (in degrees) in each of the patients’ needs to be quantified at baseline and the follow up visit. Preferably as table.

OFF centre DMO – how much was it off? How many degrees away from the macula? Need to be quantified.

This has direct implications on the structure-function mapping. That can help in understand how some points recover and not others.

Fig 4 suggestion

Keep OD baseline and follow up together instead of OD and OS together. Applies to OS as well,

We agree the suggested presentation method has some merit scientifically. As mentioned before the current presentation method conforms to standard ophthalmic practice for OU data sets with OD on the left, as the patient presents to the clinician. Fig 4 is presented to illustrate the format of the data more than to quantify anything. In particular, the current format sets up understanding of the format of Figure 5, which presents the significant slopes in OD/OS format. Flipping the columns to baseline/follow-up would mean that feature of the flow of the figures, and so too understanding, would be lost. Quantification is done for all the data Figures 5 and 6 (also in OD/OS format).

Another nice feature of the original Fig. 4 is that it allows changes to be seen by free-fusion of the pairs of baseline and follow-up columns as if they are binocular pairs. This makes it immediately obvious what is different. We now mention this in the Figure legend saying

“Readers may wish to free-fuse A and B”

That is also true of all 47 data sets in Supplementary Figure 1. This feature is lost if the columns are swapped as suggested. That being said the idea has merit, therefore we have done as suggested in Supplementary Figure 2. At the end of the paragraph on Figure 4 we now say

“The same data is presented with the baseline data for both eyes on the left in Supplementary Figure 2.”

If the DMO is well restricted within the macula, why are the peripheral points showing improvement or deterioration? In Fig 5 and 6

We have postulated on the origin of changes in retinal function in the discussion (lines 308 – 318). We now also add that structural changes may have been due to the frequency of clinical review and advice from the attending physician for diet and lifestyle modification. We have now added the following sentence on line 286 of the discussion:

“Conversely, eyes that reduced in retinal thickness possibly due to the greater frequency of follow-up and advice, improved in response amplitude, with response delays seeming to be less able to recover from former DMO. “  

Fig 4 legend says it is Diabetic patient but the patient is DMO patient. Needs to be changed

Addressed, thank you.

Fig 5
Each parameter increase or decrease may mean deterioration or recovery
Increase in sensitivity is improvement but increase in thickness is perhaps worsening

So one suggestion is to add alphabet R (recovery) or W (worsening) at either end of the legend for each variable to help the reader. In fact letter choice is up to the authors

Difference is taken baseline – last visit or vice versa?. This information needs to be present in both Fig 5 and 6 legend.

We did not look at change as the difference between the first and last visit. Firstly, that would throw away most of the data from the many intermediate visits, which are shown in Figure 3. Another reason for not taking the difference between the first and last visit is that the duration of time between those visits is different for every subject (Fig. 3), so the differences would not be calibrated for a uniform time period. To resolve both issues we fitted a line to the data to compute the slopes in dB/year, ms/year and um/year. That process also meant we could understand if the slopes were significant or not. The colour-coded slopes are reported with those units at the bottom of Figures 5 and 6. This was made clearer by amending the last sentence of the Methods which now reads

“We used multiple regression-linear models to examine the changes in retinal thickness, and changes in sensitivity and response delay overtime (see below). Thus, significant change was quantified by slopes with p-values < 0.05.”

The word slope then appears 8 more times in the text and there is no mentioned of computing the difference between the first and last visits. The text for Figure 5 says

“The longitudinal data over time were fitted by linear regression and those regions with significant change in retinal sensitivity or thickness over time between baseline and final visit are coloured. The colour-map legends at the bottom of the pairs of columns indicate the magnitude and sign of those per-region slopes.”

The main point about the observed slopes for OFA is that they appear to follow changes in retinal thickness, whether retinal thickness improved or got worse. This is detailed on page 16.

“Here, the presence of sensitivity loss and shorter response delays beyond 4° from fixation, identified eyes that increased in retinal thickness overtime (Fig. 5). Conversely, eyes that reduced in retinal thickness possibly due to the greater frequency of follow-up and advice, improved in response amplitude, with response delays seeming to be less able to recover from former DMO. The results perhaps indicate that therapeutic interventions might provide recovery of sensitivity.”

The reviewer’s comments in green helped clarify this. Thank you.

Discussion point

What are the implications of delays?

What mechanisms is responsible for the delays? Can you point to me if it has been already mentioned explicitly in the paper?

We hypothesize that response delays measured by OFA may represent neural impairment from both local retinal inflammation or higher cortical neuropathy. We have added the following sentence in the discussion:

“Therefore, response delays may represent changes in physiology locally at the retinal level or from higher cortical centres.“

It is important to note that the observed changes are not due to iris neuropathy which would create global shifts of delay that were the same for every region. Instead, we see in the 47 data sets of Supplementary Figure 1 many cases where slower and faster than normal delays occur in the same retina. Therefore, it appears that these are local functional defects. The fact that they are afferent defects from the retina was demonstrated by reference 17 (n.b. its title).

So, we have now added to the above at line 317

“Evidence that they are afferent defects from the retina has been provided (17).”

We thank the reviewer for allowing us to make these valuable points.
