# Supplementary material for: Objective perimetry identifies regional functional progression and recovery in mild Diabetic Macular Oedema
Source: PLoS One. 2023 Jun 15;18(6):e0287319. doi: 10.1371/journal.pone.0287319 (PMC10270604; doi:10.1371/journal.pone.0287319)
Supplement: Attachment — Submitted filename: Response to Reviewers_v6_final.docx [file pone.0287319.s008.docx]

**RESPONSE TO REVIEWER’S COMMENTS**

Reviewer Comments:

Reviewer #2: Other comments have been addressed. However, these comments are have not been addressed.

Spatial extent of DMO (in degrees) in each of the patients’ needs to be quantified at baseline and the follow up visit. Preferably as table.

OFF centre DMO – how much was it off? How many degrees away from the macula? Need to be quantified.

This has direct implications on the structure-function mapping. That can help in understand how some points recover and not others.

Thank you for this comment we agree this type of information could potentially add to understanding in terms of structure function mapping and what is going on with recovery and progression. We therefore measured the distribution of DMO in each eye quantifying:-

1) The area of the main oedematous region
2) The x,y coordinates of the centroid of that region in each eye

This was done for the first and last visit of each from those subjects who showed a significant change in DMO over the study in at least one eye, i.e. those subjects of Figure 5. That provided 36 eyes (2 visits * 18 eyes) for the analysis. We used the OCT 8×8 thickness grid (Fig. 2A) data from those eyes. Before analysis we flipped the data from right eyes to match left eyes. We then formed an 8×8 normative template by taking the median at each of the 8×8 locations for the 36 eyes. The template was thus the typical 8×8 thickness profile with outliers removed. The template was then subtracted from each 8×8 data set. Regions of the retinas that were thicker than normal were considered oedematous. We used the *regionprops* function of Matlab to identify the areas of oedema from each retina and found: 1) the area of the largest single region/eye, 2) its Equivalent Radius, and the x,y coordinates of its Centroid. We agree with the reviewer that it is sensible to project the data into visual space to match Figure 5. The new Table 3 represents the requested data.

The steps of the analysis behind Table 3 and some summary statistics are provided in the new Figure 6. The old Figure 6 is now Figure 7. Figure 6 and its legend and the accompanying text are provided here.

|  | OD, First | | OD, Last | | OS, First | | OS, Last | |
| --- | --- | --- | --- | --- | --- | --- | --- | --- |
| Subject | x, y | radius | x,y | radius | x,y | radius | x,y | radius |
| mf87157 | 2.57, -0.21 | 8.96 | 0.50, -0.50 | 4.15 | 7.50, -0.10 | ˖6.56 | 0.50, -0.50 | 4.15 |
| mf87158 | 3.39, 4.18 | 7.38 | 2.25, 4.50 | 3.39 | -10.5, 10.5 | 1.69 | 2.25, 4.50 | 3.39 |
| mf87164 | -1.50, 0.00 | ˖4.15 | -1.50, 0.00 | ˖4.15 | 0.00, 9.90 | ˖5.35 | -1.50, 0.00 | ˖4.15 |
| mf87167 | 0.27, -5.73 | 7.94 | 3.50, -6.67 | 7.18 | -2.61, -3.17 | 8.79 | 3.50, -6.67 | 7.18 |
| mf87169 | -0.06, 0.94 | 11.7 | 1.50, 0.87 | 11.1 | -0.64, 0.21 | 12.7 | 1.50, 0.87 | 11.1 |
| mf87172 | -0.40, 0.10 | ˖13.1 | -0.47, -0.22 | ˖13.2 | -1.12, -0.25 | 12.6 | -0.47, -0.22 | ˖13.2 |
| mf87174 | -0.77, 2.47 | 10.3 | -0.60, -1.20 | 10.7 | 0.97, -2.91 | 9.87 | -0.60, -1.20 | 10.7 |
| mf87177 | -3.82, 3.53 | 9.42 | -4.50, 10.5 | 3.78 | -7.95, 1.35 | 7.57 | -4.50, 10.5 | 3.78 |
| mf87171 | 2.14, 0.93 | 11.6 | 0.00, 0.00 | ˖13.5 | 2.33, 1.17 | ˖10.2 | 0.00, 0.00 | ˖13.5 |

*The x,y positions of the centroids of the oedema and the characteristic radii as in degree. See text of Fig. 6 for an explanation of the steps in the calculations. Radii with a ˖ indicate partial involvement of the fovea.

**Figure 6**. **A to D** are based on the 8×8 OCT thickness grid (Fig. 2A). Yellow regions are thicker than normal. The red * is the centroid of the single largest contiguous region of oedema and the red circle is the Equivalent circular region. A,B and C,D are pairs of data sets from the first and last visit for a right and a left eye of two subjects. In these cases the off-axis oedema tends to follow outer radii of the central pit, but never involving the fovea. **E.** Shows vectors representing the position of the 36 centroids in visual space which extend beyond 10 degrees eccentricity. **F.** The same data shown as a histogram. **G.** The radii of the Equivalent circular regions. **H.** The areas of the single largest contiguous area of oedema from each retina and visit.

We also added the following text to the Results which now reads:

“To discover more about the eyes that showed significant change in Figure 5 we quantified the differences from normal thickness of the OCT 8×8 thickness grid data (Fig. 2A) for the first and the last visit of each eye. Figure 6 A to D show examples of the resulting regions of oedema (yellow). We used the Matlab the *regionprops* function to find the single largest contiguous region of oedema, and found its centroid (x,y location), its area and a characteristic radius of the oedematous patch. Fig. 6E to G summarise the locations and extents of the off-axis oedematous patches. Table 3 shows the centroids and characteristic radii. As shown by Fig. 6 the off-axis oedema can partially surround the fovea without involving it. Only 4 eyes had complete foveal involvement on the later visit (mf87172, and mf87171).”
