# Supplementary material for: Objective perimetry identifies regional functional progression and recovery in mild Diabetic Macular Oedema
Source: PLoS One. 2023 Jun 15;18(6):e0287319. doi: 10.1371/journal.pone.0287319 (PMC10270604; doi:10.1371/journal.pone.0287319)
Supplement: Attachment — Submitted filename: Response to Reviewers_v7 final.docx [file pone.0287319.s010.docx]

**RESPONSE TO REVIEWER’S COMMENTS**

Reviewer Comments:

Reviewer #2: I am pleased that authors have taken efforts to address these key points.

The new panels A and B (Fig 6) shows the changes in oedema seem like a mirror image of change in OCT retinal thickness in the left most panel (Fig 5). This is probably due to flipping of data to represent the corresponding region in visual space.

Please confirm that if the data in Fig 6 is indeed in retinal space.

Therefore, add a note in Fig 6 legend that if the data is presented here in retinal space.

Panel E legend not accurate. It shows all centroids not just those greater than 10 degrees

Thank you for this comment. No, Figure 6 A-D are presented as left eyes and not presenting as corresponding regions in retinal space. They were flipped left right so that we could measure the median values across all eyes. It is also worth noting the Fig 5B3 is derived from the fitted slopes not the edema compared to normative data on the first and last days, like Fig 6, so they would not be expected to be similar. To clarify this in the text we have added the following statement to the Results:

“To discover more about the eyes that showed significant change in Figure 5 we quantified the differences from normal thickness of the OCT 8×8 thickness grid data (Fig. 2A) for the first and the last visit of each eye. Figure 6 A to D show examples of the resulting regions of oedema (yellow) presented as left eyes (flipped left-right). We used the Matlab the *regionprops* function to find the single largest contiguous region of oedema, and found its centroid (x,y location), its area and a characteristic radius of the oedematous patch. Fig. 6E to G summarise the locations and extents of the off-axis oedematous patches. Table 3 shows the centroids and characteristic radii. As shown by Fig. 6 the off-axis oedema can partially surround the fovea without involving it. Only 4 eyes had complete foveal involvement on the later visit (mf87172, and mf87171).

Figure 6E. presents all the centroids but to enhance visualisation of the central ones we presented the same data as a histogram in Fig 6F. We had confused the situation by leaving out the word “can”, which is now inserted. To make this clearer in the text we have modified the Fig 6. Legends to read:

**Fig 6**. **A to D** are based on the 8×8 OCT thickness grid (Fig. 2A). Yellow regions are thicker than normal. The red * is the centroid of the single largest contiguous region of oedema and the red circle is the Equivalent circular region. A,B and C,D are pairs of data sets from the first and last visit for a right and a left eye of two subjects. In these cases the off-axis oedema tends to follow outer radii of the central pit, but never involving the fovea. **E.** Shows vectors representing the position of the 36 centroids in visual space which can extend beyond 10 degrees eccentricity. **F.** The same data in Fig. 6E, shown as a histogram. **G.** The radii of the Equivalent circular regions. **H.** The areas of the single largest contiguous area of oedema from each retina and visit.
